# Rational Identification of Novel Antibody‐Drug Conjugate with High Bystander Killing Effect against Heterogeneous Tumors

**DOI:** 10.1002/advs.202306309

**Published:** 2024-01-25

**Authors:** Yu Guo, Zheyuan Shen, Wenbin Zhao, Jialiang Lu, Yi Song, Liteng Shen, Yang Lu, Mingfei Wu, Qiuqiu Shi, Weihao Zhuang, Yueping Qiu, Jianpeng Sheng, Zhan Zhou, Luo Fang, Jinxin Che, Xiaowu Dong

**Affiliations:** ^1^ Hangzhou Institute of Innovative Medicine, Institute of Drug Discovery and Design, College of Pharmaceutical Sciences Zhejiang University Hangzhou 310058 P. R. China; ^2^ Innovation Institute for Artificial Intelligence in Medicine of Zhejiang University Hangzhou 310018 P. R. China; ^3^ The Department of Pharmacy Zhejiang Cancer Hospital Hangzhou 310022 P. R. China; ^4^ Department of Hepatobiliary and Pancreatic Surgery the First Affiliated Hospital, Zhejiang University School of Medicine Hangzhou 310002 P. R. China; ^5^ Cancer Center Zhejiang University Hangzhou 310058 P. R. China; ^6^ Department of Pharmacy Second Affiliated Hospital Zhejiang University School of Medicine Hangzhou 310009 P. R. China

**Keywords:** antibody‐drug conjugates, bystander‐killing effect, camptothecin derivatives, rational design, tumor heterogeneity

## Abstract

Bystander‐killing payloads can significantly overcome the tumor heterogeneity issue and enhance the clinical potential of antibody‐drug conjugates (ADC), but the rational design and identification of effective bystander warheads constrain the broader implementation of this strategy. Here, graph attention networks (GAT) are constructed for a rational bystander killing scoring model and ADC construction workflow for the first time. To generate efficient bystander‐killing payloads, this model is utilized for score‐directed exatecan derivatives design. Among them, Ed9, the most potent payload with satisfactory permeability and bioactivity, is further used to construct ADC. Through linker optimization and conjugation, novel ADCs are constructed that perform excellent anti‐tumor efficacy and bystander‐killing effect in vivo and in vitro. The optimal conjugate T‐VEd9 exhibited therapeutic efficacy superior to DS‐8201 against heterogeneous tumors. These results demonstrate that the effective scoring approach can pave the way for the discovery of novel ADC with promising bystander payloads to combat tumor heterogeneity.

## Graphical abstract

A rational strategy for generating antibody‐drug conjugate with bystander‐killing effect through GAT‐driven bystander‐killing scoring, payload screening and linker optimization. Several potential bystander payloads and conjugates are discovered under the strategy, in which the optimal conjugate T‐VEd9 exhibited therapeutic efficacy superior to DS‐8201 against heterogeneous tumors.

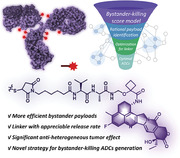

## 1 Introduction

Antibody‐drug conjugates (ADCs) consisting of monoclonal antibodies (mAbs), linkers, and payloads are an increasingly important class of novel therapeutics that combine tumor‐targeted therapeutics with cancer chemotherapy drugs.^[^ ^1^, ^2^, ^3^ ^]^ ADCs take advantage of the high specificity of mAbs and the potent activity of cytotoxic warheads.^[^ ^4^, ^5^, ^6^, ^7^ ^]^ Up to now, 15 approved ADCs and more than 150 ADCs in clinical trials (clinicaltrials.gov) have demonstrated their clinical potential, marking that ADCs are becoming the research focus of novel anti‐tumor therapies. ^[^ ^8^, ^9^, ^10^ ^]^ Tumor heterogeneity, including inter‐tumor and intra‐tumor heterogeneity, as one of the characteristics of malignant tumors, is the main factor leading to differences in tumor growth, invasion, metastasis, and prognosis.^[^ ^11^, ^12^, ^13^ ^]^ An essential challenge for ADC therapy is the heterogeneous expression of target antigens in tumor tissues or metastases, which makes it difficult for ADC to achieve the expected efficacy.^[^ ^14^, ^15^ ^]^

The bystander‐killing effect of ADC depends on the released permeable payload in the tumor microenvironment to kill tumor cells with low or even negative antigen expression. This is the case for human epidermal receptor 2 (HER2), a classic biomarker only overexpressed in less than 20% of breast cancer patients.^[^ ^16^, ^17^, ^18^ ^]^ The inter‐tumor heterogeneity significantly compresses the treatment space for HER2‐targeted therapies.^[^ ^19^, ^20^ ^]^ Even among HER2‐positive breast cancer patients, approximately 30% of patients exhibit intra‐tumor heterogeneity in HER2 expression.^[^ ^21^, ^22^, ^23^ ^]^ As previously reported, tumors with heterogeneous HER2 expression did not benefit significantly from Trastuzumab emtansine (Kadcyla, T‐DM1) but showed solid response rates with trastuzumab deruxtecan (Enhertu, DS‐8201, T‐DXd).^[^ ^24^ ^]^ Even more exciting, T‐DXd exhibited response rates of 30–40% in breast tumors with low HER2 expression, a setting where T‐DM1 had limited effect.^[^ ^25^, ^26^ ^]^ Unlike previous ADCs, T‐DXd benefits from the stable tetrapeptide linker and the DX‐8951f derivative (DXd) payload with a high homogeneity drug‐antibody ratio (DAR) of 8, which reveals the potential of payload iteration in ADC therapy.^[^ ^27^, ^28^ ^]^ It is worth noting that a previous study on T‐DXd attributed the high potency to changes in payloads’ permeability due to their different lipophilicity, which is associated with the bystander‐killing effect of ADCs.^[^ ^27^ ^]^ Recently, Disitamab Vedotin (Aidixi, RC‐48), composing the classic bystander‐killing payload monomethyl auristatin E (MMAE), demonstrated significant efficacy against HER2‐low‐expressing urothelial carcinoma in phase II clinical trial [ORR = 38% (5/13) in patients with HER2(IHC 1+)].^[^ ^29^ ^]^ The bystander‐killing effect conferred by membrane‐permeable toxins has become a potential method for ADCs to treat heterogeneous tumors.^[^ ^30^ ^]^ Yamazaki et al. successfully constructed dual‐payload ADCs carrying MMAE and monomethyl auristatin F (MMAF) and attributed their significant therapeutic effect against HER2 heterogeneous tumors to the bystander effect of MMAE.^[^ ^31^ ^]^ Although several ongoing clinical trials have revealed the potential of novel permeable payloads in treating heterogeneous tumors,^[^ ^32^, ^33^ ^]^ the lack of comprehensive method for rational identification of bystander payloads and ADCs limited the clinical potential of this strategy.

Here we show our strategy for the discovery of ADC with a more potency bystander‐killing warhead. Based on the previous reports and the analysis of existing payloads, we applied graph attention network (GAT) and comprehensive molecular characterizations to bystander‐killing scoring model for payloads’ rational identification for the first time. Then, we obtained several efficient bystander‐killing exatecan derivative payloads by score‐guided molecule generation and screening. Through further linker optimization, we constructed novel ADCs that performed excellent anti‐tumor efficacy in vivo and in vitro based on the most potent payload Ed9. We also demonstrated that a homogeneous anti‐HER2 ADC containing the novel payload exhibits a significant therapeutic effect in xenograft models bearing the heterogeneous HER2 expression tumor. Notably, the optimal conjugate, T‐VEd9, did not exhibit systemic toxicity while showing a greater in vivo curative effect than existing HER2 ADCs at the same dose. Our results suggest that the rational optimization strategy based on the GAT‐driven scoring model could provide experience for generating novel bystander payloads. The high‐efficiency bystander‐killing ADCs based on these novel permeable warheads are a promising approach to combat tumor heterogeneity.

## 2 Results and Discussion

### 2.1 The GAT‐Driven Bystander Score for Rational Payload Design

During the decades of ADC development, as the “small” elements in the “large” ADC molecules, the properties other than cytotoxicity of payloads are of increasing interest to researchers.^[^ ^34^ ^]^ Benefiting from our ongoing ADC database work,^[^ ^35^ ^]^ we found a correlation between IC_50_ and calculated cLogD of ADC payloads currently in clinic or development (Figures S1 and S2A, Supporting Information),^[^ ^36^ ^]^ consistent with previous reports.^[^ ^27^ ^]^ Recently, a study demonstrated that the lipophilic conjugation strategy can significantly enhance the cell permeability and bioactivity of molecules.^[^ ^37^ ^]^ This, in turn, has inspired many researchers to utilize cLogD as a reference point for the rational design of potent and permeable payloads for ADCs with the bystander killing effect. In practice, however, utilizing a single physicochemical property derived from calculations as the permeable descriptor is rather simplistic as it overlooks some vital molecular characteristics of the payload, particularly for those with complex skeleton structures (e.g., Lys‐SMCC‐DM1 and MMAF versus DXd and SN‐38, Figure S2B, Supporting Information). Consequently, we employed graph attention networks and more comprehensive molecular characterizations to build bystander scoring (B score) model for rational bystander payload design.

**Figure 1:**
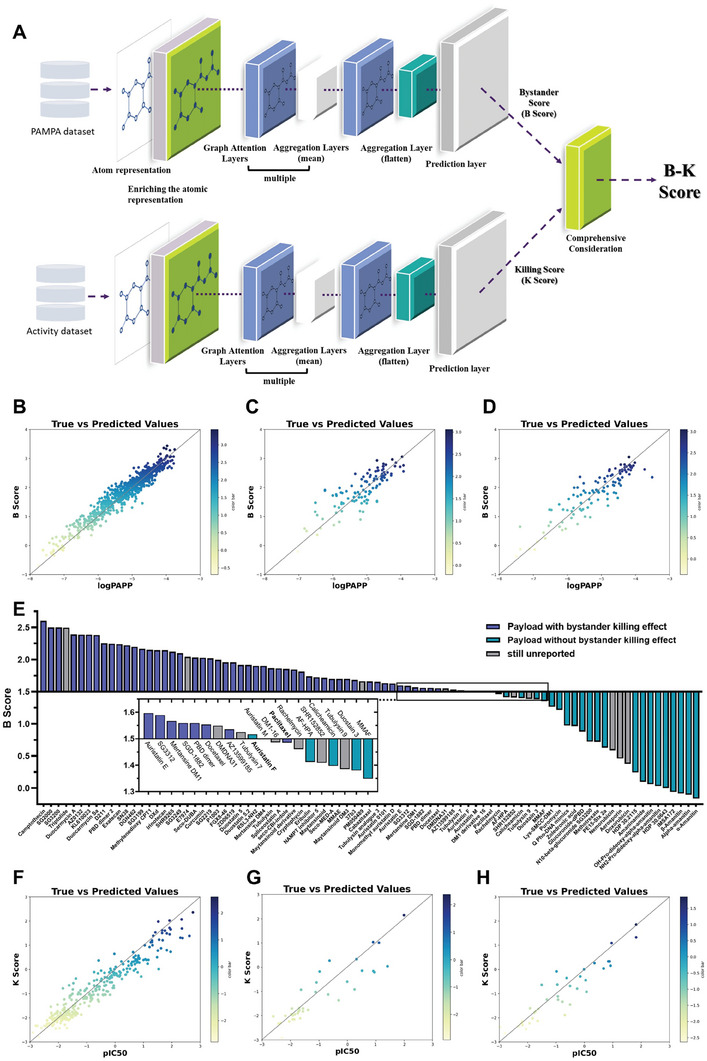
The construction and validation of the GAT‐driven bystander‐killing score model. A) The architecture of the model is divided into two sections, each consisting of a molecular characterization layer, multiple GAT layers, aggregation layers, and a prediction layer. These sections are identical in structure but process different input data. Finally, the scores calculated by these two sections are comprehensively considered and serve as the final B‐K score. B–D) The performance of the B score model on the (B) train, C) validation, and D) test dataset. E) The prediction tests of the B score model for payload currently in clinic or development from ADCdb, value 1.5 in B score model is the dividing line for with and without bystander effect. (F‐H) The performance of the K score model on the F) train, G) validation, and H) test dataset.

The schematic representation of the network architecture is illustrated in **Figure** **1**A. We implemented the entire network using the Deepchem and PyTorch Geometric frameworks with the aim of predicting the permeability of compounds. The model architecture features multiple graph attention layers, which enable the model to learn the importance of neighboring nodes in the molecular graph representation. In addition, the architecture includes a dense layer and a dropout layer at the end of the model for prediction purposes. This design allows the selective aggregation of information from the most relevant neighboring nodes, leading to a better understanding of the underlying molecular properties.

The membrane permeability dataset was normalized and then divided into training, validation, and test sets using a random splitter,^[^ ^38^ ^]^ with the respective proportions of 8:1:1, and a normalization transformer was applied to preprocess the target values. To optimize the model's hyperparameters, Optuna was performed searching over learning rate, weight decay, number of epochs, and batch size. The model was then trained using the optimal hyperparameters and evaluated using the Pearson R^2^ score (Figure 1B‐D). It is worth mentioning that we used the data set of Caco‐2 assay for model training, in cases where LogP_app_ (cm s^−1^) > −5.5 [LogP_app_ (nm s^−1^) > 1.5] measured in this assay is considered a necessary indicator of passive diffusion,^[^ ^39^, ^40^ ^]^ we set a B score of 1.5 as a criterion for whether payload has a potential bystander effect.

In the final evaluation, the model achieved an impressive R^2^ score of 0.811 on the test set (Figure 1D). To compare our model with the baseline approach, we further performed training on traditional machine learning models, including Support Vector Machine (SVM), Extreme Gradient Boosting (XGBOOST), BayesianRidge, and Random Forest (RF), using Morgan molecular fingerprints to featurelize the same dataset. The parameters of the different models were also optimized with Optuna. As a result, our model achieved the highest R^2^ values compared to others, demonstrating superior performance (Table S1, Supporting Information). More importantly, the GAT‐driven B Score model maintains high accuracy in the bystander effects prediction of over 80 ADC payloads currently in clinic or development from ADCdb (http://adcdb.idrblab.net/) (Figure 1E; Table S2, Supporting Information),^[^ ^35^ ^]^ which is a totally external dataset not included in our initial training, validation, and test sets, demonstrating the model's robustness and generalizability in real‐world applications. This result indicates that the model demonstrates a strong correlation between predicted and actual permeability values, showcasing its effectiveness in identifying underlying molecular properties and predicting bystander effects for our further payload design and optimization.

### 2.2 Discovery of Bystander‐Killing Exatecan Derivatives (Eds)

**Figure 2:**
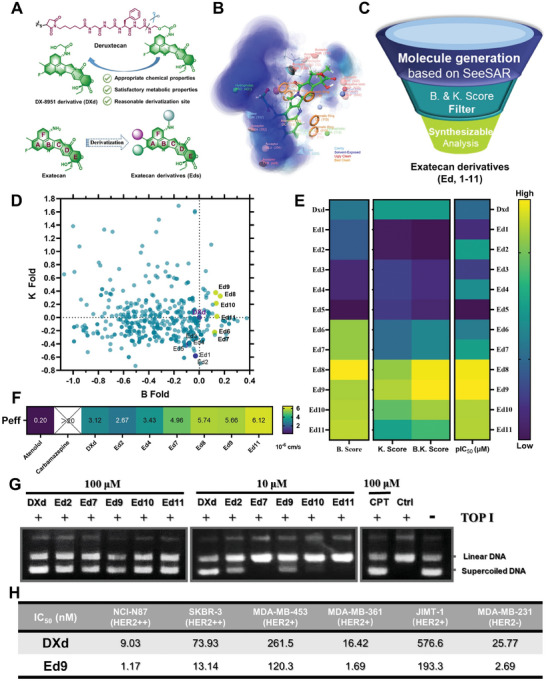
Design and evaluation of novel Eds with membrane permeability. A) Schematic illustration of the structure and generation strategy exatecan derivatives (Eds). B) Modification site analysis by determining solvent exposure of DXd in DXd/Topo I/DNA complex. C) Generation and screening process of Eds. D) B score and K score of generated Eds, calibrated with DXd (0,0). E) The heat map of the B score, K score, B.K. score, and pIC_50_ of Eds and DXd. F) Passive cell membrane diffusion of Eds and DXd by a PAMPA assay with atenolol as a negative control and carbamazepine as a positive control. G) Topo I inhibitory activity of Eds and DXd at 10 µM and 100 µM, with CPT (100 µM) as positive control. H) Proliferation inhibitory activity of Ed9 and DXd against human cancer cell lines with different HER2‐expression. Tumor cells were treated with payloads for 3 days and cell viability (%) was calculated. Data shown are representative of more than two independent duplicates.

In recent years, camptothecin derivatives, a class of chemotherapeutic drugs acting on topoisomerase I (Topo I), have achieved unprecedented success as ADC payloads, and their moderate lipophilicity and molecular weight are suitable for further derivatization and payload development. More importantly, the unique metabolic properties of the camptothecin backbone can help the cell‐permeable payload to circumvent its potential systemic toxicity.^[^ ^41^, ^42^ ^]^ Encouraged by this, we chose exatecan which contains an easily modified F‐ring for bystander derivation (**Figure** **2**A). Combined the binding analysis and growth space of DXd/Topo I/DNA ternary complex (Figure 2B), a series of A‐ring and F‐ring exatecan derivatives (Eds) were generated by SeeSAR Inspirator module (Figure 2A–C). Considering that the derivatization of the payload will also affect its killing effect, we adopted a similar GAT to construct a killing score (K score) model for predicting the Topo I inhibitory and optimized it with new hyperparameters using the related dataset (Figure 1A and 1F–H). Then, the generated Eds were screened by B‐K Score and synthesizable analysis (Figure 2C). Among them, some F‐ring amino‐substituted Eds (Ed6‐11) were selected as candidate payloads due to their excellent score and ease of synthesis (Figure 2D). On the other hand, some other F‐ring modified Eds (Ed1‐5) with chiral centers have also received our attention as stereoisomeric exploration and negative control for model validation. All Eds with different scores were synthesized by one‐step acid‐amine condensation of commercially available substituted glycolic acid with DX‐8951f, and their inhibition of tumor cell (SKBR‐3 and MDA‐MB‐231) proliferation was further evaluated (Figure 2C; Tables S3 and S4, Supporting Information).

Novel payloads are mainly divided into mono‐substituted and di‐substituted (including cycloalkyl) hydroxyacetyl‐modified Eds, and as expected, the proliferation inhibitory activity of Eds showed a strong correlation with predicted B.K. score (Figure 2E; Tables S3 and S4, Supporting Information). Although mono‐substituted hydroxyacetyl‐modified Eds did not exhibit satisfactory proliferation inhibitory activity, an interesting phenomenon was observed: the chirality of the substituted hydroxyacetyl group seems to affect the potency of these derivatives. Among the chiral isomers of single‐substituted Eds, the R‐isomer is always dominant (Ed2, 4, 7 versus Ed1, 3, 6, Table S4, Supporting Information), which suggests that the R isomer‐substituted hydroxyacetyl‐modified Eds may have more therapeutic potential. As predicted by B‐K score, the dimethyl and cycloalkyl‐substituted hydroxyacetyl‐modified Eds (Ed8‐Ed11, Figure 2E; Table S4, Supporting Information) exhibited satisfactory antitumor activity with the IC_50_ at nM level. Although Ed8‐Ed11 have both satisfactory bystander scores and killing scores, the two scores of them seem to be mutually exclusive. We speculated that although bulky hydrophobic substituents may enhance the membrane permeability of Eds, they may affect the binding of Eds to Topo I/DNA and result in potency reduction. Therefore, we further evaluated some Eds’ membrane permeability and Topo I inhibitory activity. The membrane permeability of the Eds was tested by Parallel Artificial Membrane Permeation Assay (PAMPA), and the results showed that with the introduction of lipophilic groups, the membrane permeability of high B score Eds was significantly improved compared with that of DXd (Figure 2F). On the other hand, Topo I‐mediated DNA cleavage assay demonstrate that the modification of hydroxyacetyl group will affect the Topo I inhibitory activity of Eds to a certain extent, and this effect will become more significant with the increase of substituted cycloalkyl volume (Figure 2G). A typical case is that Ed10 and Ed11 lose their inhibitory activity on Topo I at 10 µM. Inspired by this, we designed and synthesized two molecules with high cLogD and reactant accessibility that are outside the range of generating sets (Figure S3A, Supporting Information). The results of scoring and testing show that B‐K score is far more accurate than cLogD in guiding the design of this series of compounds (Figure S3B, Supporting Information). Although the inhibitory activity of Ed9 against Topo I was significantly lower than that of DXd at the concentration of 10 µM, it still maintained a satisfactory proliferation inhibitory activity, which may benefit from its enhanced membrane permeability. Overall, in accordance with our B‐K score, Ed9 demonstrates a well‐balanced bystander effect and killing effect, which sets it apart from all other Eds.

Furthermore, we compared Ed9 and DXd on multiple tumor cell lines with different expression levels of HER2. The IC_50_ values of Ed9 against NCI‐N87, SKBR‐3, MDA‐MB‐453, MDA‐MB‐361, JIMT‐1, and MDA‐MB‐231 are 2–10 fold lower than those of DXd (Figure 2H), indicating that substitution in the hydroxyacetyl by a cyclobutyl group significantly increases the permeability and activity. Therefore, we conclude that Ed9 possesses superior physicochemical properties and biological activities, which can be used as a potential high‐efficiency bystander‐killing payload for further study.

### 2.3 Linker Optimization, Construction, and Characterization of Ed9 ADCs

**Figure 3:**
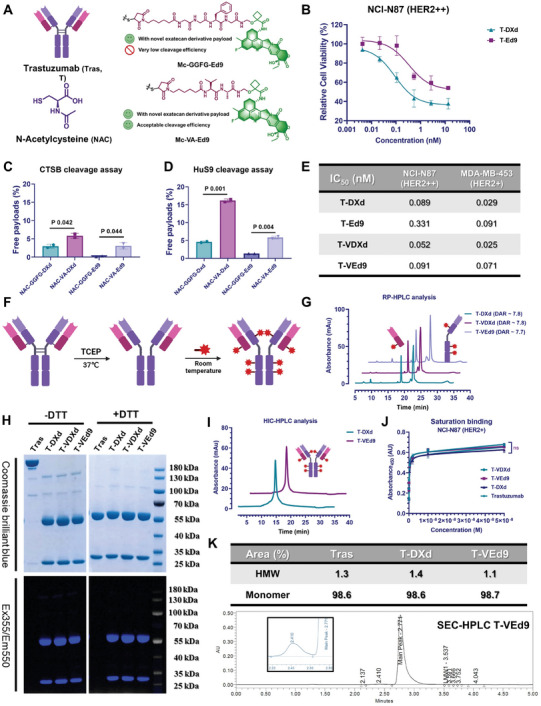
Linker optimization, construction and characterization of Ed9 ADCs. A) Schematic illustration of the linker optimization strategy and structure of the Gly‐Gly‐Phe‐Gly and Val‐Ala linker conjugates. B) Proliferation inhibitory activity of ADCs against NCI‐N87 cells. Tumor cells were treated with ADCs for 5 days and relative cell viability (%) was calculated. C) Human cathepsin B‐mediated and D) Human liver S9‐mediated cleavage of NAC‐linker‐payloads (NAC‐L‐P) at 37°C. Cleavage of each probe was monitored by HPLC and LC/ESI‐MS. E) The half‐maximal inhibitory concentration (IC_50_) of ADCs with different L‐Ps. F) Schematic illustration of the ADC conjugation through the inter‐chain disulfide reduction and classic Michael addition. G) Reversed‐phase high‐performance liquid chromatography (RP‐HPLC) of DAR8 ADCs. Absorbance wavelength was 280 nm. H) SDS‐PAGE analysis of Trastuzumab, T‐DXd, T‐VDXd, and T‐VEd9. I) Hydrophobic interaction chromatography (HIC) of DAR8 ADCs. Absorbance wavelength was 280 nm. J) Saturation‐binding curves obtained by NCI‐N87 (HER2+) cell‐based ELISA. K) Size‐exclusion chromatography (SEC) of DAR8 ADCs. Absorbance wavelength was 280 nm. Isomer ratio was calculated by absorbance area. DAR, drug‐to‐antibody ratio. All data shown are representative of more than two independent duplicates. Error bars represent S.D. Curve fitting and IC_50_ calculation was performed using GraphPad Prism 9.3 software.

Effective bystander killing of ADCs also relies on the cleavage of the linker and the release of the payload at the target tissue. In view of the previously reported satisfactory performance of the Gly‐Gly‐Phe‐Gly (GGFG) tetrapeptide linker used by T‐DXd, we synthesized Mc‐GGFG‐Ed9 using Ed9 as the payload and constructed a novel ADC (Tras‐GGFG‐Ed9, T‐Ed9) based on it (**Figure** **3**A). Unfortunately, although Ed9 was the more effective warhead, T‐Ed9 showed a weaker proliferation inhibitory activity than T‐DXd at the same drug‐to‐antibody ratio (DAR) (Figure 3B). We speculate that the low potency may be caused by the unsatisfactory linker cleavage and payload release due to the cyclobutyl group substitution close to the linker's cleavage site. Cathepsin B (CTSB) is widely considered to be the major cleavage enzyme for the peptide linkers of existing ADCs (Figrue S4, Supporting Information).^[^ ^43^, ^44^ ^]^ Therefore, the CTSB cleavage assay was performed to evaluate the response function of the novel payload and linker composition. As we speculated, less than 1% released payload was detected after human liver CTSB co‐incubated with NAC‐GGFG‐Ed9 for 24 h (Figure 3C), demonstrating that the payload Ed9 is not compatible with the tetrapeptide linker. As a more general cathepsin substrate, Val‐Ala has been widely used in the construction of novel ADCs in recent years, which can be cleaved by more than one cathepsin expressed in various tumors.^[^ ^3^, ^45^, ^46^ ^]^ Therefore, we constructed a Val‐Ala‐based dipeptide linker‐payload fragment, which exhibited better payload release efficiency than the GGFG linker in the CTSB cleavage system (Figure 3C). Although the introduction of the cyclobutyl group still had a certain effect on linkers’ cleavage, compared with NAC‐GGFG‐Ed9, the release rate of NAC‐VA‐Ed9 (3.07±0.87%) recovered to a level similar to NAC‐GGFG‐DXd (2.99±0.54%). Human liver S9 (HuS9), as a more complex mixture system, has recently been proven to simulate more realistic cleavage conditions in the analysis of ADC payload release.^[^ ^47^, ^48^ ^]^ Based on this, we evaluated the release efficiency of the abovementioned linker‐payload composition using the HuS9 cleavage assay. The results showed that the cleavage system of HuS9 was more “violent” than that of CTSB, but the release efficiency of NAC‐GGFG‐Ed9 was still low (less than 2%, Figure 3D). Although the cyclobutyl effect on warhead release was more pronounced in the HuS9 assay, it was still rescued by the introduction of the VA motif (Figure 3D). Further, we constructed two novel ADCs, Tras‐VA‐DXd (T‐VDXd) and Tras‐VA‐Ed9 (T‐VEd9) and evaluated their inhibitory activity on the proliferation of HER2‐positive tumor cells (Figure 3E). The results showed that T‐VEd9 performed superior proliferation inhibitory activity compared to T‐Ed9, which further proved the effectiveness of the VA strategy.

To obtain homogeneous DAR‐8 ADCs above, maleimide‐linker‐payloads were conjugated to fully reduced trastuzumab through the inter‐chain cysteines using a reported bioconjugation protocol (Figure 3F).^[^ ^49^ ^]^ The efficiency and homogeneity of the conjugates were confirmed through analytical characterization using reversed‐phase high‐performance liquid chromatography (RP‐HPLC, Figure 3G). The bioconjugation yields were 70% or higher for all ADCs, with the DAR close to 8 calculated by absorbance peak area. The successful conjugation and the purity of conjugates was further confirmed through Coomassie Blue staining and camptothecin backbone characteristic fluorescence detection after SDS‐PAGE (Figure 3H). The purity and homogeneity of DAR‐8 ADC was also verified by hydrophobic interaction chromatography (HIC, Figure 3I), which was consistent with the results of MALDI‐TOF/TOF MS analysis (Figure S5, Supporting Information). To demonstrate that the affinity and specificity of trastuzumab were not significantly affected by the conjugation of the linker‐payload, both HER2‐positive and negative cell‐based ELISA were performed to determine ADCs’ binding affinity. The results revealed that while each ADC bound to HER2‐positive NCI‐N87 cells with a Kd similar to that of unconjugated trastuzumab (2.69 nM, Figure 3J), none of the ADCs showed specific binding affinity to HER2‐negative MDA‐MB‐231 cells (Figure S6A, Supporting Information). In view of the possible effect of more lipophilic payloads on antibody stability, we tested the potential polymerization of the conjugates. Size‐exclusion chromatography (SEC) analysis revealed that compared with T‐DXd, T‐VEd9 with lipophilic payload avoided possible multimerization and mainly existed in monomer form (> 98.5%, Figure 3K). To our delight, the use of the VA motif seems to circumvent the negative effects on conjugates’ stability of the novel payload, possibly because it avoids hydrophobic Phe in the GGFG linker (HMW_T‐Ed9_ = 3.2%, Figure S7, Supporting Information). Next, the stability of the ADCs in human and mouse plasma was evaluated. After incubation in human plasma at 37°C for 7 days, none of the ADCs showed significant payload release, while the payload release was detected during co‐incubation with mouse plasma (Figure S6B, Supporting Information), possibly due to the previously reported carboxylesterase 1c (Ces1c).^[^ ^50^, ^51^ ^]^ However, the payload release rates of all tested ADCs in mouse plasma were acceptable and stabilized at about 2%, suggesting that the ADCs had considerable plasma stability.

### 2.4 In Vitro Evaluation of the Bystander‐Killing Effect

**Figure 4:**
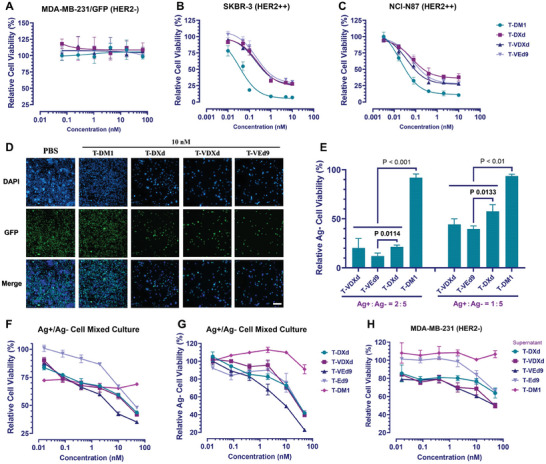
In vitro evaluation of ADCs. Proliferation inhibitory activity of ADCs against A) MDA‐MB‐231/GFP (HER2‐) cells, B) SKBR‐3 (HER2+) cells, and C) NCI‐N87 (HER2+) cells. Tumor cells were treated with ADCs for 5 days and relative cell viability (%) was calculated. D) Fluorescence imaging of NCI‐N87 and MDA‐MB‐231/GFP (Green) co‐culture system. the nuclei were stained with DAPI (Blue). Scale bar: 25 µm. E) Proliferation inhibitory activity of ADCs against MDA‐MB‐231/GFP (HER2‐) cells in the co‐culture system. The relative cell viability (%) was calculated by measuring GFP fluorescence intensity. F) Proliferation inhibitory activity of ADCs against both HER2+ and HER2‐ cells in the co‐culture system, error bars represent S.E.M. G) Proliferation inhibitory activity of ADCs against HER2‐ cells in the co‐culture system, error bars represent S.E.M. H) Proliferation inhibitory activity against HER2‐ cells of HER2+ cells supernatant cultured with ADCs, error bars represent S.E.M. All data shown are representative of more than two independent duplicates. Error bars represent S.D. without additional annotation. Curve fitting and IC_50_ calculation was performed using GraphPad Prism 9.3 software.

In order to determine whether T‐VEd9 induces more effective bystander killing, a co‐culture cell killing assay was performed. We constructed MDA‐MB‐231 cells with stable expression of GFP to determine the amount of HER2‐negative cells in the co‐incubation system. Single‐cell lines with different HER2 expressions (MDA‐MB‐231/GFP, SKBR‐3, and NCI‐N87) were exposed to gradient concentrations of T‐DM1, T‐DXd, T‐VDXd, or T‐VEd9 to determine their sensitivity to HER2‐targeting ADCs. After 6 days of incubation, relative cell viability was measured by the cell counting kit‐8 assay. As expected, the proliferation of HER2‐positive cells was significantly inhibited by the four ADCs with the IC_50_ less than 1 nM (**Figure** **4**B,C), to which monoculture MDA‐MB‐231/GFP cells displayed no sensitivity to the four ADCs at the concentration of more than 10 nM (Figure 4A). Subsequently, we conducted the co‐culture of HER2‐positive and HER2‐negative cell lines at varying ratios,^[^ ^52^ ^]^ followed by treatment with the abovementioned ADCs. The viability of HER2‐negative cells in the co‐culture system was measured by detecting the GFP fluorescence intensity. The results showed that three ADCs with exatecan derivatives payload killed both NCI‐N87 and MDA‐MB‐231/GFP cells, but T‐DM1 (negative control) did not, which was consistent with the result observed by fluorescence microscopy (Figure 4D; Figure S8, Supporting Information). Since T‐VEd9 has HER2‐specific cytotoxicity, the released payload Ed9 can cause the killing of MDA‐MB‐231 cells, suggesting that T‐VEd9 has a bystander‐killing effect. Notably, this effect of T‐VEd9 was more pronounced than that of T‐DXd, especially at a cell ratio of 1:5 (HER2+ NCI‐N87: HER2‐ MDA‐MB‐231/GFP) (Figure 4E),^[^ ^27^, ^53^ ^]^ indicating that the membrane‐permeable payload Ed9 is crucial for the bystander‐killing effect of antibody‐drug conjugates. Although T‐VDXd also exhibited superior effects over T‐DXd, the bystander‐killing effect of T‐VEd9 was more significant and further evaluated as the optimal conjugate due to the improvement of both linker kinetics and payload permeability. Furthermore, we performed the concentration‐dependent proliferation inhibitory evaluation of ADCs against mixed NCI‐N87 and MDA‐MB‐231/GFP cells. As expected, the proliferation inhibition of T‐VEd9 was significant in both mixed cells and HER2‐ cells (Figure 4F,G). The supernatant of Eds‐ADC co‐incubated with HER2+ NCI‐N87 cells for 48 h showed considerable proliferation inhibitory activity against HER2‐ MDA‐MB‐231 cells (Figure 4H), further proving the bystander killing potential of T‐VEd9.

### 2.5 In Vivo Evaluation of the Bystander‐Killing Effect on HER2‐Heterogeneity CDX Model

**Figure 5:**
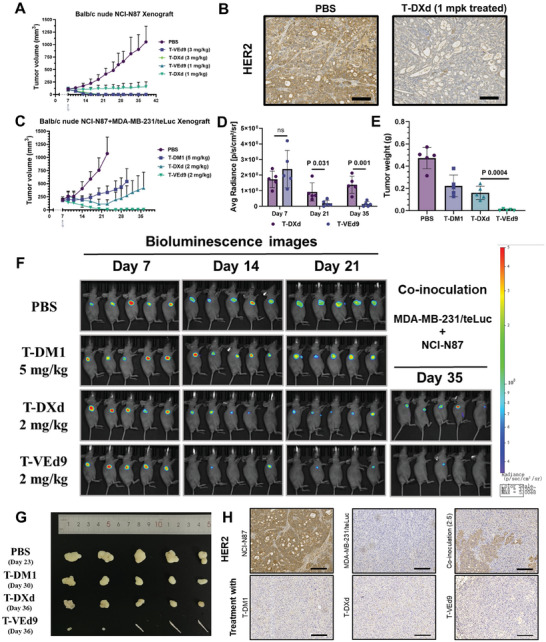
In vivo evaluation of ADCs. A) Anti‐tumor activity in HER2+ NCI‐N87 gastric cancer model following a single intravenous ADC dose of 1 or 3 mg kg^−1^. B) HER2 expression of remaining tumors after treatment with PBS and T‐DXd (1 mg k^−1^g). Scale bar: 100 µm. C–F) Bystander killing in co‐inoculation xenograft model following a single intravenous ADC dose of 2 or 5 mg kg^−1^. Luciferase activity was detected by in vivo imager after intraperitoneal injection of substrate. C) Tumor volume change. D) Luciferase activity. E) Tumor weight. F) Bioluminescence imaging data of luciferase activity. G) Tumors collected at treatment endpoint of PBS control and each ADC. H) HER2 expression on tumors consisting of either HER2‐positive or HER2‐negative cells, co‐inoculation (2: 5 ratio at the time of implantation) and the remaining and regrown tumors after treatment with each ADC. Scale bar: 100 µm. All data shown are representative of more than two independent duplicates. Error bars represent S.D. Curve fitting and *p*‐value calculation was performed using GraphPad Prism 9.3 software.

Encouraged by the above finding, we evaluated T‐VEd9 for in vivo treatment efficacy in SKBR‐3 and NCI‐N87 cell‐driven xenograft models. As a first preliminary in vivo efficacy experiment, a single exploratory dose of each ADC (10 mg kg^−1^) or PBS control was injected into HER2+ breast cancer (SKBR‐3)‐bearing mice through the tail vein. No weight loss associated with systemic toxicity was observed during the study following the administration of either ADC (Figure S9, Supporting Information). T‐VEd9, the Ed9‐based ADC, was found to be curative with a single 10 mg kg^−1^ dose, and no signs of tumor recurrence were seen by the end of the study (Figure S9B–E, Supporting Information). Notably, the therapeutic effect of trastuzumab and an ADC with a non‐cleavable linker (T‐VAEd9, Figure S9A,B, and D, Supporting Information) was insignificant, suggesting that a cleavable linker is necessary for Eds‐ADCs. To further investigate potential distinctions in efficacy between the two ADCs of the study, antitumor pharmacodynamic studies on NCI‐N87 (gastric cancer, HER2+) cell‐driven xenograft model was carried out at the dose of 1 or 3 mg kg^−1^. At a dose of 3 mg kg^−1^ for each ADC, complete and prolonged remission of tumors was observed (**Figure** **5**A; Figure S10, Supporting Information), which was consistent with previous reports.^[^ ^28^ ^]^ T‐VEd9 demonstrated significant potency even at a lower dose (1 mg kg^−1^), whereas T‐DXd showed only partial inhibition of tumor growth despite its high in vitro cell proliferation inhibitory activity (Figure 5A; Figure S10A,B, Supporting Information). Immunohistochemistry (IHC) analysis of the residual tumor showed that the expression of HER2 decreased in some tissues after treatment with 1 mg kg^−1^ T‐DXd (Figure 5B), which was considered potentially related to HER2‐driven ADC resistance.^[^ ^14^, ^54^, ^55^ ^]^ In addition, we constructed a more challenging xenograft tumor model using JIMT‐1, the breast cancer cell line with lower HER2 expression and resistance to trastuzumab (Figure S13, Supporting Information).^[^ ^56^, ^57^ ^]^ A single dose of 5 mg kg^−1^ was chosen to evaluate the anti‐tumor activity of ADCs, and both T‐DXd and T‐VEd9 exhibited good tumor inhibitory effects two weeks after administration (Figure S11A,C, Supporting Information). However, as time went on, the tumors in some of the treated animals began to grow (Figure S11A–C, Supporting Information), which we attribute to lower doses and frequency than previously reported.^[^ ^28^ ^]^ Notwithstanding these challenges, the T‐VEd9 treatment group still achieved significant tumor suppression at the end of the experiment, with one mouse achieving complete tumor remission (Figure S11B,D, Supporting Information). Therefore, we believe that T‐VEd9 may have promising therapeutic potential under a reasonable dosing regimen.

Furthermore, we established a novel in vivo assessment system to evaluate the bystander‐killing effect observed in the abovementioned in vitro studies. Teal luciferase (teLuc) was an optimized luciferase with enhanced bioluminescence imaging paired with diphenylterazine (DTZ) as substrate.^[^ ^58^ ^]^ Given the satisfactory sensitivity and signal‐to‐noise ratio of teLuc/DTZ, we generated MDA‐MB‐231/teLuc cells for determining HER2‐negative cells in a mixed tumor containing both HER2‐positive and negative cells with the use of an in vivo imaging system. Then, we established a xenograft model of HER2 heterogeneity tumor comprising HER2‐positive NCI‐N87 cells and HER2‐negative MDA‐MB‐231/teLuc cells (2: 5 ratio) transferred into BALB/c nude mice. In most mice, the admixed tumor showed aggressive growth and reached a noticeable size of ≈180 mm^3^ within 7 days after co‐inoculation (Figure 5C). The heterogeneity of the mixed tumor was verified by the immunohistochemistry of HER2, as shown in Figure 5H, in which the co‐inoculation tumor exhibited both HER2‐positive and HER2‐negative cancer cells. Afterward, the co‐inoculated xenograft mice received each HER2‐targeting ADC treatment at a single dose, and the luciferase activity and tumor volume were measured once and thrice a week, respectively (Figure 5C,D, and 5F). Regarding tumor volume change, T‐DM1 and T‐DXd were less effective than T‐VEd9 (Figure 5C; Figure S12B, Supporting Information), which may be due to limited tumor elimination, especially limited bystander killing against antigen‐negative tumor cells. In the T‐DXd and T‐VEd9 treatment groups, a notable decrease in luciferase‐mediated bioluminescent signal was observed (Figure 5D,F), indicating a partial elimination of MDA‐MB‐231/teLuc cells. More complete regression of HER2‐negative tumors was achieved in T‐VEd9‐treated mice, while the T‐DXd group showed tumor regrowth after day 35, consistent with quantified average radiance (Figure 5D–F). Endpoint immunohistochemistry (IHC) analysis of tumor tissues in all treatment groups revealed the absence of HER2‐positive cells (Figure 5G,H), indicating that tumor recurrence in this model was mainly due to the proliferation of HER2‐negative MDA‐MB‐231 cells. Considering the more complex and challenging environment of the heterogeneous xenograft model, these results demonstrate that the novel exatecan derivative and cleavable linker system can fully stimulate the therapeutic potential of Eds‐ADCs, benefiting from the satisfactory tumor tissue permeability brought by the novel payload. Hematoxylin and Eosin (H&E) staining of liver and lung tissue after treatment showed no significant differences relative to the blank group at the end of the study, indicating no significant tissue toxicity caused by ADC treatment (Figures S12A and S14, Supporting Information). Further, we performed blood biochemical analysis to quantify enzymes related to liver function, namely aminotransferase (ALT and AST), alkaline phosphatase (ALP), and γ‐glutamyl transpeptidase (γ‐GT). The results showed that these indexes of the mice under T‐VEd9 treatment were within the normal range (Figure S15, Supporting Information), indicating that the novel ADC has almost no risk of causing liver and systemic toxicities. Recently, interstitial lung disease (ILD) has attracted increasing attention as a common adverse reaction of HER2‐ADC.^[^ ^59^, ^60^ ^]^ However, its mechanism of occurrence in not yet clear and has only been reported on primates. Thus, further ILD discussion of T‐VEd9 should be based on species‐appropriate animals and toxicology model evaluation which we will continue to investigate. An earlier study showed that ILD occurs independent of the free payload, and its incidence and severity depend on the dose and frequency of administration of the ADC.^[^ ^61^ ^]^ In addition, it is worth mentioning that Enhertu could achieve safer clinical use by developing new guidelines for toxicity management of ILD,^[^ ^62^ ^]^ providing potential solutions to remedy the occurrence and severity of this treatment‐related adverse event.

## 3 Conclusion

As a growing class of “magic bullets”, the heterogeneity of the “target” is a vital factor affecting the function of antibody–drug conjugates (ADCs). Accumulating preclinical and clinical evidence strongly demonstrates that the bystander effect is an essential player in the mechanism of action of ADCs. ADC treatment of heterogeneous tumors through the bystander‐killing effect tends to be more fact than fiction. However, the few amounts of comprehensive methods for rational identification of bystander payloads and ADCs limited the clinical potential of this strategy. Encouraged by this, we applied GAT to describe payloads’ bystander‐killing effect and obtained a series of exatecan derivatives as potential warheads through rational modification and screening according to our scoring model. Among them, Ed9, a novel payload with satisfactory permeability and bioactivity, was further used to construct ADC. In the early exploration, we found that the tetrapeptide linker, which performed well in T‐DXd, was incompatible with Ed9. Therefore, further linker replacement was performed to solve the problem of inefficient payload release caused by cyclopropyl substitution. To our surprise, the Val‐Ala dipeptide linker that avoided phenylalanine maintained the lower aggregation rate (Monomer > 98.5%) of the ADC using a more lipophilic payload, revealing the complexity of payload and linker optimization. Further, we constructed and systematically characterized a series of HER2‐targeting ADCs using commercially available trastuzumab and evaluated the antitumor activity of the optimal Tras‐VA‐Ed9 (T‐VEd9). In both in vivo and in vitro models of HER2 heterogeneity, T‐VEd9 exhibited killing effects on HER2‐negative cells (TGI = 99.3%), which we attributed to the novel payload's more potent bystander effect.

In summary, we constructed antibody‐drug conjugates with potent bystander‐killing effects through B‐K score‐directed rational workflow for the first time. These molecules have shown higher therapeutic potential without causing any systemic toxicity. Further preclinical research on T‐VEd9 is ongoing, and we expect to see its therapeutic potential in more complex patient‐driven and metastatic tumor models. It is worth mentioning that in subsequent attempts, we found that our score model has shown high accuracy on various types of warheads, including payloads with modification potential such as tubulysins. In any case, our study provides an approach to systematically study payload derivatives from a more accurate bystander‐killing description, which could suggest modifications for different types of toxins to enhance or attenuate bystander effects, thus holding promise as a novel approach to advance ADC therapy toward clinical translation.

## 4 Experimental Section

### Materials

Exatecan mesylate was purchased from Jiangsu Aikon (Nanjing, China). DXd and Mc‐GGFG‐DXd were purchased from Shanghai Biochempartner (Shanghai, China). E64 was purchased from Macklin (Shanghai, China). Tris(2‐carboxyethyl)phosphine (TCEP, analytical grade) was purchased from TCI Chemical (Shanghai, China). The rest of the chemical reagents for conventional synthesis were commercially available. Solvents for liquid chromatography and compound stock solution are analytical grade. Trastuzumab was a gift from Dr. Weijie Fang. T‐DM1 was purchased from MedChemExpress (Shanghai, China). Human liver cathepsin B was purchased from Sigma‐Aldrich (St Louis, MO, USA). Human liver S9 was purchased from CHI Scientific (Jiangyin, China). HRP Conjugated Goat Anti‐human IgG (HA1018) was purchased from HUABIO (Hangzhou, China). FITC‐labeled Goat Anti‐Human IgG(H+L) was purchased from Beyotime (Shanghai, China). Plasma was purchased from Kejing Biological Technology Co., Ltd. (Yancheng, China). Teal luciferase substrate (DTZ) was purchased from MeisenCTCC (Hangzhou, China). Cell counting kit‐8 was purchased from MedChemExpress (Shanghai, China). TMB Single‐Component Substrate solution was purchased from Solarbio (Beijing, China). Rapid Coomassie blue stain was purchased from Kejing Biological Technology Co., Ltd. (Yancheng, China). FuturePAGE Gel was purchased by ACE Biotechnology (Nanjing, China). Amicon Ultra Centrifugal Filters and PES Fliters were purchased from Sigma‐Aldrich (St Louis, MO, USA). Cell culture bottles and other related consumables were purchased from Thermo Fisher Scientific (MA, USA) or Corning (NY, USA).

### Instruments

Absorbance and fluorescence readings were performed with the SYNERGY H1 microplate reader (BioTek). PAGE was carried out using the Mini‐PROTEAN Tetra system (BioRad). Gel imaging was performed by the imaging system Azure 600 (azure biosystems). Protein and nucleic acid concentrations were determined using a Nanodrop spectrophotometer (Thermo Scientific). Mass spectra were collected using Agilent 1260/G6125B liquid chromatography single quadrupole mass spectrometer. NMR spectra were recorded at room temperature on the following spectrometers: Bruker AVANCE III spectrometer (500 MHz), JEOL (Akishima, Japan) JNM‐ECZ400S NMR spectrometer (400 MHz). High‐resolution mass spectrum (HRMS) was obtained on the following spectrometers: Agilent Technologies 6224 quadrupole/time LC/MS, Agilent 6540 quadrupole/time of flight mass spectrometry. High‐performance liquid chromatography (HPLC) for small molecules was performed on an Agilent 1260 Infinity II (LC03) instrument equipped with a C18 reversed‐phase column (Agilent Eclipse XDB‐C18, 4.6×250 mm, 5 µm) and a UV detector. The column temperature was 25°C, water (0.1% formic acid) was used as phase A, methanol was used as phase B, and the elution time was 30 min with a flow rate of 1.0 mL min^−1^. High‐performance liquid chromatography (HPLC) for antibodies and conjugates was performed on an Agilent 1260 Infinity II (LC03) instrument equipped with columns in specific methods below. MALDI‐TOF/TOF‐MSI was performed using a RapifleX MALDI‐TOF/TOF system (Bruker Daltonics, Germany). Flow cytometry was performed on the BD FACSCanto II (Becton Dickinson, USA). Fluorescent images were captured by Olympus Microscopy Fluorescence Imaging System (CKX53 equipped with pE‐300^lite^, Olympus). Living animal imaging was performed with the aid of an In vivo Imaging System (IVIS) Spectrum CT (PerkinElmer, USA).

### Cells and Mice

SKBR‐3 (MeisenCTCC), MDA‐MB‐453 (MeisenCTCC), MDA‐MB‐361 (MeisenCTCC), MDA‐MB‐231 (MeisenCTCC), and JIMT‐1 (MeisenCTCC) were cultured in Dulbecco's modified Eagle's medium (Gibco) supplemented with 10% FBS (Fetal Bovine Serum) (MeisenCTCC, CTCC‐002‐001) and penicillin–streptomycin (penicillin: 100 units mL^−1^; streptomycin: 100 µg mL^−1^) (MeisenCTCC, SJ000022). NCI‐N87 cells (MeisenCTCC) were cultured in RPMI1640 (Gibco) supplemented with 10% FBS (Fetal Bovine Serum) (MeisenCTCC, CTCC‐002‐001) and penicillin–streptomycin (penicillin: 100 units mL^−1^; streptomycin: 100 µg mL^−1^) (MeisenCTCC, SJ000022). The MDA‐MB‐231/GFP cells were constructed by Pricella (Procell Life Science&Technology Co., Ltd., Wuhan, China). The MDA‐MB‐231/teLuc cells were constructed by MeisenCTCC (Zhejiang Meisen Cell Technology Co., Ltd., Hangzhou, China). All cells were cultured at 37°C under 5% CO_2_. All cell lines were authenticated by STR profiling and routinely checked for mycoplasma contamination. All procedures were approved by the animal experiment center of the Institute of Basic Medicine and Cancer (IBMC), Hangzhou Institution of Medicine (HIM), Chinese Academy of Sciences, and were performed in accordance with the institutional guidelines for animal care and use (2023R0024). All animals were maintained under a 12 h light‐dark cycle and controlled conditions, namely 20–26°C and 40–70% relative humidity, with free access to water and food.

### Graph Attention Model

The molecule permeability dataset from the Excel file was preprocessed to remove duplicate SMILES entries and then the unique SMILES, alongside their corresponding properties, are converted into a list. Molecular graphs were generated using DeepChem's MolGraphConvFeaturizer, with nodes as atoms and edges as bonds. The featurized data was made into a DeepChem NumpyDataset and partitioned into train, validation, and test sets with an 8:1:1 ratio. The Normalization Transformer is further performed to standardize the target property values. Hyperparameters were optimized with Optuna, using the validation Pearson R2 score as the objective function. This process entails constructing a GAT model with hyperparameters sampled by Optuna, training the model for a predetermined number of epochs, and evaluating its performance on the validation dataset. The hyperparameters and their search spaces include learning rate with log‐uniform sampling between 1e‐5 and 1e‐2, weight decay with log‐uniform sampling between 1e‐6 and 1e‐3, number of epochs with integer sampling between 200 and 500, batch size with categorical sampling among, ^[^ ^32^, ^64^ ^, 128, 256]^ predictor hidden features with categorical sampling among, ^[^ ^32^, ^64^ ^, 128, 256]^ and predictor dropout with log‐uniform sampling between 0.1 and 0.6. In addition, the number and aggregation methods of GAT layers were manually tuned. The best configuration was three GAT layers with mean, mean, and flatten aggregations. Upon determining the best hyperparameters from the study, which were {“batch_size”: 32, “learning_rate”: 0.00014547080624370068, “n_epochs”: 416, “predictor_dropout”: 0.208576001608173, “predictor_hidden_feats”: 128, “weight_decay”: 3.655402125086186e‐05}, The best hyperparameters were used to retrain the GAT model and evaluate on the test set.

### Chemical Synthesis

Unless stated otherwise, all compounds were prepared by conventional chemical reactions using commercial reagents. The detailed synthesis procedure and spectrum characterization was outlined in the Supplementary Materials.

### Cell Viability Assay

Cells were seeded in a 96‐well plate at 1000–4000 cells per well for different cell lines. After overnight incubation, serially diluted samples (100 µL) were added to each well, and the plate was incubated at 37°C for 72 h (payloads) or 108 h (ADCs). After the old medium was replaced with 100 µL fresh medium, 10 µL of the Cell Counting Kit‐8 (CCK‐8) was added to each well, and the plate was incubated at 37°C for 2 h. After gently agitating the plate, the absorbance at 450 nm was recorded using a BioTek Synergy microplate reader. EC_50_ values were calculated using GraphPad Prism 9.3 software. All assays were performed in triplicate.

### Parallel Artificial Membrane Permeability Assay

PAMPA was performed by WuXi AppTec Co., Ltd. (Wuxi, China). In brief, 150 µL of 10.0 µm donor solutions (5% DMSO) were added to each well of the donor plate, whose PVDF membrane was precoated with 5 µL of 1% lecithin/dodecane mixture. Then, 300 µL of PBS was added to each well of the PTFE acceptor plate. After incubation for 4 h at room temperature with shaking at 300 rpm, the plate was removed from the incubator, and the solution was transferred from each acceptor and donor well and mixed with acetonitrile (containing internal standard) as samples. Acceptor samples and donor samples were all analyzed by LC‐MS/MS to determine permeability rates (Pe). All assays were performed in duplicates.

### Topo 1‐Mediated DNA Cleavage Assay

DNA cleavage assay was performed by Huawei Pharmaceutical Co., Ltd. (Jinan, China) with reference to previous literature about camptothecin derivatives.^[^ ^63^ ^]^

### Antibody–Linker Conjugation

A solution of trastuzumab [5 mg mL^−1^ in PBS (pH 7.4) containing 2 mm EDTA] was treated with 9 molar equivalents of TCEP and shaken (600 rpm) at 37 °C for 2 h. Then, 14 molar equivalents of maleimide linker‐payload were added to the entirely reduced antibody solution with a total DMSO concentration < 7.5% (v/v). The solution was incubated (600 rpm) for another 2 h at room temperature. Post‐reaction, the conjugates were purified and buffer‐exchanged with PBS (pH 7.4) using an Amicon Ultra 50K centrifugal filters device and were sterile‐filtered (0.22 µM PES filters).

### Human Cathepsin B or Human Liver S9‐Mediated Cleavage Assay

NAC‐LPs were prepared by co‐incubation of MC‐linker‐payloads and 5 molar equivalents of NAC under 37°C for 2 h. Next, 50 µL of NAC‐LPs (200 µm) and 100 µL CTSB/HuS9 (30 UN/20 mg mL^−1^) were added to 350 µL CTSB activity buffer (50 mM sodium acetate, 100 mm NaCl, 8 mm L‐cysteine, and 1 mM EDTA, pH 5.0).^[^ ^64^ ^]^ Aliquots (100 µL) were taken at subsequent time points (*t* = 0, 6, 12, and 24 h). Then, 400 µL methanol containing 50 µm E64 was added, and centrifugation was performed for 10 min for protein sedimentation. Released payloads were analyzed on HPLC and LC‐MS and quantified using the standard curve method. All assays were performed in duplicates.

### Chromatographic Characterization

Reversed‐phase high‐performance liquid chromatography (RP‐HPLC) for antibodies and conjugates was performed on an Agilent 1260 Infinity II (LC03) instrument equipped with a reversed‐phase column (Agilent PLRP‐S 1000 Å, 2.1×150 mm, 8 µm). The column temperature was 80 °C, water (0.1% trifluoroacetic acid) was used as phase A, acetonitrile (0.1% trifluoroacetic acid) was used as phase B, and the elution time was 35 min with a flow rate of 0.4 mL min^−1^. The gradient was 25% A to 50% A from 3 min to 28 min, 50% A to 95% A from 28 to 30 min, and then 95% A to 25% A from 30 to 32 min. UV detection was monitored at 280 nm. Hydrophobic interaction chromatography (HIC) for antibodies and conjugates was performed on an Agilent 1260 Infinity II (LC03) instrument equipped with a HIC column (TOSOH TSKgel Butyl‐NPR, 4.6×10 cm). The column temperature was 25 °C, 2 m (NH_4_)_2_SO_4_ (containing 25 mm Na_2_HPO_4_) was used as phase A, 25 mm Na_2_HPO_4_ (pH 7.01) was used as phase B, isopropanol (IPA) was used as phase C, and the elution time was 25 min with a flow rate of 0.5 mL min^−1^. The gradient was 75% A and 25% B to 85% B and 15% C from 0 min to 15 min, 85% B and 15% C to 75% A and 25% B from 15 min to 18 min, and then keep 75% A and 25% B to 25 min. UV detection was monitored at 280 nm. Size exclusion chromatography (SEC) was performed by Hangzhou JUST Biotherapeutics Co., Ltd. on an ACQUITY UPLC system (Waters).

### Mass Spectrometry

The sample solution (2 µL) was spotted on the MALDI target plate and mixed together with 1 µL matrix solution. After air‐drying, MALDI‐TOF‐MS analysis was performed using a RapifleX MALDI‐TOF/TOF system (Bruker Daltonics, Germany).

### SDS‐PAGE

ADCs in PBS (0.25 mg mL^−1^, 36 µL, pH = 7.4) were mixed with 5× SDS loading buffer (with or without DTT). After heating (99 °C, 5 min) and centrifugation (15 000 rpm, 10 min), 5 µL PageRuler Prestained protein ladder (Thermo Scientific) and 15 µL samples were then loaded onto ACE 12% FuturePAGE Gel and run in Bis‐Tris buffer at 160 V for 45 min. The image of the gel was taken by the imaging system Azure 600 (Ex: 355 nm, Em: 550 nm).

### Cell‐Based ELISA Assay

Cell‐based ELISA was performed by referring to previous literature.^[^ ^31^ ^]^ In brief, NCI‐N87 or MDA‐MB‐231 cells were seeded at the quantity of 1×10^4^ per well to the 96‐well clear plate. After paraformaldehyde fixation and blocking with 0.2% BSA in PBS, cells were incubated overnight at 4 °C with gradient concentrations of antibodies or conjugates. Then cells were incubated with goat anti‐human IgG‐HRP conjugate at room temperature for 1 h. After washing, TMB single‐component substrate solution was added and incubated in the dark. 2N‐H_2_SO_4_ was added for reaction termination after 30 min, and then the absorbance at 450 nm was recorded using a BioTek Synergy microplate reader. All assays were performed in triplicate.

### Plasma Stability Test

Each ADC, at a concentration of 1 mg mL^−1^ (20 µL in PBS), was dispensed into undiluted BALB/c murine or human plasma, resulting in a final concentration of 50 µg mL^−1^ and incubated at 37 °C. Subsequent aliquots of 40 µL were systematically extracted at successive intervals (precisely at 0, 1, 3, 6, 12, 24, 48, 108, and 144 h) and conserved at −80 °C pending utilization. Released payloads were analyzed on HPLC and LC‐MS, with the quantification derived through the standard curve method. All tests were performed in duplicates.

### Flow Cytometry

After trypsin digestion, cells were collected by centrifugation at 600×g 4°C, washed once with 1 mL pre‐cooled 1×PBS (pH 7.4), and incubated with 1 mL primary antibody (trastuzumab, 50 nM) for 30 min on ice. Then, after the supernatant was discarded by centrifugation, cells were washed twice with pre‐cooled 1×PBS (pH 7.4), and incubated with 500 µL FITC‐labeled Goat Anti‐Human IgG(H+L) (1:500 dilution in 1×PBS, Beyotime, China) for 30 min on ice. Finally, after the supernatant was discarded by centrifugation, cells were washed once and resuspended with pre‐cooled 1×PBS, and the flow cytometry was performed on the BD FACSCanto II (Becton Dickinson, USA).

### In Vitro Co‐Culture Model for Bystander‐Killing Evaluation

HER2‐positive cells (NCI‐N87 or SKBR‐3) and HER2‐negative cells (MDA‐MB‐231/GFP) were seeded in a clear bottom white 96‐well plate at 1000 cells per well and 2500 or 5000 cells per well (different ratios). After overnight incubation, a single concentration or a serially diluted solution of each ADC was added. Cell viability was evaluated after 120 h using the cell counting kit‐8 assay. The mixed cell viability was determined by CCK‐8 assay. The HER2‐negative cell viability was determined by detecting the GFP fluorescence intensity (Ex: 488 nm, Em: 507 nm) with a BioTek Synergy microplate reader. In the supernatant proliferation inhibition experiment, gradient concentrations of ADC were incubated with NCI‐N87 (4000 cells per well) for 48 h. Then the supernatant was incubated with MDA‐MB‐231 (3000 cells per well) for 72 h, and the cells viability were measured by CCK‐8 assay.

### HER2‐Positive Breast and Gastric Cancer Xenograft Model

A suspension of 5 × 10^6^ SKBR‐3 cells in 100 µL of 1: 1 PBS/Matrigel solution was injected subcutaneously into the armpit of female BALB/c nude mice aged 5–6 weeks (Day 0). On day 7, the tumors reached an average volume of ≈120 mm^3^, mice were randomized to each group, and each drug or PBS was given to the mice at a single dose of 10 mg kg^−1^ through injection via the tail vein, with a volume of 10 mL kg^−1^. The size of the tumor and body weight were monitored twice a week. A suspension of 5 × 10^6^ NCI‐N87 cells in 100 µL of PBS solution was injected subcutaneously into the flank of female BALB/c nude mice aged 5–6 weeks (Day 0). On day 7, the tumors reached an average volume of ≈110 mm^3^, mice were randomized to each group, and each ADC was given to the mice at a single dose of 3 or 1 mg kg^−1^ through injection via the tail vein, with a volume of 10 mL kg^−1^. The size of the tumor and body weight were monitored three times a week. A suspension of 5 × 10^6^ JIMT‐1 cells in 100 µL of 1: 1 PBS/Matrigel solution was injected subcutaneously into the flank of female BALB/c nude mice aged 4–5 weeks (Day 0). On day 11, the tumors reached an average volume of ≈100 mm^3^, mice were randomized to each group, and each drug or PBS was given to the mice at a single dose of 5 mg kg^−1^ through injection via the tail vein, with a volume of 10 mL kg^−1^. The size of the tumor and body weight were monitored three times a week. The tumor volume was estimated on the mathematical formula, which is 1/2 × length × width^2^. In circumstances wherein the calculated tumor volume exceeded the threshold of 1000 mm^3^, the tumor's diameter surpassed 20 mm, or when the mice showed obvious signs of severe distress, humane euthanasia was promptly carried out. After the sacrifice of mice, the tumors were isolated and collected for IHC analysis performed by Haoke Biotechnology Co., Ltd.

### In Vivo Xenograft Mouse Model of HER2‐Heterogeneous Cancer

A mix suspension of 2 × 10^6^ NCI‐N87 cells and 5 × 10^6^ MDA‐MB‐231/teLuc cells in 100 µL of 1: 1 PBS/Matrigel solution was injected subcutaneously into the flank of female BALB/c nude mice aged 5–6 weeks (Day 0). On day 7, the tumors reached an average volume of ≈180 mm^3^, mice were randomized to each group, and each ADC was given to the mice at a single dose of 5 or 2 mg kg^−1^ through injection via the tail vein, with a volume of 10 mL kg^−1^. The size of the tumor and body weight were monitored three times a week. The tumor volume was estimated on the mathematical formula, which is 1/2 × length × width^2^. In circumstances wherein the calculated tumor volume exceeded the threshold of 1000 mm^3^, the tumor's diameter surpassed 20 mm, or when the mice showed obvious signs of severe distress, humane euthanasia was promptly carried out. After the sacrifice of mice, the tumors and organs were isolated and collected for IHC analysis and H&E staining performed by Haoke Biotechnology Co., Ltd.

### In Vivo Teal Luciferase Imaging

Teal luciferase activity of each mouse in the HER2‐heterogeneous xenograft model was determined by an in vivo Imaging System (IVIS) Spectrum CT (PerkinElmer, USA) once a week (Day 7, 14, 21, 35) 10 min after intraperitoneal injection of pre‐dissolved DTZ (MeisenCTCC). The amount of luminescence was analyzed using analysis software (Living Image Software version 4.3.1; PerkinElmer) as average radiance (p per s per cm^2^ per sr).

### Blood Chemistry Analysis

A volume of ≈500 µL of whole blood was collected from each mouse and allowed to clot for 60 min. Subsequently, the sample was centrifuged at 2000 × g for 20 min at room temperature. The resulting serum samples (200–300 µL) were stored at −80 °C until use. Further analysis was performed by Haoke Biotechnology Co., Ltd.

### Statistical Analysis

Statistical analysis was done by Excel and GraphPad Prism 9.3. Comparisons were made using a two‐tailed, unpaired Student's *t*‐test. *P* < 0.05 was considered statistically significant and all the results are expressed as a mean ± S.D. unless otherwise mentioned.

## Conflict of Interest

The authors declare no conflict of interest.

## Author Contributions

Y.G. and Z.S. contributed equally to this work. Y.G., Z.Z., L.F., J.C., and X.D. conceived the key concepts. Z.S. built the related prediction model. Y.G. and Y.S. performed the synthesis and structure elucidation of linkers and payloads. M.W. performed high‐resolution mass spectrometry analysis. Y.G. and J.S. performed MALDI‐TOF‐MS analysis. Y.G. conducted conjugation and characterization experiments of ADCs. Y.G., W.Z., Y.Q., J.S., and W.Z. carried out the in vitro evaluation and analysis. Y.G., W.Z., Q.S., J.L., and Y.L. participated in cell‐driven xenograft studies and analysis. L.S. and Z.S. conducted computational studies. Y.G. and J.C. analyzed the data and prepared the paper.

## Supporting information

Supporting Information

## Data Availability

The data that support the findings of this study are available from the corresponding author upon reasonable request.

## References

[1] Z. Fu , S. Li , S. Han , C. Shi , Y. Zhang , Sig Transduct Target Ther 2022, 7, 93. 10.1038/s41392-022-00947-7 PMC8941077 35318309

[2] C. H. Chau , P. S. Steeg , W. D. Figg , Lancet 2019, 394, 793. 31478503 10.1016/S0140-6736(19)31774-X

[3] J. D. Bargh , A. Isidro‐Llobet , J. S. Parker , D. R. Spring , Chem. Soc. Rev. 2019, 48, 4361. 31294429 10.1039/c8cs00676h

[4] A. Samantasinghar , N. P. Sunildutt , F. Ahmed , A. M. Soomro , A. R. C. Salih , P. Parihar , F. H. Memon , K. H. Kim , I. S. Kang , K. H. Choi , Biomed. Pharmacother. 2023, 161, 114408. 36841027 10.1016/j.biopha.2023.114408

[5] P. Guo , J. Huang , B. Zhu , A. C. Huang , L. Jiang , J. Fang , M. A. Moses , Sci. Adv. 2023, 9, eabq7866. 37146146 10.1126/sciadv.abq7866 PMC10162665

[6] F. Tang , L.‐X. Wang , W. Huang , Nat. Protoc. 2017, 12, 1702. 28749929 10.1038/nprot.2017.058 PMC5705183

[7] Y. Zeng , W. Shi , Q. Dong , W. Li , J. Zhang , X. Ren , C. Tang , B. Liu , Y. Song , Y. Wu , X. Diao , H. Zhou , H. Huang , F. Tang , W. Huang , Angew Chem Int Ed Engl 2022, 61, e202204132. 35737596 10.1002/anie.202204132

[8] J. Z. Drago , S. Modi , S. Chandarlapaty , Nat. Rev. Clin. Oncol. 2021, 18, 327. 33558752 10.1038/s41571-021-00470-8 PMC8287784

[9] P. Tarantino , R. Carmagnani Pestana , C. Corti , S. Modi , A. Bardia , S. M. Tolaney , J. Cortes , J.‐C. Soria , G. Curigliano , CA Cancer J Clin 2022, 72, 165. 34767258 10.3322/caac.21705

[10] C. Dumontet , J. M. Reichert , P. D. Senter , J. M. Lambert , A. Beck , Nat Rev Drug Discov 2023, 22, 641. 37308581 10.1038/s41573-023-00709-2

[11] R. A. Burrell , N. McGranahan , J. Bartek , C. Swanton , Nature 2013, 501, 338. 24048066 10.1038/nature12625

[12] D. Zardavas , A. Irrthum , C. Swanton , M. Piccart , Nat. Rev. Clin. Oncol. 2015, 12, 381. 25895611 10.1038/nrclinonc.2015.73

[13] K. Polyak , J. Clin. Invest. 2011, 121, 3786. 21965334 10.1172/JCI60534 PMC3195489

[14] S. García‐Alonso , A. Ocaña , A. Pandiella , Cancer Res. 2018, 78, 2159. 29653942 10.1158/0008-5472.CAN-17-3671

[15] A. Ocaña , E. Amir , A. Pandiella , Breast Cancer Res. 2020, 22, 15. 32005279 10.1186/s13058-020-1252-7 PMC6995165

[16] S. Loibl , L. Gianni , Lancet 2017, 389, 2415. 27939064 10.1016/S0140-6736(16)32417-5

[17] C. E. DeSantis , J. Ma , M. M. Gaudet , L. A. Newman , K. D. Miller , A. Goding Sauer , A. Jemal , R. L. Siegel , CA Cancer J Clin 2019, 69, 438. 31577379 10.3322/caac.21583

[18] G. M. Choong , G. D. Cullen , C. C. O'Sullivan , CA Cancer J Clin 2020, 70, 355. 32813307 10.3322/caac.21634

[19] Y. Ishimine , A. Goto , Y. Watanabe , H. Yajima , S. Nakagaki , T. Yabana , T. Adachi , Y. Kondo , K. Kasai , Case Reports in Gastrointestinal Medicine 2015, 2015, 132030. 25893119 10.1155/2015/132030 PMC4393931

[20] N. Harbeck , M. Gnant , Lancet 2017, 389, 1134. 27865536 10.1016/S0140-6736(16)31891-8

[21] H. Seol , H. J. Lee , Y. Choi , H. E. Lee , Y. J. Kim , J. H. Kim , E. Kang , S.‐W. Kim , S. Y. Park , Mod Pathol 2012, 25, 938. 22388760 10.1038/modpathol.2012.36

[22] J. Ju , F. Du , S.‐L. Gao , Y.‐R. Si , N.‐L. Hu , D.‐X. Liu , X. Wang , J. Yue , F.‐C. Zheng , Y.‐K. Kang , Z.‐X. Yang , F. Ma , B.‐H. Xu , P. Yuan , Breast Cancer Res. Treat. 2022, 194, 221. 35699854 10.1007/s10549-022-06629-w

[23] F. Schettini , A. Prat , The Breast 2021, 59, 339. 34392185 10.1016/j.breast.2021.07.019 PMC8374722

[24] J. Cortés , S.‐B. Kim , W.‐P. Chung , S.‐A. Im , Y. H. Park , R. Hegg , M. H. Kim , L.‐M. Tseng , V. Petry , C.‐F. Chung , H. Iwata , E. Hamilton , G. Curigliano , B. Xu , C.‐S. Huang , J. H. Kim , J. W. Y. Chiu , J. L. Pedrini , C. Lee , Y. Liu , J. Cathcart , E. Bako , S. Verma , S. A. Hurvitz , N. Engl. J. Med. 2022, 386, 1143. 35320644 10.1056/NEJMoa2115022

[25] S. Modi , W. Jacot , T. Yamashita , J. Sohn , M. Vidal , E. Tokunaga , J. Tsurutani , N. T. Ueno , A. Prat , Y. S. Chae , K. S. Lee , N. Niikura , Y. H. Park , B. Xu , X. Wang , M. Gil‐Gil , W. Li , J.‐Y. Pierga , S.‐A. Im , H. C. F. Moore , H. S. Rugo , R. Yerushalmi , F. Zagouri , A. Gombos , S.‐B. Kim , Q. Liu , T. Luo , C. Saura , P. Schmid , T. Sun , et al., N. Engl. J. Med. 2022, 387, 9.35665782

[26] Cancer Discov. 2022, 12, 1828.

[27] Y. Ogitani , K. Hagihara , M. Oitate , H. Naito , T. Agatsuma , Cancer Sci. 2016, 107, 1039. 27166974 10.1111/cas.12966 PMC4946713

[28] Y. Ogitani , T. Aida , K. Hagihara , J. Yamaguchi , C. Ishii , N. Harada , M. Soma , H. Okamoto , M. Oitate , S. Arakawa , T. Hirai , R. Atsumi , T. Nakada , I. Hayakawa , Y. Abe , T. Agatsuma , Clin. Cancer Res. 2016, 22, 5097. 27026201 10.1158/1078-0432.CCR-15-2822

[29] H. Xu , X. Sheng , L. Zhou , X. Yan , S. Li , Z. Chi , C. Cui , L. Si , B. Tang , L. Mao , B. Lian , X. Wang , X. Bai , J. Li , J. Guo , JCO 2022, 40, 4519.

[30] F. Giugliano , C. Corti , P. Tarantino , F. Michelini , G. Curigliano , Curr. Oncol. Rep. 2022, 24, 809. 35305211 10.1007/s11912-022-01266-4

[31] C. M. Yamazaki , A. Yamaguchi , Y. Anami , W. Xiong , Y. Otani , J. Lee , N. T. Ueno , N. Zhang , Z. An , K. Tsuchikama , Nat. Commun. 2021, 12, 3528. 34112795 10.1038/s41467-021-23793-7 PMC8192907

[32] Z. Jiang , T. Sun , X. Wang , Q. Liu , M. Yan , Z. Tong , C. Geng , J. Tang , Y. Yin , G. Yu , J. Wang , W. Su , S. Wang , Y. Pan , H. Yang , JCO 2022, 40, 1102.

[33] X. Hu , J. Zhang , R. Liu , S. Gao , J. Wu , Y. Wang , Y. Hao , J. Ge , Y. Qing , S. Yi , Q. Yang , H. Rao , J. Yuan , JCO 2022, 40, 1037.

[34] L. R. Staben , J. Chen , J. D. Cruz‐Chuh , G. Del Rosario , M. A. Go , J. Guo , S. C. Khojasteh , K. R. Kozak , G. Li , C. Ng , G. D. Lewis Phillips , T. H. Pillow , R. K. Rowntree , J. Wai , B. Wei , K. Xu , Z. Xu , S.‐F. Yu , D. Zhang , P. S. Dragovich , J. Med. Chem. 2020, 63, 9603. 32787101 10.1021/acs.jmedchem.0c00691

[35] L. Shen , X. Sun , Z. Chen , Y. Guo , Z. Shen , Y. Song , W. Xin , H. Ding , X. Ma , W. Xu , W. Zhou , J. Che , L. Tan , L. Chen , S. Chen , X. Dong , L. Fang , F. Zhu , Nucleic Acids Res. 2024, 52, D1097. 37831118 10.1093/nar/gkad831 PMC10768060

[36] G. Xiong , Z. Wu , J. Yi , L. Fu , Z. Yang , C. Hsieh , M. Yin , X. Zeng , C. Wu , A. Lu , X. Chen , T. Hou , D. Cao , Nucleic Acids Res. 2021, 49, W5. 33893803 10.1093/nar/gkab255 PMC8262709

[37] J. Morstein , A. Capecchi , K. Hinnah , B. Park , J. Petit‐Jacques , R. C. Van Lehn , J.‐L. Reymond , D. Trauner , J. Am. Chem. Soc. 2022, 144, 18532. 36178375 10.1021/jacs.2c07833

[38] N.‐N. Wang , J. Dong , Y.‐H. Deng , M.‐F. Zhu , M. Wen , Z.‐J. Yao , A.‐P. Lu , J.‐B. Wang , D.‐S. Cao , J. Chem. Inf. Model. 2016, 56, 763. 27018227 10.1021/acs.jcim.5b00642

[39] S. Skolnik , X. Lin , J. Wang , X.‐H. Chen , T. He , B. Zhang , J. Pharm. Sci. 2010, 99, 3246. 20166204 10.1002/jps.22080

[40] J. Wang , L. Bell , in ADME‐Enabling Technologies in Drug Design and Development (Eds.: D. Zhang , S. Surapaneni ), Wiley, New York 2012, pp. 129–159.

[41] T. Oguma , M. Yamada , T. Konno , K. Inukai , M. Nakaoka , Biol. Pharm. Bull. 2001, 24, 176. 11217088 10.1248/bpb.24.176

[42] H. Okamoto , M. Oitate , K. Hagihara , H. Shiozawa , Y. Furuta , Y. Ogitani , H. Kuga , Xenobiotica 2020, 50, 1242. 32306807 10.1080/00498254.2020.1755909

[43] B. Gikanga , N. S. Adeniji , T. W. Patapoff , H.‐W. Chih , L. Yi , Bioconjugate Chem. 2016, 27, 1040. 10.1021/acs.bioconjchem.6b00055 26914498

[44] Z. Su , D. Xiao , F. Xie , L. Liu , Y. Wang , S. Fan , X. Zhou , S. Li , Acta Pharm. Sin. B 2021, 11, 3889. 35024314 10.1016/j.apsb.2021.03.042 PMC8727783

[45] W. Weng , T. Meng , Q. Zhao , Y. Shen , G. Fu , J. Shi , Y. Zhang , Z. Wang , M. Wang , R. Pan , L. Ma , C. Chen , L. Wang , B. Zhou , H. Zhang , J. Pu , J. Zhang , Y. P. Hu , G. Hua , Y. Qian , S.‐H. Liu , W. Hu , X. Meng , Cancer Discov 2023, 13, 950. 36693125 10.1158/2159-8290.CD-22-1368

[46] W. Lai , S. Zhao , Q. Lai , W. Zhou , M. Wu , X. Jiang , X. Wang , Y. Peng , X. Wei , L. Ouyang , L. Gou , H. Chen , Y. Wang , J. Yang , J. Med. Chem. 2022, 65, 11679. 35982539 10.1021/acs.jmedchem.2c00471

[47] A. J. Bessire , T. E. Ballard , M. Charati , J. Cohen , M. Green , M.‐H. Lam , F. Loganzo , B. Nolting , B. Pierce , S. Puthenveetil , L. Roberts , K. Schildknegt , C. Subramanyam , Bioconjugate Chem. 2016, 27, 1645. 10.1021/acs.bioconjchem.6b00192 27206324

[48] A. J. Bessire , C. Subramanyam , in Antibody‐Drug Conjugates: Methods and Protocols (Ed.: L. N. Tumey ), Springer US, New York, NY, 2020, pp. 341–351.

[49] D. V. Nadkarni , in Antibody‐Drug Conjugates: Methods and Protocols (Ed.: L. N. Tumey ), Springer US, New York, NY, 2020, pp. 37–49.

[50] N. G. Caculitan , J. dela C Chuh , Y. Ma , D. Zhang , K. R. Kozak , Y. Liu , T. H. Pillow , J. Sadowsky , T. K. Cheung , Q. Phung , B. Haley , B.‐C. Lee , R. W. Akita , M. X. Sliwkowski , A. G. Polson , Cancer Res. 2017, 77, 7027. 29046337 10.1158/0008-5472.CAN-17-2391

[51] Y. Anami , C. M. Yamazaki , W. Xiong , X. Gui , N. Zhang , Z. An , K. Tsuchikama , Nat. Commun. 2018, 9, 2512. 29955061 10.1038/s41467-018-04982-3 PMC6023893

[52] S. Wu , D. K. Shah , in Antibody‐Drug Conjugates: Methods and Protocols (Ed.: L. N. Tumey ), Springer US, New York, NY, 2020, pp. 329–340.

[53] L. Conilh , G. Fournet , E. Fourmaux , A. Murcia , E.‐L. Matera , B. Joseph , C. Dumontet , W. Viricel , Pharmaceuticals 2021, 14, 247. 33803327 10.3390/ph14030247 PMC8000490

[54] E. Díaz‐Rodríguez , L. Gandullo‐Sánchez , A. Ocaña , A. Pandiella , Cancers 2022, 14, 154. 10.3390/cancers14010154 PMC8750930 35008318

[55] X. Sun , Y. Zhang , H. Li , Y. Zhou , S. Shi , Z. Chen , X. He , H. Zhang , F. Li , J. Yin , M. Mou , Y. Wang , Y. Qiu , F. Zhu , Nucleic Acids Res. 2023, 51, D1263. 36243960 10.1093/nar/gkac812 PMC9825618

[56] M. Barok , J. Isola , Z. Pályi‐Krekk , P. Nagy , I. Juhász , G. Vereb , P. Kauraniemi , A. Kapanen , M. Tanner , G. Vereb , J. Szöllösi , Mol. Cancer Ther. 2007, 6, 2065. 17620435 10.1158/1535-7163.MCT-06-0766

[57] K. Köninki , M. Barok , M. Tanner , S. Staff , J. Pitkänen , P. Hemmilä , J. Ilvesaro , J. Isola , Cancer Lett 2010, 294, 211. 20193978 10.1016/j.canlet.2010.02.002

[58] H.‐W. Yeh , O. Karmach , A. Ji , D. Carter , M. M. Martins‐Green , H. Ai , Nat. Methods 2017, 14, 971. 28869756 10.1038/nmeth.4400 PMC5678970

[59] P. Conte , P. A. Ascierto , G. Patelli , R. Danesi , A. Vanzulli , F. Sandomenico , P. Tarsia , A. Cattelan , A. Comes , M. De Laurentiis , A. Falcone , D. Regge , L. Richeldi , S. Siena , ESMO Open 2022, 7, 100404. 35219244 10.1016/j.esmoop.2022.100404 PMC8881716

[60] P. Tarantino , S. Modi , S. M. Tolaney , J. Cortés , E. P. Hamilton , S.‐B. Kim , M. Toi , F. Andrè , G. Curigliano , JAMA Oncol 2021, 7, 1873. 34647966 10.1001/jamaoncol.2021.3595

[61] K. Kumagai , T. Aida , Y. Tsuchiya , Y. Kishino , K. Kai , K. Mori , Cancer Sci. 2020, 111, 4636. 33051938 10.1111/cas.14686 PMC7734153

[62] S. M. Swain , M. Nishino , L. H. Lancaster , B. T. Li , A. G. Nicholson , B. J. Bartholmai , J. Naidoo , E. Schumacher‐Wulf , K. Shitara , J. Tsurutani , P. Conte , T. Kato , F. Andre , C. A. Powell , Cancer Treat. Rev. 2022, 106, 102378. 35430509 10.1016/j.ctrv.2022.102378

[63] Z. Miao , L. Zhu , G. Dong , C. Zhuang , Y. Wu , S. Wang , Z. Guo , Y. Liu , S. Wu , S. Zhu , K. Fang , J. Yao , J. Li , C. Sheng , W. Zhang , J. Med. Chem. 2013, 56, 7902. 24069881 10.1021/jm400906z

[64] D. Xiao , L. Zhao , F. Xie , S. Fan , L. Liu , W. Li , R. Cao , S. Li , W. Zhong , X. Zhou , Theranostics 2021, 11, 2550. 33456559 10.7150/thno.51232 PMC7806464

