# Supplementary material for: Rational Identification of Novel Antibody‐Drug Conjugate with High Bystander Killing Effect against Heterogeneous Tumors
Source: Adv Sci (Weinh). 2024 Jan 25;11(13):2306309. doi: 10.1002/advs.202306309 (PMC10987111; doi:10.1002/advs.202306309)
Supplement: Supplementary file 1 — Supporting Information [file ADVS-11-2306309-s001.pdf]

ADVANCED SCIENCE
Open Access

Supporting Information

for *Adv. Sci.*, DOI 10.1002/advs.202306309

Rational Identification of Novel Antibody-Drug Conjugate with High Bystander Killing Effect against Heterogeneous Tumors

Yu Guo, Zheyuan Shen, Wenbin Zhao, Jialiang Lu, Yi Song, Liteng Shen, Yang Lu, Mingfei Wu, Qiuqiu Shi, Weihao Zhuang, Yueping Qiu, Jianpeng Sheng, Zhan Zhou, Luo Fang, Jinxin Che and Xiaowu Dong**

Supporting Information

Rational Identification of Novel Antibody-Drug Conjugate with High Bystander Killing Effect against Heterogeneous Tumors

Yu Guo, Zheyuan Shen, Wenbin Zhao, Jialiang Lu, Yi Song, Liteng Shen, Yang Lu, Mingfei Wu, Qiuqiu Shi, Weihao Zhuang, Yueping Qiu, Jianpeng Sheng, Zhan Zhou, Luo Fang, Jinxin Che, and Xiaowu Dong**

This PDF file includes:

Figs. S1 to S15
Tables S1 to S5
Synthesis
Supplementary References

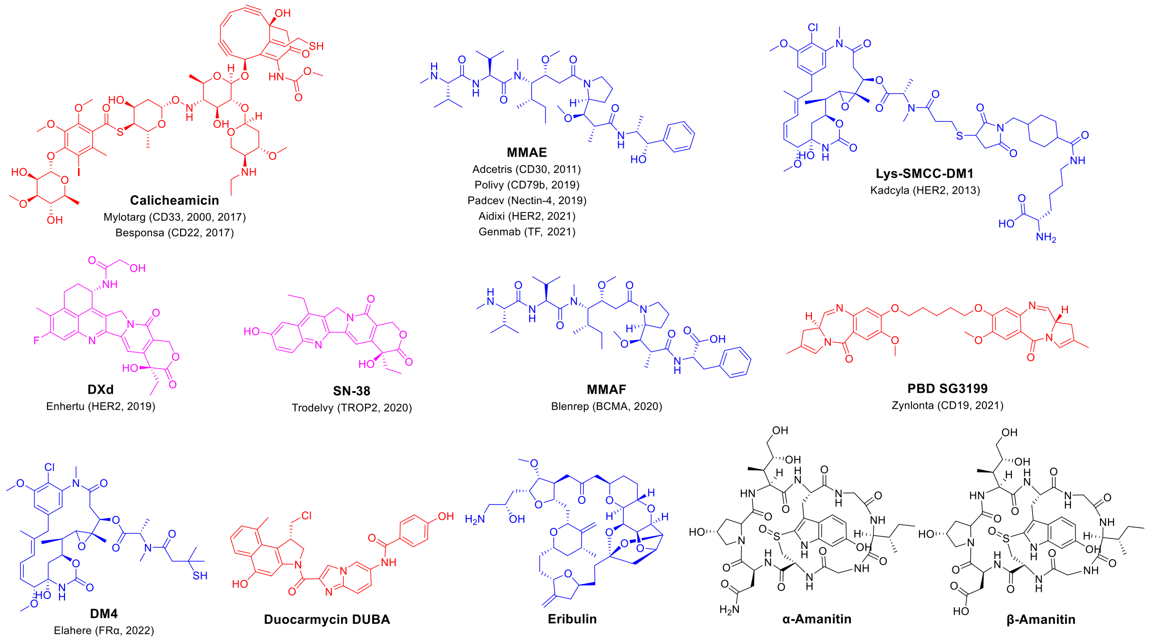

Figure S1. Structure of ADC payloads currently in clinic or development. (Red) DNA damaging agents; (Blue) microtubule inhibitors; (Pink) topoisomerase I inhibitors; (Black) RNA polymerase II inhibitors.

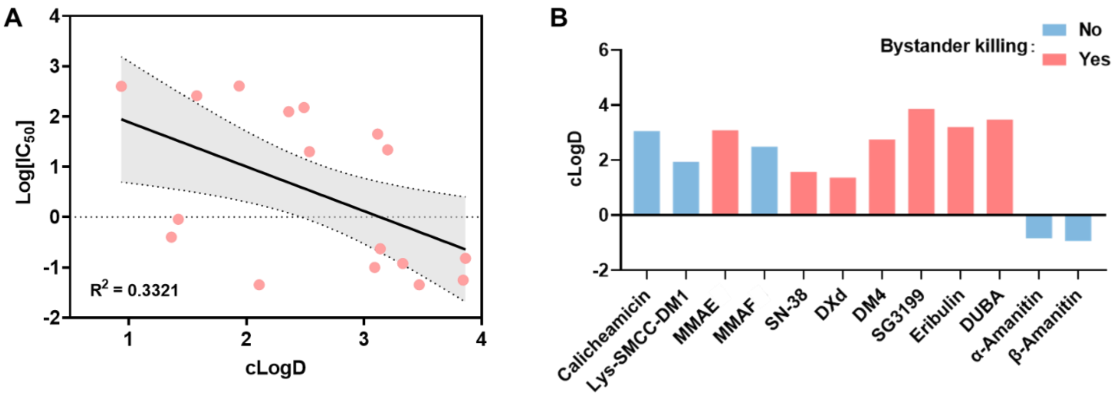

Figure S2. (A) Correlation analysis of payload cytotoxicity and cLogD. The red dots represent the reported payload. The gray-shaded region represents the 90% confidence interval for linear regression fit. Linear fitting was performed by GraphPad Prism 9.3 software. (B) cLogD and bystander killing effect of payloads currently in clinic or development.

A

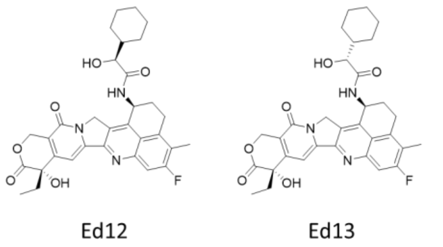

B

Compound	B Score	cLogD	K Score	B.K. Score	pIC_{50}
**DXd**	2.143	1.362	-0.246	1.992	1.131
**Ed12**	2.172	2.99	-0.949	1.223	0.901
**Ed13**	2.172	2.99	-0.949	1.223	0.946

Figure S3. (A) Structure of two exatecan derivatives (Ed12 and Ed13) not in the generated data set. (B) cLogD, B-K score and pIC50 of Ed12 and Ed13, pIC50 normalized from IC50 (μM) against SKBR-3.

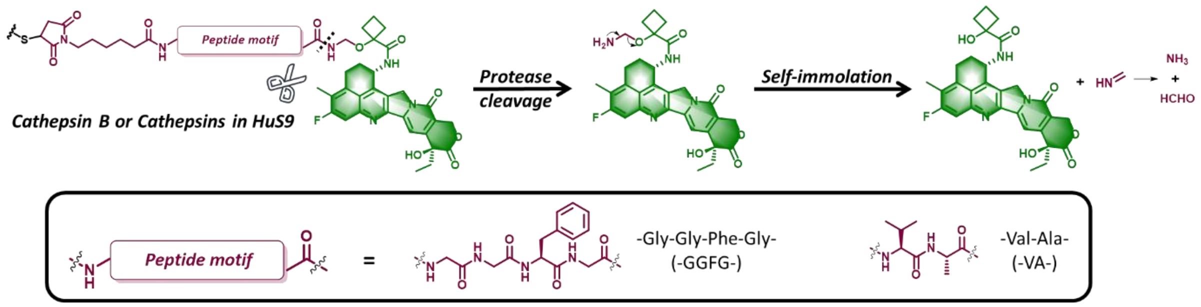

Figure S4. The mechanism and processes of GGFG and VA linker cleavage under cathepsin B and HuS9 treatment.

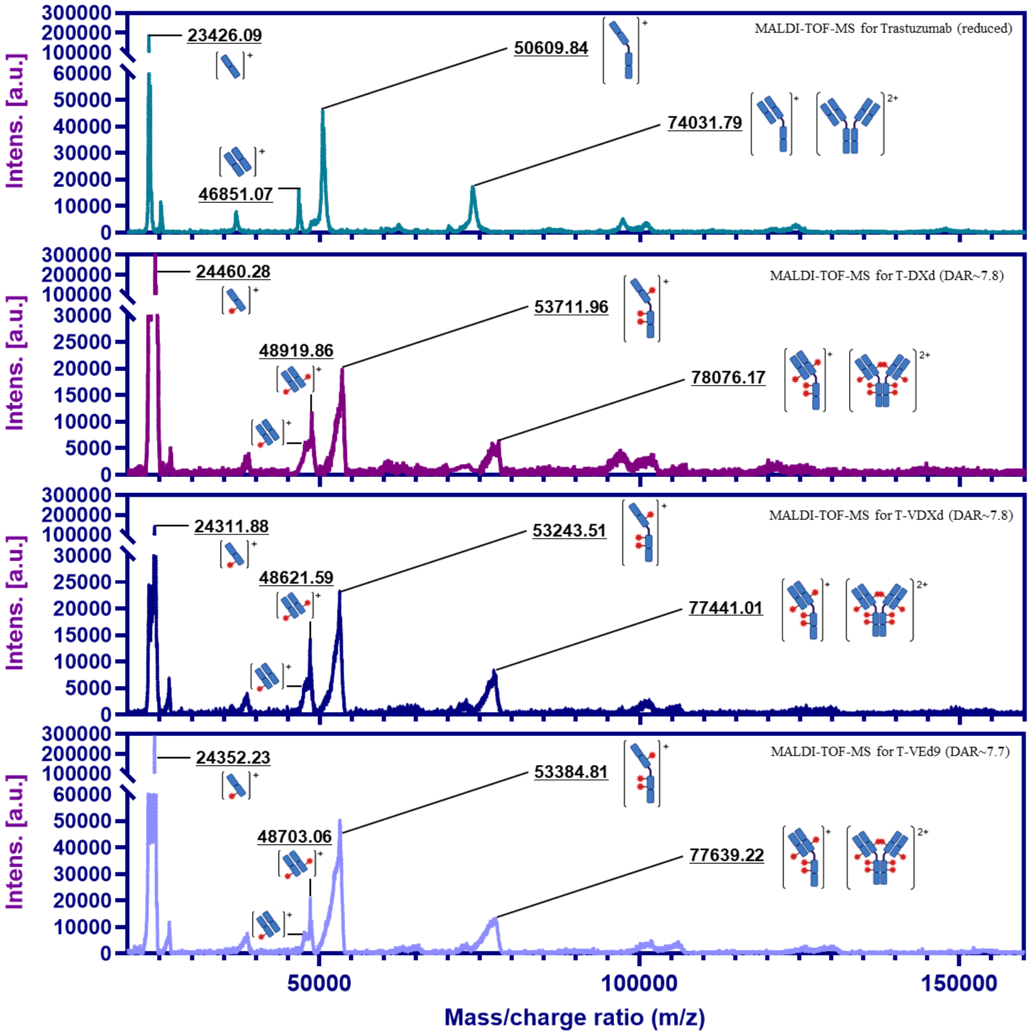

Figure S5. MALDI-TOF-MS analysis of Trastuzumab, T-DXd, T-VDXd, and T-VEd9.

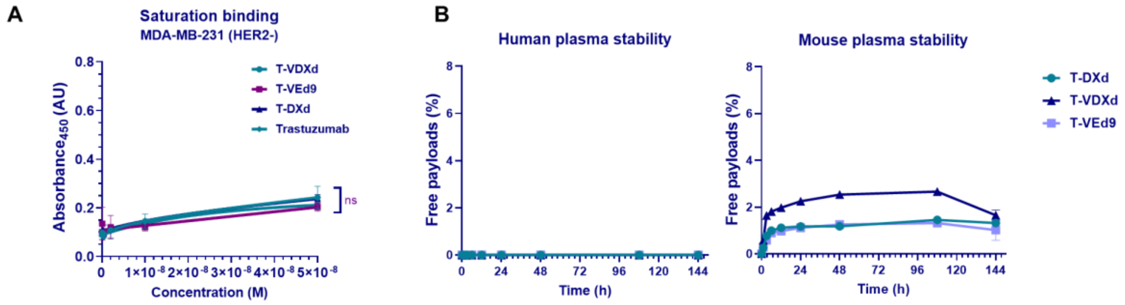

Figure S6. Characterization of novel trastuzumab-Eds conjugates. (A) Saturation-binding curves obtained by MDA-MB-231 (HER2-) cell-based ELISA. (B) Ex-vivo human and balb/c nude mouse plasma stability assay. HPLC and LC/ESI-MS monitored the payload release. All data shown are representative of more than two independent duplicates. Error bars represent S.D. Curve fitting and unpaired t-tests were performed using GraphPad Prism 9.3 software.

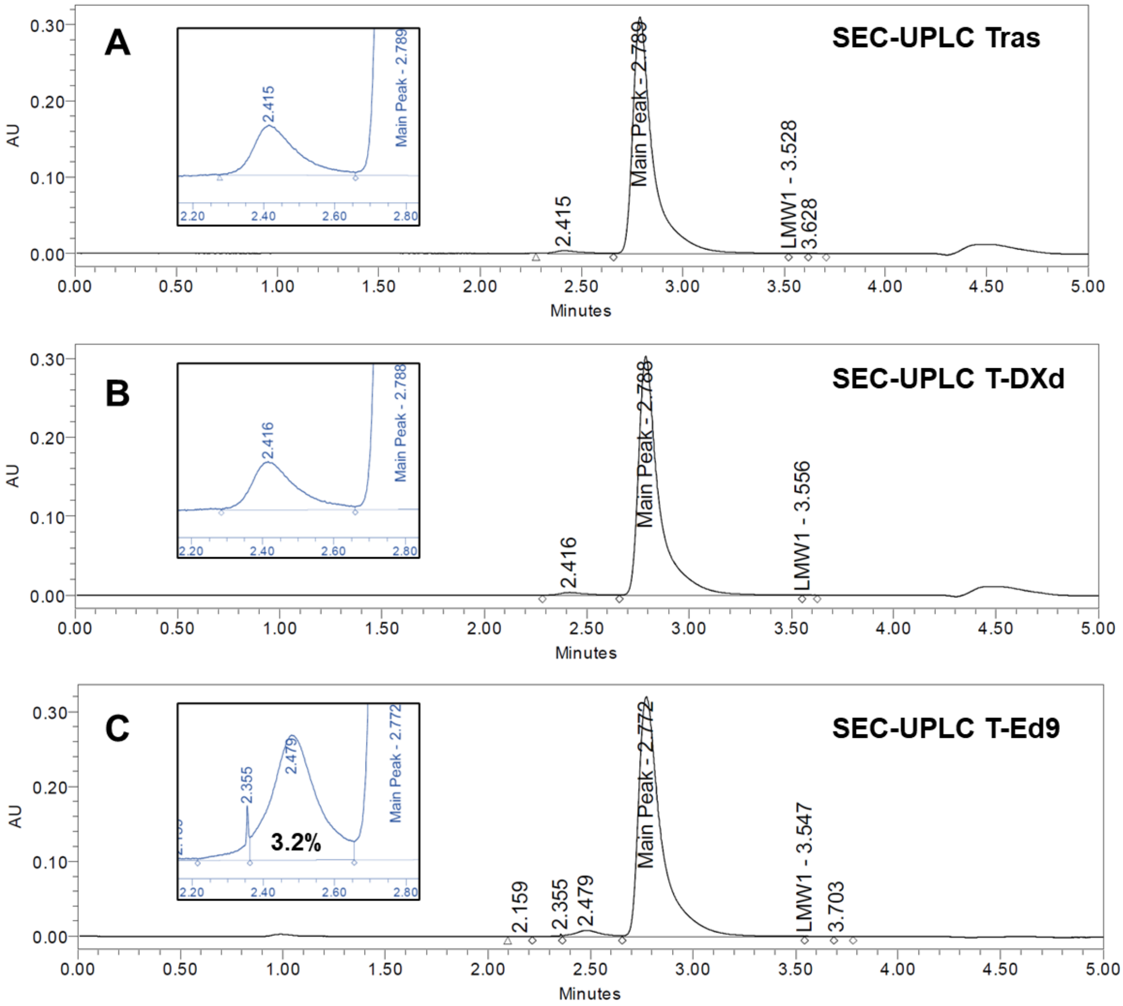

Figure S7. Size-exclusion chromatography (SEC) analysis of DAR8 ADCs. The absorbance wavelength was 280 nm. The isomer ratio was calculated by absorbance area.

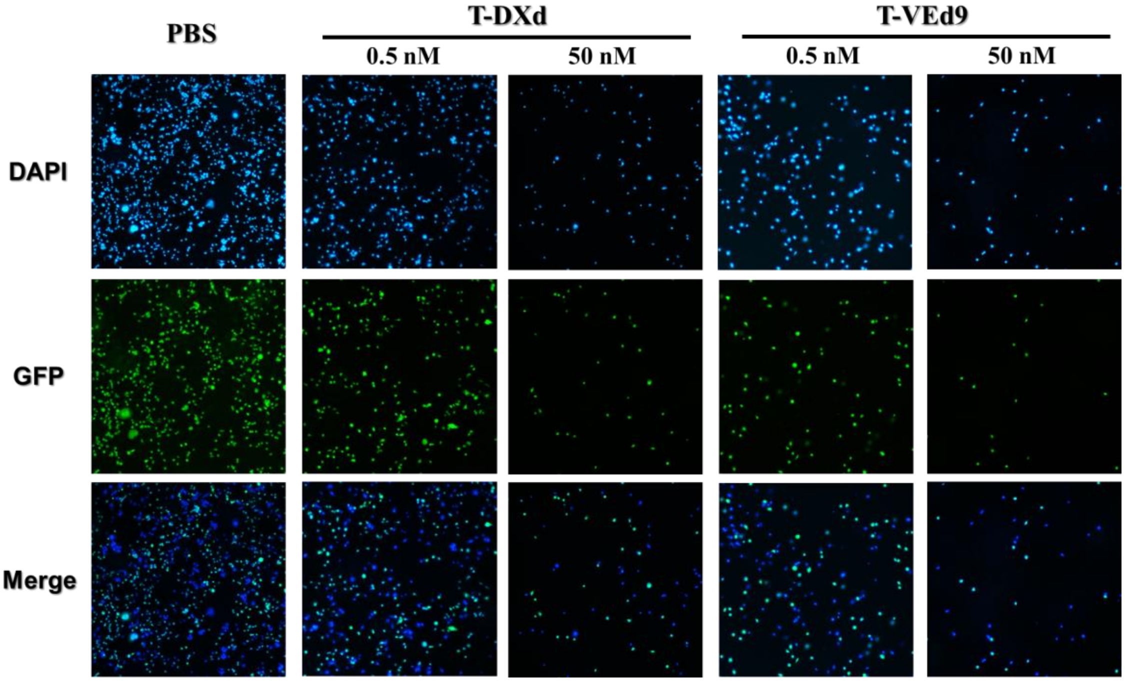

Figure S8. Fluorescence imaging of SKBR-3 and MDA-MB-231/GFP (Green) co-culture system with the treatment of different concentration ADCs. the nuclei were stained with DAPI (Blue). Scale bar: 25 μm.

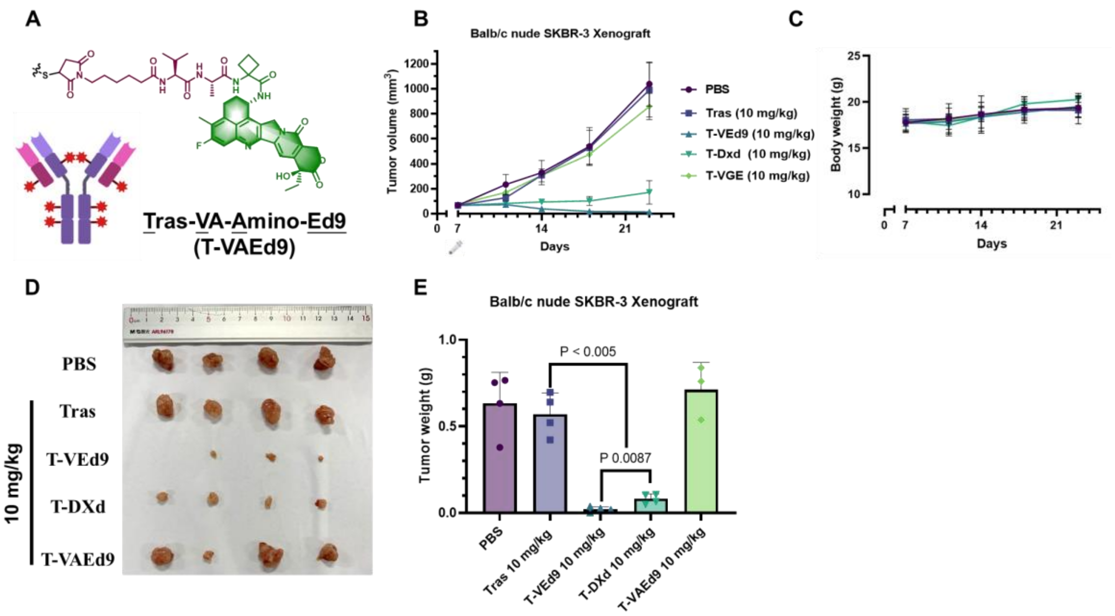

Figure S9. Anti-tumor activity in HER2+ SKBR-3 breast cancer model. (A) Structure of Tras-VA-Amino-Ed9 (T-VAEd9). (B-C) Tumor volume and body weight change in HER2+ SKBR-3 breast cancer model following a single intravenous ADC dose of 10 mg/kg. (D) The tumor tissues collected at the treatment endpoint of PBS control and each ADC. (E) Tumor weight. Error bars represent S.D. Unpaired t-tests were performed by GraphPad Prism 9.3 software.

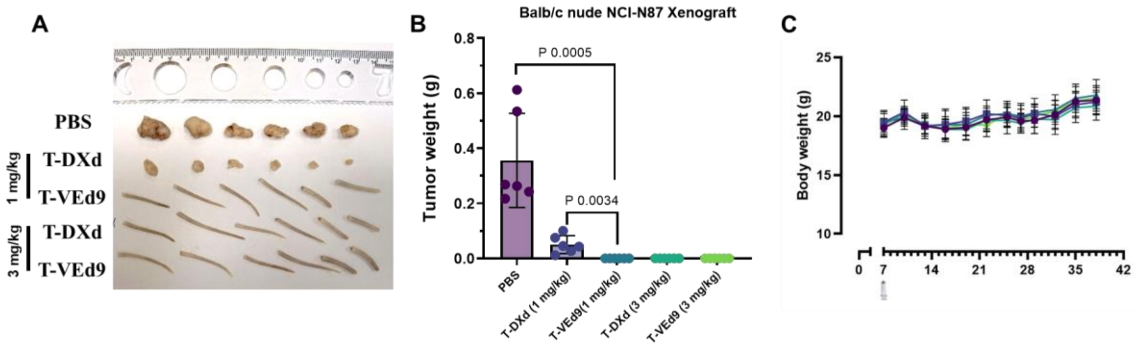

Figure S10. Anti-tumor activity in HER2+ NCI-N87 gastric cancer model. (A) The tumor tissues collected at the treatment endpoint of PBS control and each ADC. (B) Tumor weight. (C) Body weight change in HER2+ NCI-N87 gastric cancer model following a single intravenous ADC dose of 1 or 3 mg/kg. Error bars represent S.D. Unpaired t-tests were performed by GraphPad Prism 9.3 software.

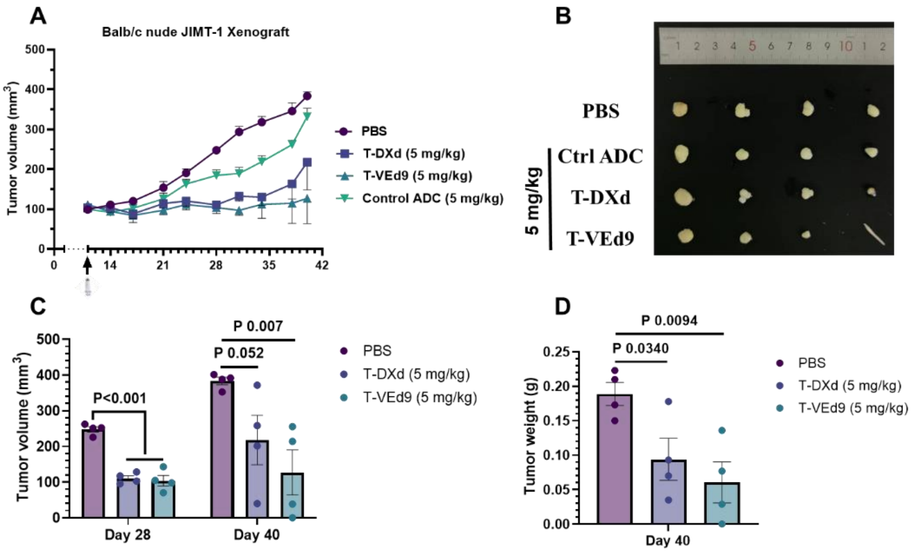

Figure S11. Anti-tumor activity in JIMT-1 xenograft model. (A) Tumor volume change following a single intravenous ADC dose of 5 mg/kg. Control ADC with an unstable linker. (B) The tumor tissues collected at the Day 40 of PBS control and each ADC. (C) Tumor volume analysis at Day 28 and 40. (D) Tumor weight. Error bars represent S.E.M. Unpaired t-tests were performed by GraphPad Prism 9.3 software.

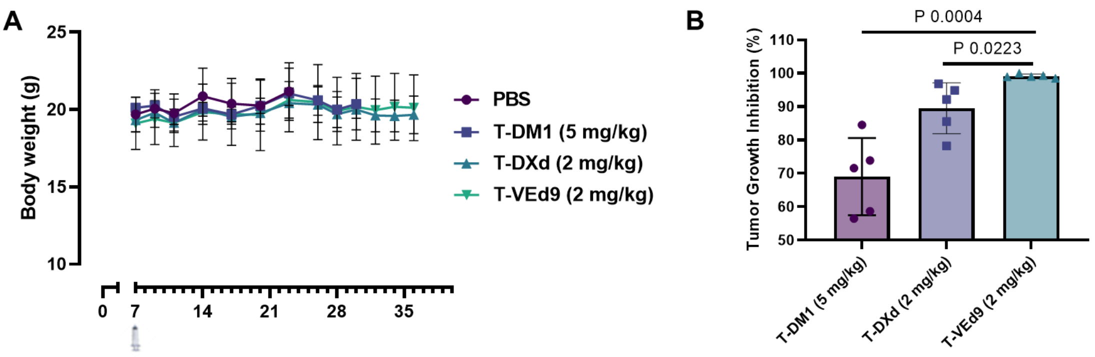

Figure S12. (A) Body weight change and (B) Tumor growth inhibition in HER2-heterogeneity co-inoculation xenograft model following a single intravenous ADC. Error bars represent S.D. Unpaired t-tests were performed by GraphPad Prism 9.3 software.

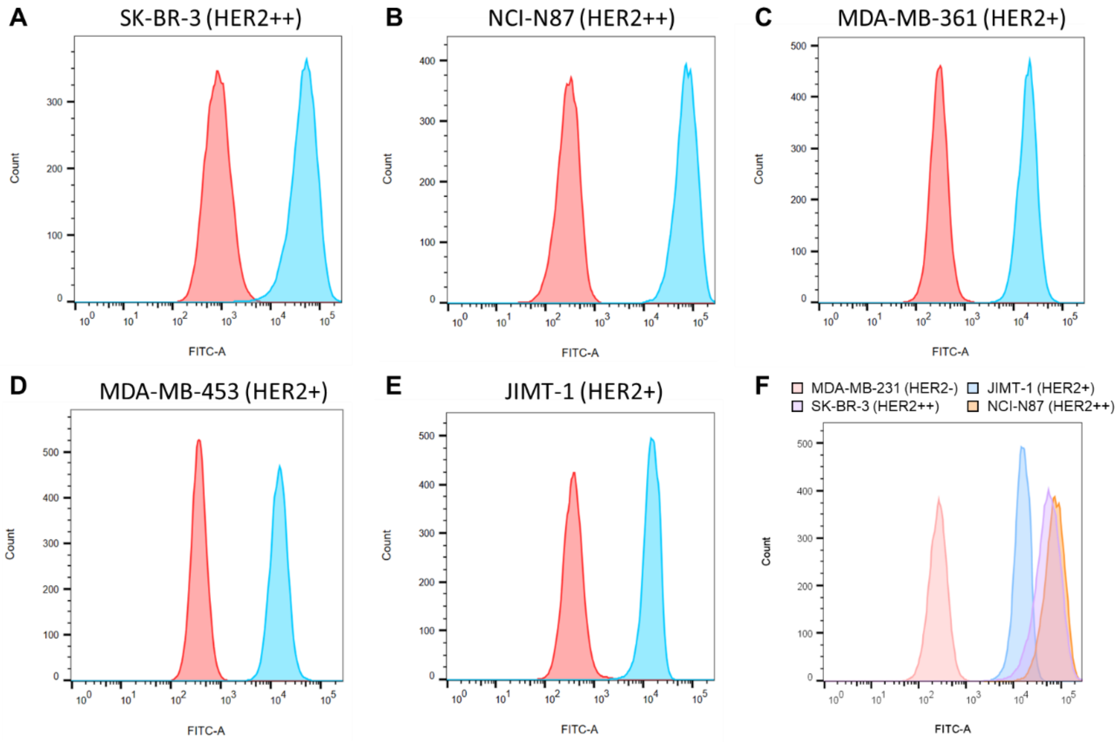

Figure S13. Analysis of HER2 expression level in cancer cells used in this study by flow cytometry. (A) SK-BR-3. (B) NCI-N87. (C) MDA-MB-361. (D) MDA-MB-453. (E) JIMT-1. (F) Merge graph of cells used in xenograft model at the same excitation/detection intensity.

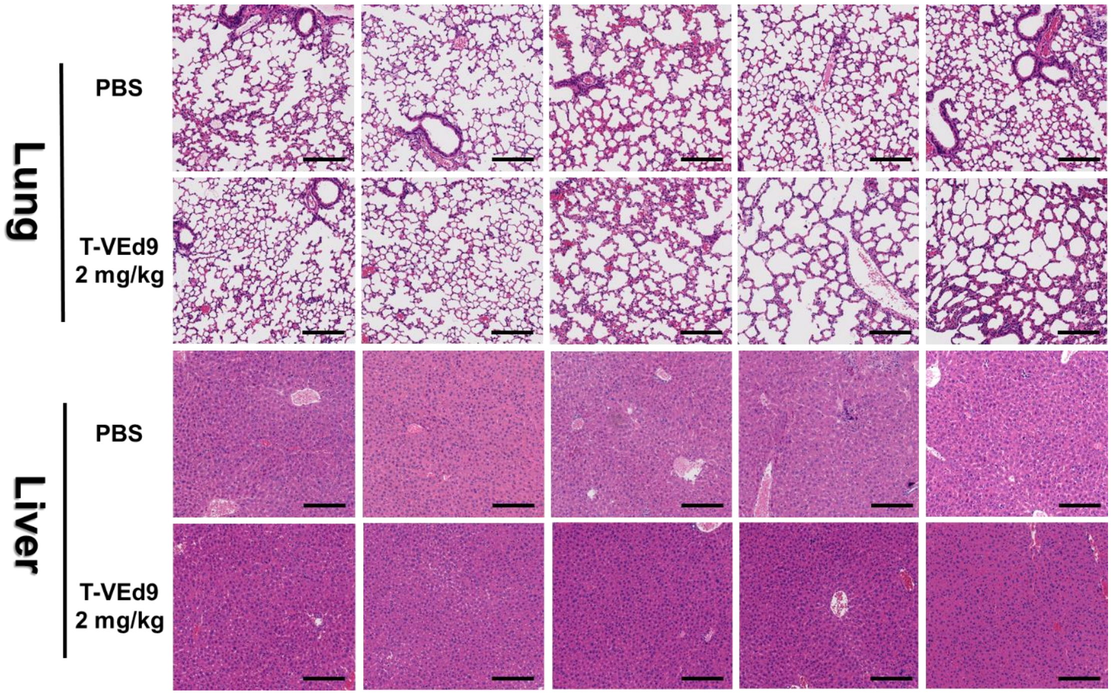

Figure S14. Hematoxylin and Eosin (H&E) staining of mouse liver and lung tissue after receiving treatment of PBS and T-VEd9. Scale bar: 25 μm.

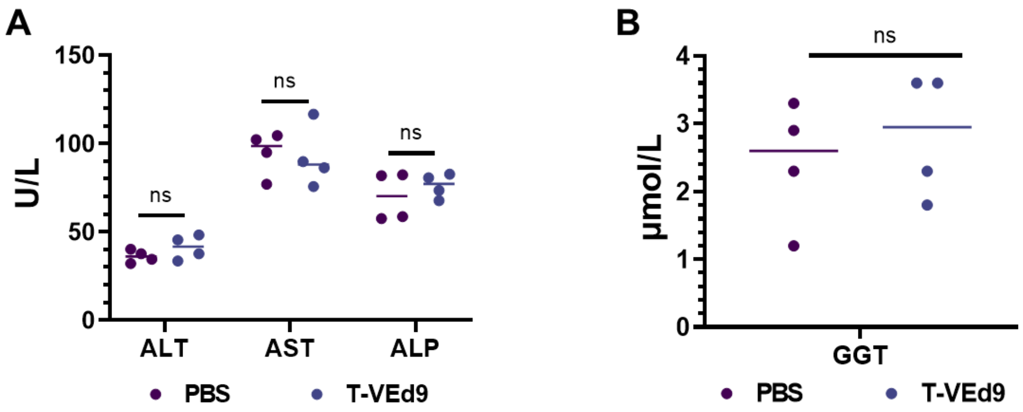

Figure S15. Blood chemistry parameters (ALT, AST, ALP, and GGT) post-injection of PBS control and T-VEd9 (2 mg/kg). Error bars represent S.D. The graphs and unpaired t-tests were performed by GraphPad Prism 9.3 software.

Table S1. Test the R^2 score and RMSE of GAT and machine learning models for B and K scores.

Model	B score		K score	
	**R^2**	**RMSE**	**R^2**	**RMSE**
RF	**0.663**	**0.465**	**0.837**	**0.626**
SVR	**0.714**	**0.428**	**0.840**	**0.621**
XGBOOST	**0.716**	**0.427**	**0.834**	**0.632**
Bayers	**0.725**	**0.420**	**0.817**	**0.644**
B-K score model	**0.811**	**0.374**	**0.845**	**0.486**

Table S2. Estimated physicochemical properties and reported bystander of ADC payloads currently in clinic or development.

Payload	Calculated from structure (ADMET 2.0) [a]					B Score	Reported bystander?
	M.W.	TPSA	nHA	nHD	cLogD		
**Calicheamicin**	1289.31	308.77	24	7	3.058	1.397	N/A
**Lys-SMCC-DM1**	1102.47	294.28	21	8	1.938	1.265	No [1]
**MMAE**	717.5	149.54	12	4	3.091	1.699	Yes [2]
**MMAF**	731.48	166.61	13	4	2.489	1.349	No [2]
**SN-38**	392.14	101.65	7	2	1.577	2.220	Yes [2]
**DXd**	493.16	130.75	9	3	1.362	2.143	Yes [1]
**DM4**	765.31	156.47	13	2	2.756	1.879	Yes [2]
**SG3199**	584.26	102.26	10	0	3.863	2.166	Yes [2]
**Eribulin**	729.41	146.39	12	3	3.201	1.738	Yes [3]
**DUBA**	526.14	107.17	8	3	3.469	2.028	Yes [4]
**α-Amanitin**	918.35	386.87	24	14	-0.839	-0.163	No [5]
**β-Amanitin**	919.34	381.08	24	13	-0.935	-0.082	No [5]

[a.] The conventional physical and chemical properties are calculated by ADMETlab2.0.

Table S3. B Score, K Score and B.K. Score of DXd and Ed1-Ed11 calculated by our B Score and K Score model.[a]

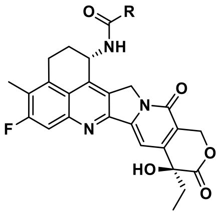

Compound	R	B Score	PAMPA [b]	K Score	B.K. Score	pIC_{50} [c]
**DXd**	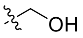	2.143	3.12	-0.246	1.897	1.131
**Ed1**	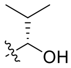	2.113	N.T.	-0.827	1.286	0.888
**Ed2**	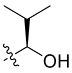	2.113	2.67	-0.827	1.286	1.376
**Ed3**	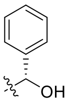	2.062	N.T.	-0.638	1.424	0.961
**Ed4**	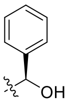	2.062	3.43	-0.638	1.424	1.306
**Ed5**	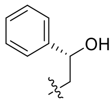	2.037	N.T.	-0.669	1.368	0.753
**Ed6**	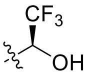	2.265	N.T.	-0.469	1.796	1.219
**Ed7**	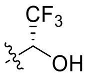	2.265	4.96	-0.469	1.796	1.382
**Ed8**	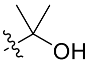	2.310	5.74	0.078	2.387	1.841
**Ed9**	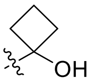	2.273	5.66	0.131	2.405	1.881
**Ed10**	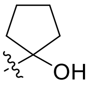	2.278	N.T.	-0.032	2.246	1.750
**Ed11**	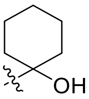	2.283	6.12	-0.229	2.054	1.753

[a.] The higher score is more satisfactory; the color scale from red to green indicates a low to high score; [b.] 10^{-6} cm/s, N.T., not tested; [c.] Normalized from IC_{50} (μM) against SKBR-3.

Table S4. Activities of DXd and Ed1-Ed11 in inhibition of the SKBR-3 and MDA-MB-231 proliferation.

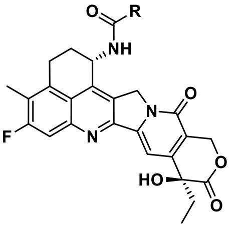

Compound	R	IC_{50} (nM) [a]	
		SKBR-3	MDA-MB-231
**DXd**	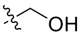	73.93 ± 17.13	25.77
**Ed1**	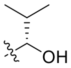	129.41	N.T.
**Ed2**	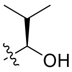	42.11	8.32
**Ed3**	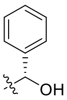	109.43 ± 12.19	N.T.[b]
**Ed4**	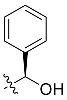	49.47 ± 8.32	4.41
**Ed5**	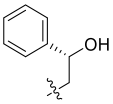	176.57 ± 15.84	N.T.
**Ed6**	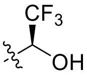	60.37	9.46
**Ed7**	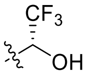	41.53	6.11
**Ed8**	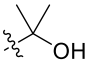	14.43 ± 3.79	4.21
**Ed9**	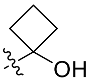	13.14 ± 4.24	2.69
**Ed10**	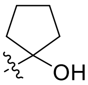	17.77 ± 3.69	5.17
**Ed11**	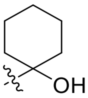	17.67 ± 5.34	5.43

[a.] The data are generated from more than two independent experiments; [b.] N.T., not tested.

Table S5. Statistical significance analysis of tumor volume after treatment in cell-driven xenograft model.

Main Figures	Method	Comparison	P value	Significance [a]
**Figure S9B**	Two-tailed unpaired t-test	**Day 23**<br>10 mpk T-VEd9 vs. 10 mpk T-DXd<br>10 mpk T-VEd9 vs. 10 mpk Tras	0.0167<br>0.0001	*<br>***
**Figure 5A**	Two-tailed unpaired t-test	**Day 38**<br>1 mpk T-VEd9 vs. 1 mpk T-DXd<br>1 mpk T-VEd9 vs. 3 mpk T-DXd	0.0032<br>/	**<br>ns
**Figure 5C**	Two-tailed unpaired t-test	**Day 23**<br>2 mpk T-VEd9 vs. 2 mpk T-DXd<br>2 mpk T-VEd9 vs. 5 mpk T-DM1	0.0223<br>0.0004	*<br>***
		**Day 30**<br>2 mpk T-VEd9 vs. 2 mpk T-DXd<br>2 mpk T-VEd9 vs. 5 mpk T-DM1	0.0147<br>0.0013	*<br>**
		**Day 36**<br>2 mpk T-VEd9 vs. 2 mpk T-DXd	0.0174	*

[a.] *P < 0.05; **P < 0.01; ***P < 0.001; n.s., not significant.

Synthesis

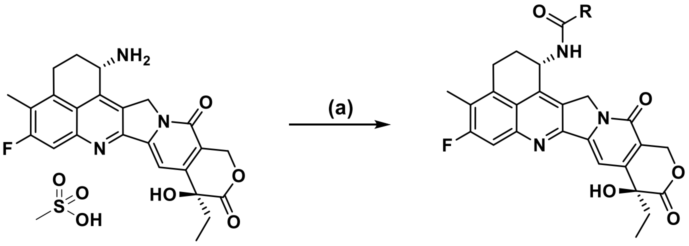

Synthesis of **exatecan derivatives (Eds)**, (a) EDC·HCl, HOBT, DIPEA, DMF, room temp, 4h.

General procedure: The corresponding acids (0.044 mmol), HOBT (7 mg, 0.051mmol), EDC·HCl (10 mg, 0.051mmol), and DIPEA (10 μL, 0.051 mmol) was dissolved in 3 mL DMF in the flask, stirred at room temperature for 30min and then added the 1 mL DMF solution of exatecan mesylate (18 mg, 0.034 mmol) and DIPEA (15 μL, 0.085 mmol) into the flask, react at room temperature for another 4h. After TLC monitoring, the mixture was concentrated under a vacuum. The residue was diluted with water (25 mL) and extracted with ethyl acetate (25 mL × 3), washed with saturated brine (25 mL × 2), and dried over anhydrous sodium sulfate. The crude product was purified by column chromatography to afford the product.

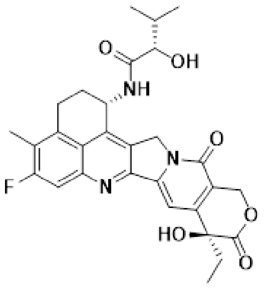

(S)-N-((1S,9S)-9-ethyl-5-fluoro-9-hydroxy-4-methyl-10,13-dioxo-2,3,9,10,13,15-hexahydro-1H,12H-benzo[de]pyrano[3',4':6,7]indolizino[1,2-b]quinolin-1-yl)-2-hydroxy-3-methylbutanamide (**Ed1**)

General procedure. Yield: 70.35%; purity: 98.98% (R_t = 8.815 min). ^{1}H NMR (400 MHz, DMSO-d_6) δ 8.29 (d, J = 8.8 Hz, 1H), 7.71 (d, J = 10.9 Hz, 1H), 7.26 (s, 1H), 5.56 – 5.47 (m, 1H), 5.38 (s, 3H), 5.21 – 5.03 (m, 2H), 3.79 (d, J = 3.9 Hz, 1H), 3.11 (q, J = 6.4 Hz, 2H), 2.33 (d, J = 1.9 Hz, 3H), 2.16 (h, J = 6.2 Hz, 1H), 2.07 (dt, J = 8.4, 3.9 Hz, 1H), 2.01 (ddd, J = 13.5, 6.6, 2.6 Hz, 1H), 1.82 (hept, J = 7.1 Hz, 2H), 0.89 (d, J = 6.9 Hz, 3H), 0.81 (m, 6H). ^{13}C NMR (101 MHz, DMSO-d_6) δ 174.03, 172.98, 160.92, 157.18, 152.79, 150.52, 148.37, 145.74, 141.20, 137.02, 136.96, 126.16, 124.20, 122.38, 119.68, 110.43, 97.17, 76.13, 72.89, 65.76, 50.05, 44.96, 31.97, 30.74, 28.44, 19.67, 16.93, 11.48, 8.31. HRMS (ESI) for $C_{29}H_{31}FN_3O_6$ $[M+H]^+$, calcd: 536.2197, found: 536.2178.

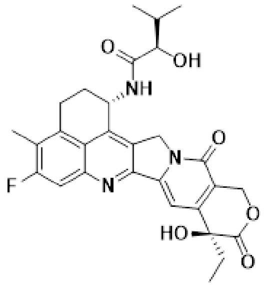

(R)-N-((1S,9S)-9-ethyl-5-fluoro-9-hydroxy-4-methyl-10,13-dioxo-2,3,9,10,13,15-hexahydro-1H,12H-benzo[de]pyrano[3',4':6,7]indolizino[1,2-b]quinolin-1-yl)-2-hydroxy-3-methylbutanamide (**Ed2**)

General procedure. Yield: 67.63%; purity: 100% (R_t = 8.116 min). ^{1}H NMR (400 MHz, DMSO-*d*$_6$) δ 8.33 (d, *J* = 8.6 Hz, 1H), 7.73 (dd, *J* = 11.0, 2.9 Hz, 1H), 7.26 (d, *J* = 1.3 Hz, 1H), 6.49 (s, 1H), 5.50 (dt, *J* = 9.7, 5.2 Hz, 1H), 5.38 (s, 2H), 5.31 – 5.22 (m, 1H), 5.20 – 5.07 (m, 2H), 3.69 (d, *J* = 4.6 Hz, 1H), 3.20 – 3.02 (m, 2H), 2.34 (d, *J* = 2.0 Hz, 3H), 2.17 – 2.06 (m, 2H), 2.04 – 1.97 (m, 1H), 1.90 – 1.74 (m, 2H), 0.88 (d, *J* = 6.8 Hz, 3H), 0.85 – 0.80 (m, 6H). ^{13}C NMR (101 MHz, DMSO-*d*$_6$) δ 173.96, 173.01, 163.39, 160.92, 157.22, 152.86, 150.47, 148.54, 145.79, 140.79, 136.91, 130.19, 126.29, 124.07, 122.31, 119.68, 110.50, 97.16, 76.14, 72.88, 65.79, 50.34, 45.00, 31.97, 30.79, 28.34, 23.69, 19.74, 17.47, 11.54, 8.29. HRMS (ESI) for $C_{29}H_{31}FN_3O_6$ $[M+H]^+$, calcd: 536.2197, found: 536.2182.

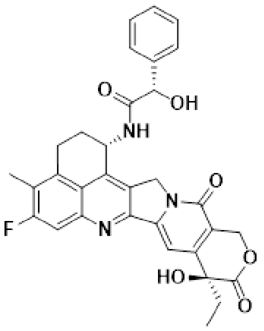

(S)-N-((1S,9S)-9-ethyl-5-fluoro-9-hydroxy-4-methyl-10,13-dioxo-2,3,9,10,13,15-hexahydro-1H,12H-benzo[de]pyrano[3',4':6,7]indolizino[1,2-b]quinolin-1-yl)-2-hydroxy-2-phenylacetamide (**Ed3**)

General procedure. Yield: 46.15%; purity: 96.83% (R_t = 9.090 min). ^{1}H NMR (400 MHz, DMSO-*d*$_6$) δ 8.63 (d, *J* = 8.7 Hz, 1H), 7.73 (d, *J* = 10.9 Hz, 1H), 7.42 – 7.39 (m, 2H), 7.30 – 7.26 (m, 3H), 7.24 – 7.20 (m, 1H), 6.50 (s, 1H), 6.21 (d, *J* = 4.4 Hz, 1H), 5.46 (q, *J* = 6.3 Hz, 1H), 5.40 (s, 2H), 5.08 (d, *J* = 3.1 Hz, 2H), 5.03 (d, *J* = 4.2 Hz, 1H), 3.15 – 3.02 (m, 2H), 2.34 (d, *J* = 1.7 Hz, 3H), 2.14 (h, *J* = 6.0 Hz, 1H), 2.00 – 1.93 (m, 1H), 1.84 (dt, *J* = 16.5, 7.0 Hz,

2H), 0.84 (t, *J* = 7.3 Hz, 3H). ^{13}C NMR (101 MHz, DMSO-*d*6) δ 173.02, 172.83, 163.41, 160.94, 157.16, 152.82, 150.49, 148.52, 145.72, 141.61, 141.06, 136.99, 130.19, 128.58, 128.05, 127.09, 126.21, 124.23, 122.33, 119.66, 110.23, 97.15, 74.41, 72.89, 65.78, 50.07, 45.20, 30.80, 28.15, 23.91, 11.50, 8.31. HRMS (ESI) for $C_{32}H_{29}FN_3O_6$ [M+H]$^+$, calcd: 570.2040, found: 570.2026.

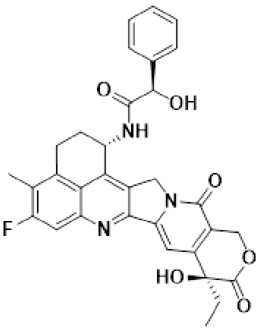

(R)-N-((1S,9S)-9-ethyl-5-fluoro-9-hydroxy-4-methyl-10,13-dioxo-2,3,9,10,13,15-hexahydro-1H,12H-benzo[de]pyrano[3',4':6,7]indolizino[1,2-b]quinolin-1-yl)-2-hydroxy-2-phenylacetamide (**Ed4**)

General procedure. Yield: 51.26%; purity: 93.82% (R_t = 8.459 min). ^{1}H NMR (400 MHz, DMSO-*d*6) δ 8.66 (d, *J* = 8.7 Hz, 1H), 7.74 (d, *J* = 10.9 Hz, 1H), 7.49 – 7.45 (m, 2H), 7.33 – 7.28 (m, 2H), 7.28 – 7.21 (m, 2H), 6.49 (s, 1H), 6.15 (d, *J* = 4.8 Hz, 1H), 5.45 (dd, *J* = 9.5, 4.4 Hz, 1H), 5.41 (s, 2H), 5.12 (s, 2H), 4.99 (d, *J* = 4.7 Hz, 1H), 3.09 (s, 2H), 2.34 (d, *J* = 1.8 Hz, 3H), 2.06 (d, *J* = 10.6 Hz, 1H), 1.95 (dt, *J* = 13.7, 7.0 Hz, 1H), 1.90 – 1.77 (m, 2H), 0.85 (t, *J* = 7.3 Hz, 3H). ^{13}C NMR (101 MHz, DMSO-*d*6) δ 173.04, 172.69, 163.39, 160.92, 157.24, 152.90, 150.48, 148.40, 145.79, 141.76, 140.85, 136.91, 130.20, 128.60, 127.97, 127.33, 124.26, 122.35, 119.68, 110.51, 97.14, 74.10, 72.89, 65.83, 50.49, 45.38, 30.79, 28.27, 23.88, 11.55, 8.31. HRMS (ESI) for $C_{32}H_{29}FN_3O_6$ [M+H]$^+$, calcd: 570.2040, found: 570.2024.

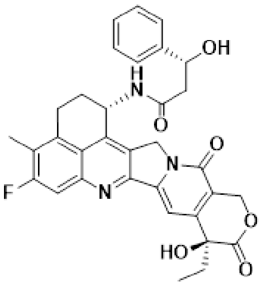

(R)-N-((1S,9S)-9-ethyl-5-fluoro-9-hydroxy-4-methyl-10,13-dioxo-2,3,9,10,13,15-hexahydro-1H,12H-benzo[de]pyrano[3',4':6,7]indolizino[1,2-b]quinolin-1-yl)-3-hydroxy-3-phenylpropanamide (**Ed5**)

General procedure. Yield: 43.27%; purity: 96.60% (R_t = 7.914 min). ^{1}H NMR (400 MHz, DMSO-d_6) δ 8.32 (d, *J* = 8.6 Hz, 1H), 7.75 (d, *J* = 11.0 Hz, 1H), 7.28 – 7.25 (m, 2H), 7.24 – 7.22 (m, 3H), 7.17 – 7.10 (m, 1H), 6.50 (s, 1H), 5.49 (dt, *J* = 8.6, 4.2 Hz, 1H), 5.40 (s, 2H), 5.33 (d, *J* = 4.5 Hz, 1H), 5.22 (d, *J* = 19.1 Hz, 1H), 5.05 (d, *J* = 19.1 Hz, 1H), 4.93 (td, *J* = 8.6, 7.1, 3.3 Hz, 1H), 3.05 (dt, *J* = 17.1, 4.8 Hz, 1H), 2.99 – 2.89 (m, 1H), 2.54 – 2.49 (m, 1H), 2.39 (dd, *J* = 13.6, 5.7 Hz, 1H), 2.34 (d, *J* = 1.9 Hz, 3H), 2.00 – 1.94 (m, 2H), 1.89 – 1.77 (m, 2H), 0.84 (t, *J* = 7.3 Hz, 3H). ^{13}C NMR (101 MHz, DMSO-d_6) δ 173.05, 170.22, 163.41, 160.94, 157.17, 152.88, 150.43, 148.49, 145.80, 145.18, 140.42, 136.86, 130.19, 128.61, 128.24, 127.13, 126.44, 124.18, 121.78, 119.70, 110.54, 97.13, 72.88, 70.38, 65.81, 49.99, 45.74, 43.79, 30.85, 27.82, 23.14, 11.56, 8.30. HRMS (ESI) for $C_{33}H_{31}FN_3O_6$ [M+H]$^+$, calcd: 584.2197, found: 584.2176.

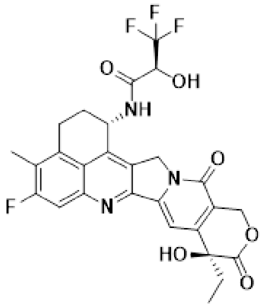

(S)-N-((1S,9S)-9-ethyl-5-fluoro-9-hydroxy-4-methyl-10,13-dioxo-2,3,9,10,13,15-hexahydro-1H,12H-benzo[de]pyrano[3',4':6,7]indolizino[1,2-b]quinolin-1-yl)-3,3,3-trifluoro-2-hydroxypropanamide (**Ed6**)

General procedure. Yield: 31.25%; purity: 94.86% (R_t = 8.996 min). ^{1}H NMR (400 MHz, DMSO-d_6) δ 8.93 (d, *J* = 8.6 Hz, 1H), 7.75 (d, *J* = 10.9 Hz, 1H), 7.27 (d, *J* = 4.3 Hz, 1H), 7.13 (dd, *J* = 6.9, 3.8 Hz, 1H), 6.49 (s, 1H), 5.55 (dt, *J* = 9.0, 5.1 Hz, 1H), 5.39 (d, *J* = 4.5 Hz, 2H), 5.23 – 5.04 (m, 2H), 4.58 (p, *J* = 7.6 Hz, 1H), 3.12 (d, *J* = 7.1 Hz, 2H), 2.39 – 2.30 (m, 3H), 2.15 (dtt, *J* = 19.0, 13.2, 5.9 Hz, 2H), 1.88 – 1.76 (m, 2H), 0.83 (t, *J* = 7.2 Hz, 3H). HRMS (ESI) for $C_{27}H_{24}F_4N_3O_6$ [M+H]$^+$, calcd: 562.1601, found: 562.1584.

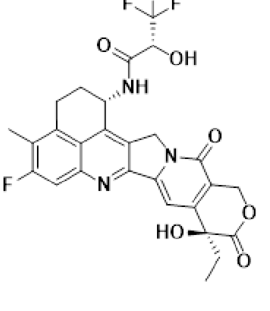

(R)-N-((1S,9S)-9-ethyl-5-fluoro-9-hydroxy-4-methyl-10,13-dioxo-2,3,9,10,13,15-hexahydro-1H,12H-benzo[de]pyrano[3',4':6,7]indolizino[1,2-b]quinolin-1-yl)-3,3,3-trifluoro-2-hydroxypropanamide (**Ed7**)

General procedure. Yield: 43.68%; purity: 95.12% (R_t = 8.219 min). ^{1}H NMR (400 MHz, DMSO-d_6) δ 8.91 (d, *J* = 8.6 Hz, 1H), 7.75 (d, *J* = 10.9 Hz, 1H), 7.27 (s, 1H), 7.17 (d, *J* = 6.8 Hz, 1H), 6.50 (s, 1H), 5.54 (dt, *J* = 8.7, 5.1 Hz, 1H), 5.38 (s, 2H), 5.26 – 4.97 (m, 2H), 4.58 (qd, *J* = 7.5, 2.9 Hz, 1H), 3.19 – 3.03 (m, 2H), 2.35 (d, *J* = 1.9 Hz, 3H), 2.25 – 2.15 (m, 1H), 2.10 (td, *J* = 14.2, 13.1, 7.3 Hz, 1H), 1.83 (hept, *J* = 7.1 Hz, 2H), 0.83 (t, *J* = 7.5 Hz, 3H). HRMS (ESI) for $C_{27}H_{24}F_4N_3O_6$ [M+H]$^+$, calcd: 562.1601, found: 562.1585.

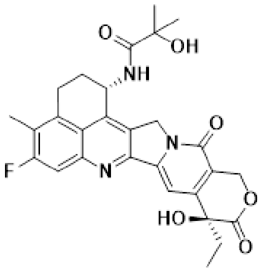

N-((1S,9S)-9-ethyl-5-fluoro-9-hydroxy-4-methyl-10,13-dioxo-2,3,9,10,13,15-hexahydro-1H,12H-benzo[de]pyrano[3',4':6,7]indolizino[1,2-b]quinolin-1-yl)-2-hydroxy-2-methylpropanamide (**Ed8**)

General procedure. Yield: 71.23%; purity: 97.40% (R_t = 7.468 min). ^{1}H NMR (400 MHz, DMSO-d_6) δ 8.29 (dd, *J* = 9.2, 1.8 Hz, 1H), 7.63 (d, *J* = 10.9 Hz, 1H), 7.23 (s, 1H), 6.48 (s, 1H), 5.72 (s, 1H), 5.47 (d, *J* = 11.5 Hz, 2H), 5.37 (d, *J* = 1.7 Hz, 2H), 5.16 (d, *J* = 18.9 Hz, 1H), 4.84 (d, *J* = 18.9 Hz, 1H), 3.22 – 2.98 (m, 2H), 2.28 (d, *J* = 1.9 Hz, 3H), 2.11 (q, *J* = 6.6, 5.6 Hz, 2H), 1.89 – 1.73 (m, *J* = 7.2 Hz, 2H), 1.42 (s, 3H), 1.32 (s, 3H), 0.83 (t, *J* = 7.2 Hz, 3H). ^{13}C NMR (101 MHz, DMSO-d_6) δ 176.98, 172.99, 163.25, 160.78, 157.10, 152.76, 150.41, 148.38, 145.52, 142.16, 136.87, 130.19, 125.42, 123.88, 122.28, 119.56, 110.19, 97.11, 72.88, 72.77, 65.77, 50.41, 46.20, 30.88, 28.84, 28.65, 27.93, 25.09, 11.47, 8.29. HRMS (ESI) for $C_{28}H_{29}FN_3O_6$ [M+H]$^+$, calcd: 522.2040, found: 522.2025.

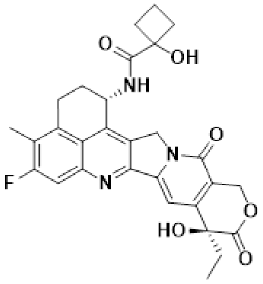

N-((1S,9S)-9-ethyl-5-fluoro-9-hydroxy-4-methyl-10,13-dioxo-2,3,9,10,13,15-hexahydro-1H,12H-benzo[de]pyrano[3',4':6,7]indolizino[1,2-b]quinolin-1-yl)-1-hydroxycyclobutane-1-carboxamide (**Ed9**)

General procedure. Yield: 68.56%; purity: 95.52% (R_t = 7.892 min). ^{1}H NMR (400 MHz, DMSO-d_6) δ 8.21 (d, *J* = 9.1 Hz, 1H), 7.62 (d, *J* = 10.9 Hz, 1H), 7.22 (s, 1H), 6.48 (s, 1H), 6.10 (s, 1H), 5.50 (q, *J* = 7.5 Hz, 1H), 5.37 – 5.34 (m, 2H), 5.10 – 5.00 (m, 1H), 4.83 (d, *J* = 18.9 Hz, 1H), 3.16 (dt, *J* = 16.8, 5.2 Hz, 1H), 3.03 (dt, *J* = 16.2, 7.3 Hz, 1H), 2.63 (ddt, *J* = 10.8, 7.1, 3.2 Hz, 1H), 2.27 (d, *J* = 1.9 Hz, 3H), 2.18 – 1.92 (m, 5H), 1.86 – 1.77 (m, 4H), 0.85 – 0.81 (m, 3H). ^{13}C NMR (101 MHz, DMSO-d_6) δ 175.60, 172.99, 163.23, 160.76, 157.07, 152.75, 150.39, 148.36, 145.52, 142.32, 136.87, 130.18, 125.38, 122.29, 119.54, 110.17, 97.08, 75.22, 72.88, 65.77, 50.34, 46.08, 35.38, 35.17, 30.88, 28.78, 25.11, 13.55, 11.41, 8.29. HRMS (ESI) for $C_{29}H_{29}FN_3O_6$ [M+H]$^+$, calcd: 534.2040, found: 534.2026.

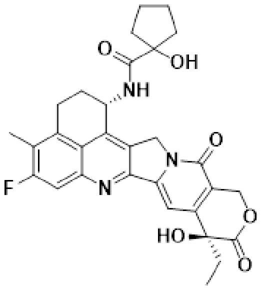

N-((1S,9S)-9-ethyl-5-fluoro-9-hydroxy-4-methyl-10,13-dioxo-2,3,9,10,13,15-hexahydro-1H,12H-benzo[de]pyrano[3',4':6,7]indolizino[1,2-b]quinolin-1-yl)-1-hydroxycyclopentane-1-carboxamide (**Ed10**)

General procedure. Yield: 74.77%; purity: 96.29% (R_t = 8.251 min). ^{1}H NMR (400 MHz, DMSO-d_6) δ 8.41 (dd, *J* = 9.3, 2.0 Hz, 1H), 7.62 (d, *J* = 10.9 Hz, 1H), 7.23 (s, 1H), 6.49 (s, 1H), 5.49 (q, *J* = 7.8 Hz, 1H), 5.37 (d, *J* = 2.7 Hz, 2H), 5.33 (s, 1H), 5.15 (d, *J* = 19.0 Hz, 1H), 4.79 (d, *J* = 18.9 Hz, 1H), 3.16 (dt, *J* = 16.8, 4.8 Hz, 1H), 3.03 (dt, *J* = 16.4, 7.5 Hz, 1H), 2.27 (d, *J* = 2.0 Hz, 3H), 2.12 (q, *J* = 6.8 Hz, 2H), 2.08 – 2.00 (m, 2H), 1.84 (tt, *J* = 14.3, 7.2 Hz, 3H), 1.76 – 1.64 (m, 5H), 0.83 (t, *J* = 7.2 Hz, 3H). ^{13}C NMR (101 MHz, DMSO-d_6) δ 176.93, 172.98, 163.23, 160.75, 157.06, 152.74, 150.39, 148.22, 145.49, 142.37, 136.86, 136.80, 130.18, 125.32, 124.03, 122.27, 119.58, 110.40, 97.08, 82.91, 72.88, 65.78, 50.39, 46.33, 30.90, 28.91, 25.21, 24.97, 24.86, 11.46, 8.30. HRMS (ESI) for $C_{30}H_{31}FN_3O_6$ [M+H]$^+$, calcd: 548.2197, found: 548.2179.

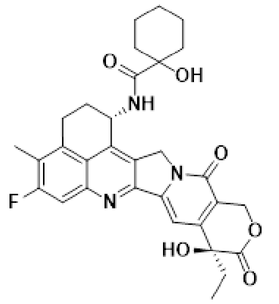

N-((1S,9S)-9-ethyl-5-fluoro-9-hydroxy-4-methyl-10,13-dioxo-2,3,9,10,13,15-hexahydro-1H,12H-benzo[de]pyrano[3',4':6,7]indolizino[1,2-b]quinolin-1-yl)-1-hydroxycyclohexane-1-carboxamide (**Ed11**)

General procedure. Yield: 64.53%; purity: 93.68% (R_t = 9.004 min). ^{1}H NMR (400 MHz, DMSO-d_6) δ 8.24 (d, *J* = 9.1 Hz, 1H), 7.65 (d, *J* = 10.8 Hz, 1H), 7.23 (s, 1H), 6.48 (s, 1H), 5.46 (q, *J* = 7.9 Hz, 1H), 5.37 (d, *J* = 2.3 Hz, 2H), 5.17 – 5.09 (m, 2H), 4.88 (d, *J* = 18.9 Hz, 1H), 3.14 (dt, *J* = 17.0, 5.6 Hz, 1H), 3.03 (dt, *J* = 16.8, 7.3 Hz, 1H), 2.29 (d, *J* = 1.9 Hz, 3H), 2.11 (h, *J* = 6.1, 5.6 Hz, 2H), 1.85 – 1.78 (m, 2H), 1.77 – 1.72 (m, 2H), 1.52 (d, *J* = 16.4 Hz, 8H), 0.85 – 0.81 (m, 3H). ^{13}C NMR (101 MHz, DMSO-d_6) δ 177.45, 172.98, 163.27, 160.80, 157.09, 152.75, 150.39, 148.41, 145.57, 141.91, 136.83, 130.18, 125.68, 123.87, 122.28, 119.60, 110.42, 97.09, 74.34, 72.86, 65.79, 50.33, 45.89, 34.60, 34.01, 30.91, 28.74, 25.57, 24.73, 21.34, 21.27, 11.47, 8.28. HRMS (ESI) for $C_{31}H_{33}FN_3O_6$ $[M+H]^+$, calcd: 562.2353, found: 562.2338.

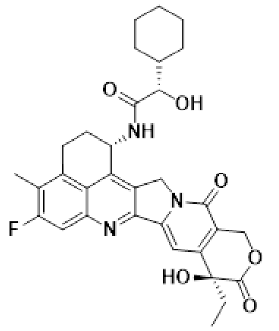

(S)-2-cyclohexyl-N-((1S,9S)-9-ethyl-5-fluoro-9-hydroxy-4-methyl-10,13-dioxo-2,3,9,10,13,15-hexahydro-1H,12H-benzo[de]pyrano[3',4':6,7]indolizino[1,2-b]quinolin-1-yl)-2-hydroxyacetamide (**Ed12**)

General procedure. Yield: 46%; purity: 100% (R_t = 10.465 min). ^{1}H NMR (400 MHz, Methanol-d_4) δ 7.49 (s, 1H), 7.46 (d, *J* = 10.7 Hz, 1H), 5.54 – 5.47 (m, 2H), 5.32 (d, *J* = 1.4 Hz, 1H), 5.13 (d, *J* = 19.0 Hz, 1H), 4.98 – 4.88 (m, 1H), 3.86 (d, *J* = 4.6 Hz, 1H), 3.25 – 3.04 (m, 2H), 2.32 (d, *J* = 1.8 Hz, 3H), 2.30 – 2.13 (m, 3H), 2.05 – 1.95 (m, 1H), 1.90 (qd, *J* = 7.0, 1.9 Hz, 3H), 1.77 (d, *J* = 11.9 Hz, 4H), 1.65 (d, *J* = 11.1 Hz, 4H), 0.96 (t, *J* = 7.3 Hz, 3H). HRMS (ESI) for $C_{32}H_{35}FN_3O_6$ $[M+H]^+$, calcd: 576.2510, found: 576.2497.

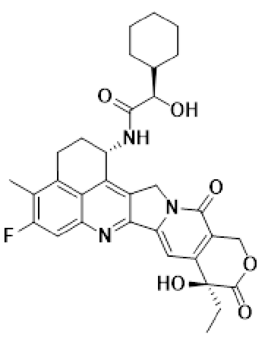

(R)-2-cyclohexyl-N-((1S,9S)-9-ethyl-5-fluoro-9-hydroxy-4-methyl-10,13-dioxo-2,3,9,10,13,15-hexahydro-1H,12H-benzo[de]pyrano[3',4':6,7]indolizino[1,2-b]quinolin-1-yl)-2-hydroxyacetamide (**Ed13**)

General procedure. Yield: 46%; purity: 100% (R_t = 9.562 min). ^{1}H NMR (400 MHz, Methanol-d_4) δ 7.52 (s, 1H), 7.41 (d, J = 10.8 Hz, 1H), 5.60 – 5.52 (m, 2H), 5.33 – 5.27 (m, 1H), 5.10 – 5.03 (m, 1H), 4.46 (d, J = 18.7 Hz, 1H), 4.07 (d, J = 3.3 Hz, 1H), 3.26 – 2.97 (m, 2H), 2.29 (d, J = 1.8 Hz, 3H), 2.25 (h, J = 3.9 Hz, 2H), 2.07 – 1.95 (m, 2H), 1.91 (q, J = 7.3 Hz, 3H), 1.73 (td, J = 23.2, 22.4, 9.6 Hz, 6H), 1.56 (s, 2H), 0.97 (t, J = 7.4 Hz, 3H). HRMS (ESI) for $C_{32}H_{35}FN_3O_6$ $[M+H]^+$, calcd: 576.2510, found: 576.2494.

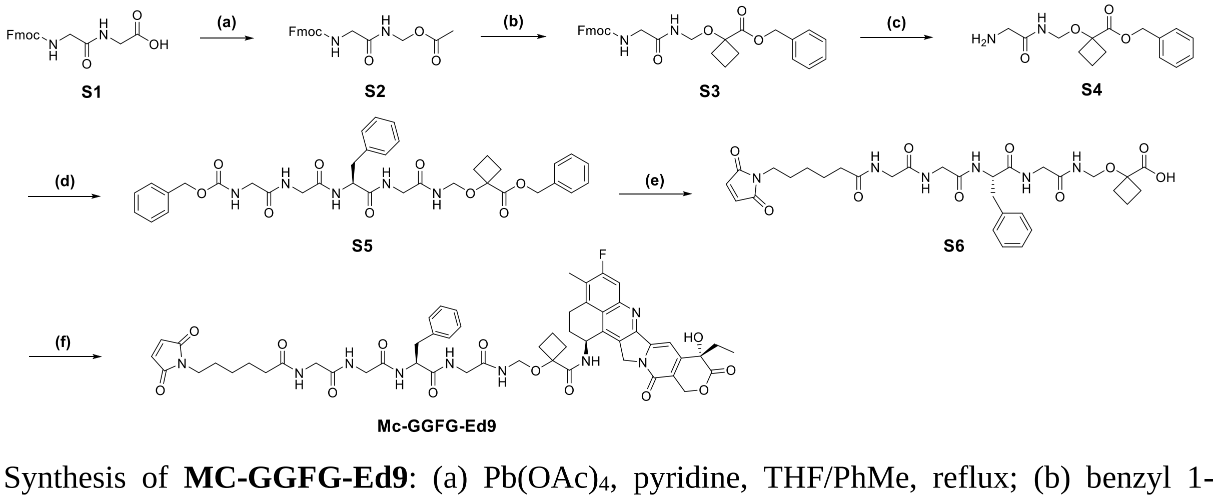

Synthesis of **MC-GGFG-Ed9**: (a) $Pb(OAc)_4$, pyridine, THF/PhMe, reflux; (b) benzyl 1-hydroxycyclobutane-1-carboxylate, PPTS, DCM/THF, reflux; (c) 20% piperidine in DMF, room temp; (d) Z-GGF-OH, EDC·HCl, HOBT, DIPEA, DMF, room temp; (e) 1) Pd/C, H_2, MeOH, room temp; 2) Maleimide-NHS, DIPEA, DMF, room temp; (f) exatecan mesylate, EDC·HCl, HOBT, DIPEA, DMF, room temp.

(2-((((9H-fluoren-9-yl)methoxy)carbonyl)amino)acetamido)methyl acetate (**S2**): Fmoc-Gly-Gly-OH (2.8 g, 8 mmol), $Pb(OAc)_4$ (4.4 g, 10 mmol), pyridine (900 μL, 10 mmol), 50mL

tetrahydrofuran and 15 mL toluene was added to a 250 mL flask. The mixture was heated to reflux (85 °C) for 2.5h under a nitrogen atmosphere. After filtration and concentration, the residue was purified by column chromatography to afford the product as the white solid (1.8 g, yield: 61.1%). ESI-MS: m/z = 391.1 $[M+Na]^+$. ^{1}H NMR (400 MHz, DMSO-d_6) δ 8.90 (t, *J* = 7.0 Hz, 1H), 7.86 (d, *J* = 7.5 Hz, 2H), 7.68 (d, *J* = 7.5 Hz, 2H), 7.55 (t, *J* = 6.1 Hz, 1H), 7.38 (td, *J* = 7.5, 1.2 Hz, 2H), 7.30 (td, *J* = 7.5, 1.2 Hz, 2H), 5.06 (d, *J* = 6.9 Hz, 2H), 4.26 (d, *J* = 7.4 Hz, 2H), 4.22 – 4.17 (m, 1H), 3.62 (d, *J* = 6.4 Hz, 2H), 1.96 (s, 3H). ^{13}C NMR (101 MHz, DMSO-d_6) δ 170.93, 157.07, 144.38, 141.27, 128.19, 127.63, 125.79, 120.67, 66.27, 64.49, 47.14, 43.81, 21.28.

benzyl 1-((2-((((9H-fluoren-9-yl) methoxy) carbonyl) amino) acetamido) methoxy) cyclobutane-1-carboxylate (**S3**): benzyl 1-hydroxycyclobutane-1-carboxylate (1.4 g, 6.8 mmol) was added to 30 mL THF/DCM solution of **S2** (600 mg, 1.6 mmol) and Pyridinium p-Toluenesulfonate (80 mg, 0.32 mmol) in the flask. The mixture was heated to reflux (50 °C) overnight under a nitrogen atmosphere. After TLC monitoring, the mixture was concentrated under a vacuum. The residue was diluted with water (100 mL) and extracted with ethyl acetate (100 mL × 3), washed with saturated brine (50 mL × 2), and dried over anhydrous sodium sulfate. The crude product was purified by column chromatography to afford the product as the pale-yellow solid (250 mg, yield: 30.4%). ESI-MS: m/z = 537.2 $[M+Na]^+$. ^{1}H NMR (400 MHz, Chloroform-*d*) δ 7.73 (dd, *J* = 9.6, 5.5 Hz, 2H), 7.53 (d, *J* = 7.6 Hz, 2H), 7.42 – 7.31 (m, 8H), 7.30 – 7.26 (m, 1H), 5.26 (s, 2H), 4.67 (dd, *J* = 42.0, 10.6 Hz, 2H), 4.46 – 4.33 (m, 2H), 4.19 (dq, *J* = 21.9, 6.7, 6.0 Hz, 1H), 3.90 – 3.71 (m, 2H), 2.51 (dddd, *J* = 13.2, 7.0, 3.4, 1.6 Hz, 2H), 2.39 – 2.27 (m, 2H), 1.98 – 1.80 (m, 2H). ^{13}C NMR (101 MHz, Chloroform-d) δ 176.29, 171.03, 143.73, 141.38, 135.48, 128.80, 128.60, 128.12, 127.87, 127.20, 125.10, 120.11, 74.46, 67.50, 47.12, 44.69, 34.75, 12.98.

benzyl 1-((2-aminoacetamido)methoxy)cyclobutane-1-carboxylate (**S4**): Piperidine (160 mg, 1.87 mmol) was added to 30 mL DMF solution of **S3** (490 mg, 0.95 mmol) in the flask. After TLC and LC-MS monitoring, the mixture was concentrated under a vacuum. The residue was purified by column chromatography to afford the product as the transparent yellowish solid (200 mg, yield: 72.1%). ESI-MS: m/z = 293.1 $[M+H]^+$; 315.1 $[M+Na]^+$.

benzyl (S)-1-((11-benzyl-3,6,9,12,15-pentaoxo-1-phenyl-2-oxa-4,7,10,13,16-pentaazaheptadecan-17-yl)oxy)cyclobutane-1-carboxylate (**S5**): Z-Gly-Gly-Phe-OH (314 mg,

0.76 mmol), HOBT (123 mg, 0.91 mmol), EDC·HCl (174 mg, 0.91 mmol), and DIPEA (150 μL, 0.87 mmol) was added to DMF in the flask, stirred at room temperature for 30min, and then added the DMF solution of **S4** (200 mg, 0.68 mmol) and DIPEA (120 μL, 0.70 mmol) into the flask, react at room temperature for another 4 h. After TLC and LC-MS monitoring, the mixture was concentrated under a vacuum. The residue was purified by column chromatography to afford the product as the colorless solid (160 mg, yield: 34.2%). ESI-MS: m/z = 710.3 $[M+Na]^+$.

(S)-1-((7-benzyl-20-(2,5-dioxo-2,5-dihydro-1H-pyrrol-1-yl)-3,6,9,12,15-pentaoxo-2,5,8,11,14-pentaazaicosyl) oxy) cyclobutane-1-carboxylic acid (**S6**): **S5** (160 mg, 0.23 mmol), dissolved in 5:95 deionized water: methanol (15 mL) to which was added 10% palladium on carbon (25 mg). The mixture was hydrogenated and stirred for 3 h, then vacuum filtered through celite filter aid, and the solvent was removed from the filtrate by rotary evaporation under vacuum to give 70 mg of the desired product as a thick oil (65% yield). The crude product was dissolved in anhydrous DMF (2 mL), to which was added DIPEA (0.1 mL, 0.58 mmol) and 6-Maleimidohexanoic acid N-hydroxysuccinimide ester (101 mg, 0.33 mmol). The reaction was stirred for 2h and then concentrated under a vacuum. The residue was purified by column chromatography to afford the product as the colorless solid (35 mg, yield: 35.7%). ESI-MS: m/z = 679.3 $[M+Na]^+$.

1-(((S)-7-benzyl-20-(2,5-dioxo-2,5-dihydro-1H-pyrrol-1-yl)-3,6,9,12,15-pentaoxo-2,5,8,11,14-pentaazaicosyl)oxy)-N-((1S,9S)-9-ethyl-5-fluoro-9-hydroxy-4-methyl-10,13-dioxo-2,3,9,10,13,15-hexahydro-1H,12H-benzo[de]pyrano[3',4':6,7]indolizino[1,2-b]quinolin-1-yl)cyclobutane-1-carboxamide (**MC-GGFG-Ed9**): **S6** (35 mg, 0.054 mmol), HOBT (9 mg, 0.065 mmol), EDC·HCl (13 mg, 0.065 mmol), and DIPEA (15 μL, 0.085 mmol) was added to DMF in the flask, stirred at room temperature for 30min, and then added the DMF solution of exatecan mesylate (24 mg, 0.044 mmol) and DIPEA (15 μL, 0.085 mmol) into the flask, react at room temperature for another 4h. After TLC monitoring, the mixture was concentrated under a vacuum. The residue was purified by column chromatography to afford the product as the pale-yellow solid (15 mg, yield: 31.8%). ESI-MS: m/z = 1074.4 $[M+H]^+$; 1096.4 $[M+Na]^+$. ^{1}H NMR (400 MHz, DMSO-d_6) δ 8.72 (t, *J* = 6.5 Hz, 1H), 8.64 (t, *J* = 8.9 Hz, 1H), 8.16 – 7.96 (m, 3H), 7.73 – 7.59 (m, 2H), 7.25 (s, 1H), 7.19 – 7.15 (m, 2H), 7.11 (dd, *J* = 7.6, 1.4 Hz, 3H), 6.95 (s, 2H), 6.48 (s, 1H), 5.57 (q, *J* = 7.2 Hz, 1H), 5.36 (s, 2H), 5.08 (s, 2H), 4.54 – 4.47 (m, 1H), 4.45 – 4.32 (m, 2H), 3.62 (p, *J* = 6.0 Hz, 5H), 3.53 – 3.45 (m, 1H), 3.29 (s, 2H), 3.22 (d, *J* = 17.0 Hz, 1H), 3.12 – 2.98 (m, 1H), 2.98 – 2.86 (m, 1H), 2.74 – 2.64 (m,

1H), 2.32 – 2.28 (m, 3H), 2.26 – 2.17 (m, 4H), 2.05 (td, *J* = 7.5, 4.3 Hz, 2H), 2.00 – 1.91 (m, 1H), 1.82 (h, *J* = 7.4 Hz, 4H), 1.66 – 1.54 (m, 1H), 1.42 (q, *J* = 6.6, 5.9 Hz, 5H), 1.33 – 1.29 (m, 1H), 0.84 – 0.81 (m, 3H). HRMS (ESI) for $C_{55}H_{61}FN_9O_{13}$ $[M+H]^+$, calcd: 1074.4373, found: 1074.4358.

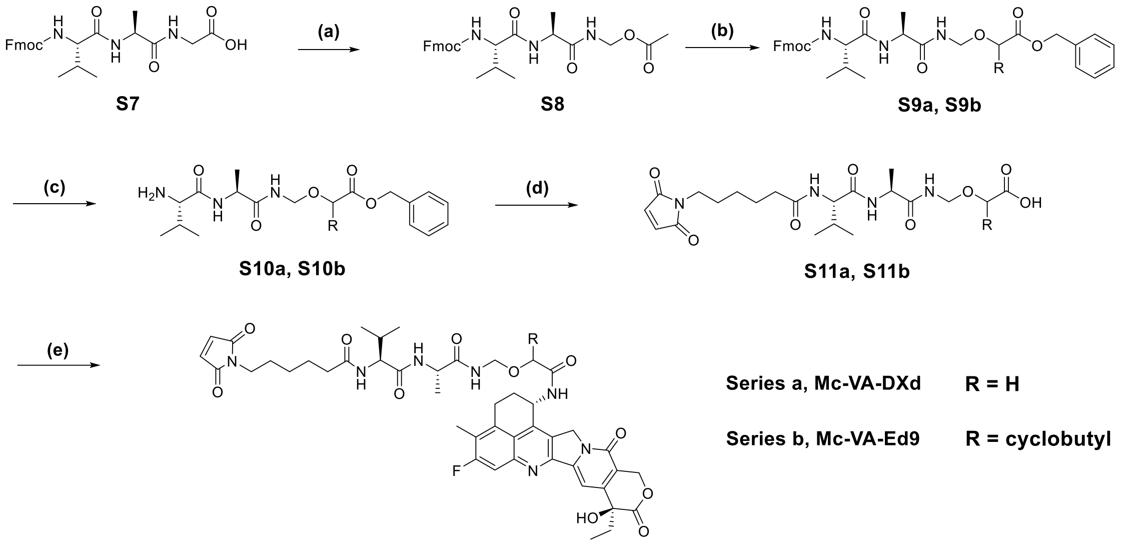

Synthesis of **MC-VA-DXd** and **MC-VA-Ed9**: (a) $Pb(OAc)_4$, pyridine, THF/PhMe, reflux; (b) benzyl 2-hydroxyacetate, PPTS, DCM/THF, reflux; (c) 20% piperidine in DMF, room temp; (d) 1) Pd/C, H_2, MeOH, room temp; 2) Maleimide-NHS, DIPEA, DMF, room temp; (e) exatecan mesylate, EDC·HCl, HOBT, DIPEA, DMF, room temp.

(5S,8S)-1-(9H-fluoren-9-yl)-5-isopropyl-8-methyl-3,6,9-trioxo-2-oxa-4,7,10-triazaundecan-11-yl acetate (**S8**): Fmoc-Val-Ala-Gly-OH (2.3 g, 5 mmol), $Pb(OAc)_4$ (3.1 g, 7 mmol), pyridine (800 μL, 7 mmol), 30 mL tetrahydrofuran and 10 mL toluene was added to a 100mL flask. The mixture was heated to reflux (85°C) for 2.5h under a nitrogen atmosphere. After filtration and concentration, the residue was purified by column chromatography to afford the product as the white solid (500 mg, yield: 20.8%). ESI-MS: m/z = 504.2 $[M+Na]^+$. ^1H NMR (400 MHz, Chloroform-*d*) δ 7.75 (d, *J* = 7.5 Hz, 2H), 7.58 (d, *J* = 7.6 Hz, 3H), 7.38 (t, *J* = 7.6 Hz, 2H), 7.29 (ddd, *J* = 7.7, 4.5, 1.7 Hz, 2H), 5.54 (d, *J* = 8.5 Hz, 1H), 5.22 (d, *J* = 7.1 Hz, 1H), 4.53 (dt, *J* = 12.7, 6.9 Hz, 1H), 4.45 (dd, *J* = 10.5, 7.1 Hz, 1H), 4.37 (t, *J* = 8.7 Hz, 1H), 4.20 (t, *J* = 6.6 Hz, 1H), 4.01 (t, *J* = 7.5 Hz, 1H), 2.05 (d, *J* = 25.8 Hz, 4H), 1.36 (d, *J* = 7.1 Hz, 3H), 0.99 – 0.86 (m, 6H).

benzyl (5S,8S)-1-(9H-fluoren-9-yl)-5-isopropyl-8-methyl-3,6,9-trioxo-2,12-dioxa-4,7,10-triazatetradecan-14-oate (**S9a**): benzyl 2-hydroxyacetate (700 mg, 4.2 mmol) was added to 30

mL THF/DCM (2:1) solution of **S8** (200 mg, 0.42 mmol) and Pyridinium p-Toluenesulfonate (15 mg, 0.06 mmol) in the flask. The mixture was heated to reflux (50 °C) overnight under a nitrogen atmosphere. After TLC monitoring, the mixture was concentrated under a vacuum. The residue was diluted with water (30 mL) and extracted with ethyl acetate (50 mL × 3), washed with saturated brine (50 mL × 2), and dried over anhydrous sodium sulfate. The crude product was purified by column chromatography to afford the product as the colorless solid (91 mg, yield: 36.9%). ESI-MS: m/z = 610.3 $[M+Na]^+$. ^{1}H NMR (400 MHz, DMSO-d_6) δ 8.71 (t, *J* = 6.8 Hz, 1H), 8.08 (d, *J* = 7.0 Hz, 1H), 7.85 (d, *J* = 7.5 Hz, 2H), 7.71 (dd, *J* = 7.3, 5.5 Hz, 2H), 7.42 – 7.35 (m, 3H), 7.33 (d, *J* = 3.6 Hz, 4H), 7.31 – 7.25 (m, 3H), 5.10 (s, 2H), 4.63 – 4.56 (m, 2H), 4.29 – 4.15 (m, 4H), 4.10 (s, 2H), 3.86 (dd, *J* = 8.9, 7.0 Hz, 1H), 2.01 – 1.88 (m, 1H), 1.18 (d, *J* = 7.1 Hz, 3H), 0.81 (dd, *J* = 10.4, 6.8 Hz, 6H). ^{13}C NMR (101 MHz, DMSO-d_6) δ 174.08, 171.54, 170.44, 156.69, 144.43, 141.25, 136.36, 128.98, 128.65, 128.55, 128.19, 127.61, 125.93, 120.65, 69.45, 66.14, 64.67, 60.41, 48.93, 47.20, 30.91, 19.71, 18.72, 18.36.

benzyl 1-(((5S,8S)-1-(9H-fluoren-9-yl)-5-isopropyl-8-methyl-3,6,9-trioxo-2-oxa-4,7,10-triazaundecan-11-yl)oxy)cyclobutane-1-carboxylate (**S9b**): benzyl 1-hydroxycyclobutane-1-carboxylate (840 mg, 4 mmol) was added to 30 mL THF/DCM solution of **S8** (210 mg, 0.44 mmol) and Pyridinium p-Toluenesulfonate (20 mg, 0.08 mmol) in the flask. The mixture was heated to reflux (50 °C) overnight under a nitrogen atmosphere. After TLC monitoring, the mixture was concentrated under a vacuum. The residue was diluted with water (30 mL) and extracted with ethyl acetate (50 mL × 3), washed with saturated brine (50 mL × 2), and dried over anhydrous sodium sulfate. The crude product was purified by column chromatography to afford the product as the colorless solid (85 mg, yield: 30.8%). ESI-MS: m/z = 650.3 $[M+Na]^+$.

benzyl 2-(((S)-2-((S)-2-amino-3-methylbutanamido)propanamido)methoxy)acetate (**S10a**): Piperidine (20 mg, 0.23 mmol) was added to 5 mL DMF solution of **S9a** (70 mg, 0.12 mmol) in the flask. After TLC and LC-MS monitoring, the mixture was concentrated under a vacuum. The residue was purified by column chromatography to afford the product as the colorless solid (38 mg, yield: 86.7%). ESI-MS: m/z = 366.2 $[M+H]^+$; 388.2 $[M+Na]^+$.

benzyl 1-(((S)-2-((S)-2-amino-3-methylbutanamido)propanamido)methoxy)cyclobutane-1-carboxylate (**S10b**): Compound **S10b** was synthesized from **S9b** with the experimental procedure described for **S10a**. The yield was 88.9%, as a pale-yellow solid. ESI-MS: m/z = 406.2 $[M+H]^+$; 428.2 $[M+Na]^+$.

(7S,10S)-17-(2,5-dioxo-2,5-dihydro-1H-pyrrol-1-yl)-10-isopropyl-7-methyl-6,9,12-trioxo-3-oxa-5,8,11-triazaheptadecanoic acid (**S11a**): **S10a** (50 mg, 0.14 mmol), dissolved in 5:95 deionized water: methanol (25 mL) to which was added 10% palladium on carbon (10 mg). The mixture was hydrogenated and stirred for 3 h, then vacuum filtered through celite filter aid, and the solvent was removed from the filtrate by rotary evaporation under vacuum to give 35 mg of the desired product as a thick oil (93.9% yield). The crude product was dissolved in anhydrous DMF (4 mL), to which was added DIPEA (35 μL, 0.2 mmol) and 6-Maleimidohexanoic acid N-hydroxysuccinimide ester (62 mg, 0.2 mmol). The reaction was stirred for 2h and then concentrated under a vacuum. The residue was purified by column chromatography to afford the product as the white powder (52 mg, yield: 84.6%). ESI-MS: m/z = 469.2 $[M+H]^+$; 491.2 $[M+Na]^+$.

1-(((S)-2-((S)-2-(6-(2,5-dioxo-2,5-dihydro-1H-pyrrol-1-yl)hexanamido)-3-methylbutanamido) propanamido)methoxy)cyclobutane-1-carboxylic acid (**S11b**): Compound **S11b** was synthesized from **S10b** with the experimental procedure described for **S11a**. The yield was 78.1%, as a pale yellow-white powdery solid. ESI-MS: m/z = 509.2 $[M+H]^+$; 531.2 $[M+Na]^+$.

6-(2,5-dioxo-2,5-dihydro-1H-pyrrol-1-yl)-N-((S)-1-(((S)-1-(((2-(((1S,9S)-9-ethyl-5-fluoro-9-hydroxy-4-methyl-10,13-dioxo-2,3,9,10,13,15-hexahydro-1H,12H-naphtho[1,8-ef]pyrano[4',3':4,5] pyrido[2,1-a]isoindol-1-yl)amino)-2-oxoethoxy)methyl)amino)-1-oxopropan-2-yl)amino)-3-methyl-1-oxobutan-2-yl)hexanamide (**MC-VA-DXd**): **S11a** (30 mg, 0.064 mmol), HOBT (10 mg, 0.077 mmol), EDC·HCl (15 mg, 0.077 mmol), and DIPEA (15 μL, 0.085 mmol) was added to DMF in the flask, stirred at room temperature for 30min, and then added the DMF solution of exatecan mesylate (27 mg, 0.051 mmol) and DIPEA (15 μL, 0.085 mmol) into the flask, react at room temperature for another 4h. After TLC monitoring, the mixture was concentrated under a vacuum. The residue was purified by column chromatography to afford the product as the white powder (52 mg, yield: 87.2%). ESI-MS: m/z = 886.4 $[M+H]^+$; 908.4 $[M+Na]^+$. ^{1}H NMR (400 MHz, DMSO-d_6) δ 8.66 (t, *J* = 6.7 Hz, 1H), 8.46 (d, *J* = 8.9 Hz, 1H), 7.99 (d, *J* = 7.1 Hz, 1H), 7.74 (d, *J* = 10.0 Hz, 2H), 7.27 (s, 1H), 6.96 (s, 2H), 6.50 (s, 1H), 5.56 (q, *J* = 6.8, 6.2 Hz, 1H), 5.38 (d, *J* = 2.1 Hz, 2H), 5.14 (s, 2H), 4.66 – 4.53 (m, 2H), 4.16 (q, *J* = 7.1, 6.4 Hz, 1H), 4.03 (dd, *J* = 8.5, 6.8 Hz, 1H), 3.95 (s, 2H), 3.32 (s, 2H), 3.12 (td, *J* = 17.1, 15.9, 8.7 Hz, 2H), 2.35 (d, *J* = 1.9 Hz, 3H), 2.22 – 1.90 (m, 5H), 1.83

(dq, *J* = 14.5, 6.8 Hz, 3H), 1.51 – 1.35 (m, 5H), 1.14 (d, *J* = 7.0 Hz, 3H), 0.84 (d, *J* = 7.3 Hz, 3H), 0.72 (dd, *J* = 9.5, 6.7 Hz, 6H). ^{13}C NMR (101 MHz, DMSO-d_6) δ 174.04, 173.01, 172.89, 171.61, 171.43, 169.66, 163.42, 160.95, 157.24, 152.93, 150.58, 148.54, 148.40, 145.75, 141.08, 137.03, 134.98, 130.19, 126.06, 124.30, 124.10, 122.28, 119.63, 110.51, 110.28, 97.25, 72.89, 70.30, 67.30, 65.75, 58.03, 50.21, 48.80, 45.00, 37.53, 35.39, 30.77, 28.29, 26.30, 25.39, 24.12, 19.65, 18.58, 18.23, 11.56, 11.51, 8.27. HRMS (ESI) for $C_{45}H_{53}FN_7O_{11}$ [M+H]$^+$, calcd: 886.3787, found: 886.3769. $C_{45}H_{52}FN_7NaO_{11}$ [M+Na]$^+$, calcd: 908.3607, found: 908.3593.

1-(((S)-2-((S)-2-(6-(2,5-dioxo-2,5-dihydro-1H-pyrrol-1-yl)hexanamido)-3-methylbutanamido) propanamido)methoxy)-N-((1S,9S)-9-ethyl-5-fluoro-9-hydroxy-4-methyl-10,13-dioxo-2,3,9,10,13,15-hexahydro-1H,12H-naphtho[1,8-ef]pyrano[4',3':4,5]pyrido[2,1-a]isoindol-1-yl)cyclobutane-1-carboxamide (**MC-VA-Ed9**): Compound **MC-VA-Ed9** was synthesized from **S11b** with the experimental procedure described for **MC-VA-DXd**. The yield was 83.3%, as an off-white solid. ESI-MS: m/z = 926.4 [M+H]$^+$; 948.4 [M+Na]$^+$. ^{1}H NMR (400 MHz, DMSO-d_6) δ 8.72 (t, *J* = 6.4 Hz, 1H), 8.62 (d, *J* = 9.0 Hz, 1H), 7.89 (d, *J* = 7.2 Hz, 1H), 7.66 (d, *J* = 2.5 Hz, 1H), 7.63 (d, *J* = 4.9 Hz, 1H), 7.23 (s, 1H), 6.95 (s, 2H), 6.48 (s, 1H), 5.56 (q, *J* = 8.1 Hz, 1H), 5.41 – 5.29 (m, 2H), 4.95 (q, *J* = 18.9 Hz, 2H), 4.43 (ddd, *J* = 31.1, 8.9, 6.6 Hz, 2H), 4.15 (q, *J* = 7.1 Hz, 1H), 3.95 (dd, *J* = 8.6, 6.7 Hz, 1H), 3.30 (s, 1H), 3.20 (d, *J* = 17.0 Hz, 1H), 3.06 (q, *J* = 10.7, 10.1 Hz, 1H), 2.50 (d, *J* = 5.2 Hz, 1H), 2.32 – 2.29 (m, 3H), 2.19 (dq, *J* = 20.4, 9.1 Hz, 5H), 2.01 (tq, *J* = 14.2, 7.0 Hz, 3H), 1.81 (dq, *J* = 13.9, 7.6, 6.6 Hz, 5H), 1.67 (h, *J* = 6.7 Hz, 1H), 1.47 – 1.32 (m, 5H), 1.18 (s, 1H), 1.08 (d, *J* = 7.1 Hz, 3H), 0.81 (t, *J* = 7.3 Hz, 3H), 0.54 (d, *J* = 2.7 Hz, 3H), 0.52 (d, *J* = 2.7 Hz, 3H). ^{13}C NMR (101 MHz, DMSO-d_6) δ 174.32, 173.39, 173.00, 172.72, 171.59, 171.26, 163.29, 160.82, 157.10, 152.84, 150.44, 148.45, 148.30, 145.53, 141.67, 136.94, 136.88, 134.97, 125.39, 124.16, 123.97, 122.31, 119.56, 110.45, 110.22, 97.22, 79.77, 72.88, 66.43, 65.74, 57.75, 50.34, 48.50, 46.48, 37.51, 35.32, 32.27, 30.87, 30.75, 29.96, 28.81, 28.27, 26.27, 25.36, 24.99, 19.32, 18.32, 18.12, 14.15, 11.49, 11.44, 8.24. HRMS (ESI) for $C_{48}H_{57}FN_7O_{11}$ [M+H]$^+$, calcd: 926.4100, found: 926.4086. $C_{48}H_{56}FN_7NaO_{11}$ [M+Na]$^+$, calcd: 948.3920, found: .948.3905.

The spectrum of compound **Ed1**

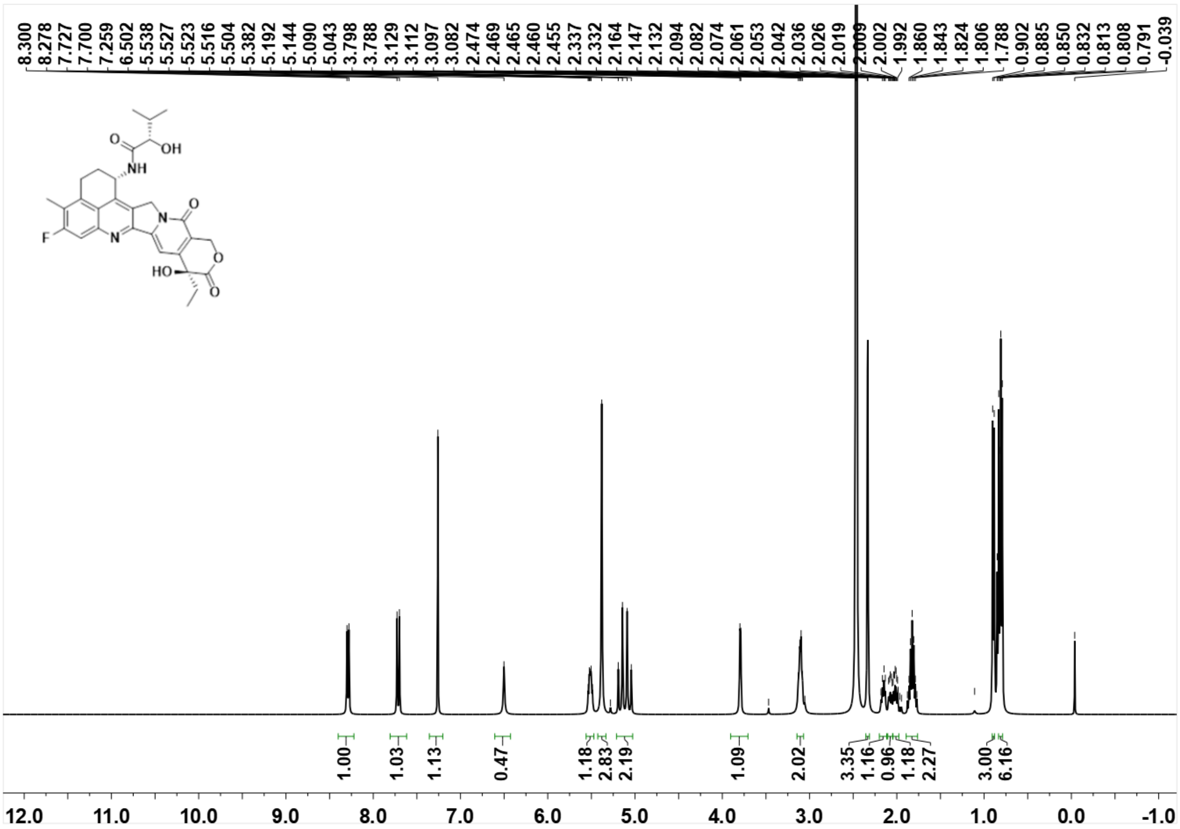

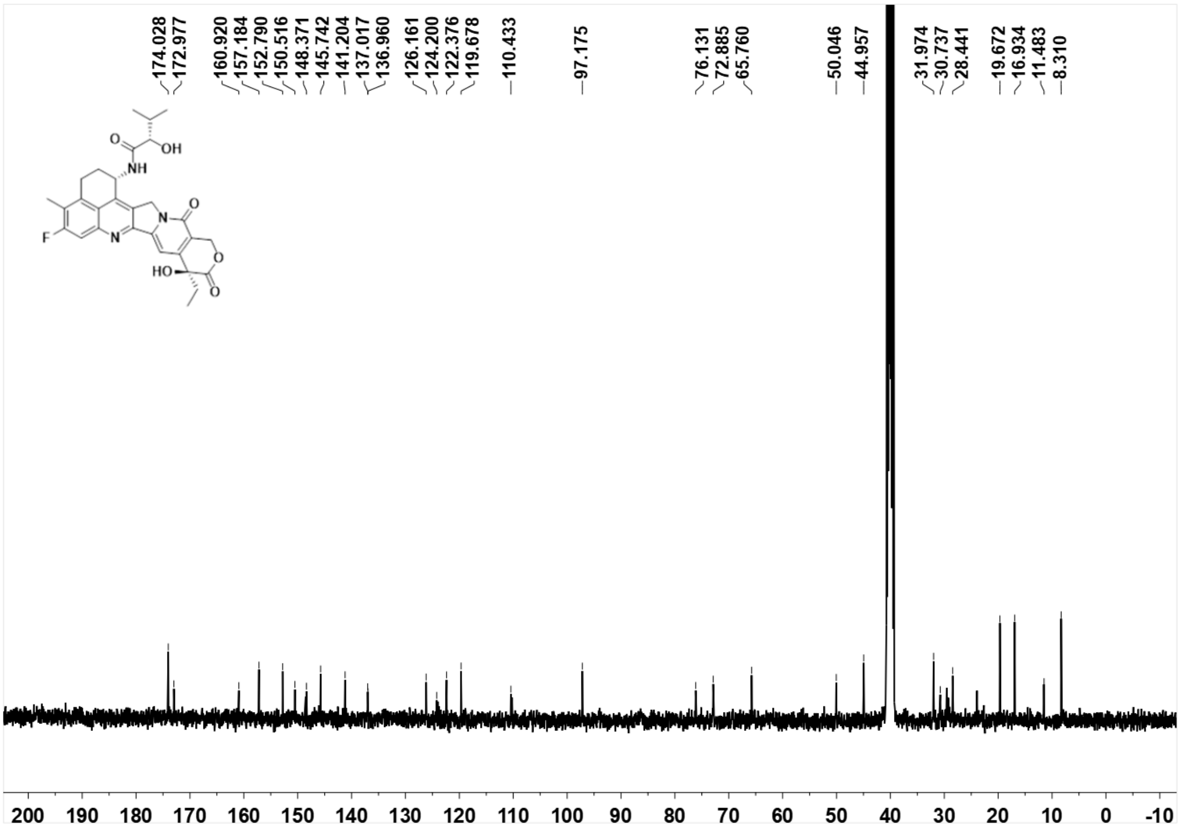

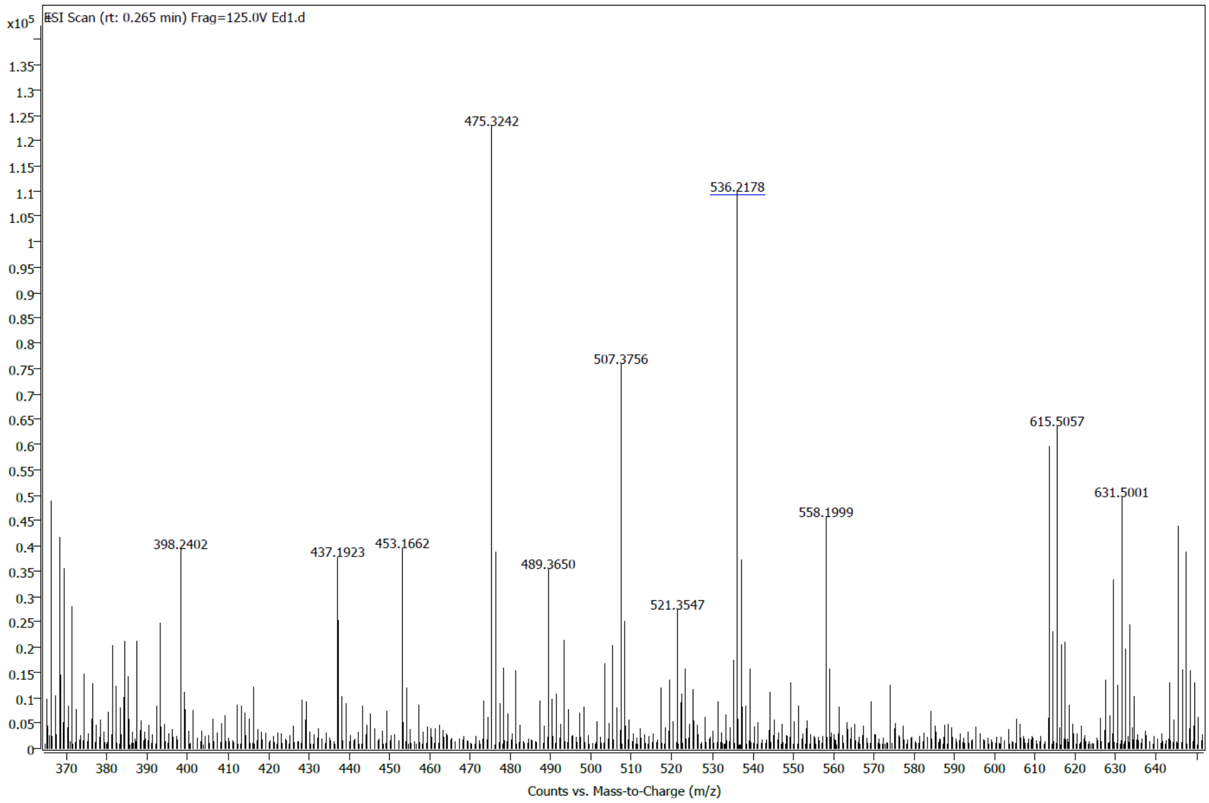

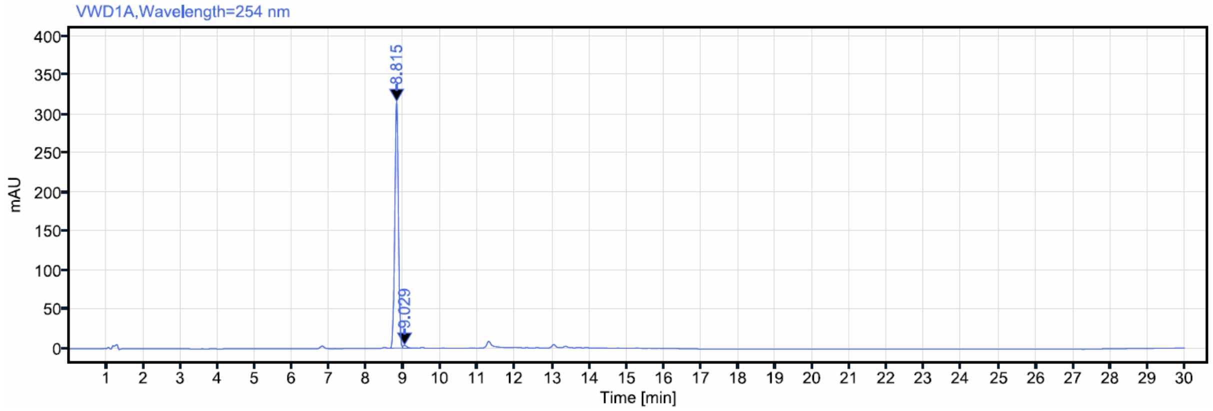

Signal: VWD1A,Wavelength=254 nm

RT [min]	Type	Width [min]	Area	Height	Area%	Symmetry
8.815	VV	0.35	2164.453	316.51	98.982	1.05
9.029	VB	0.21	22.254	3.42	1.018	0.78
		Sum	2186.707			

The spectrum of compound **Ed2**

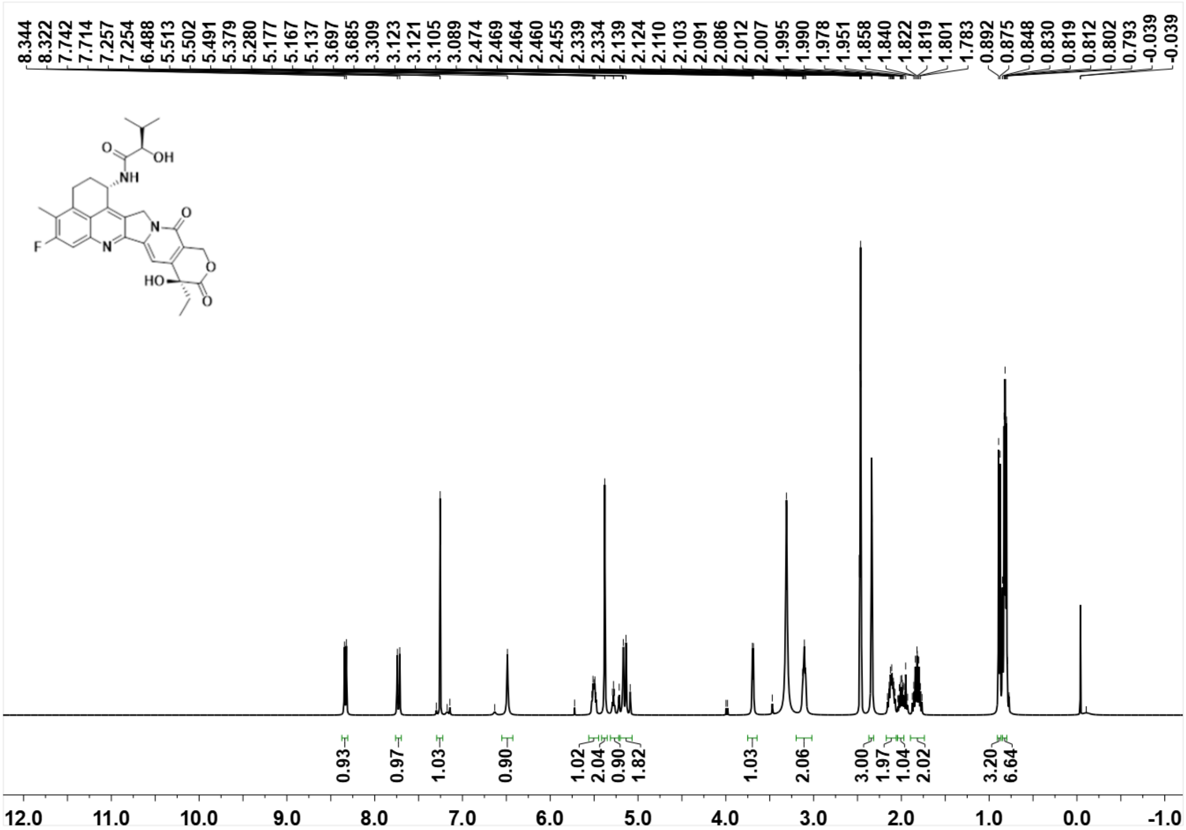

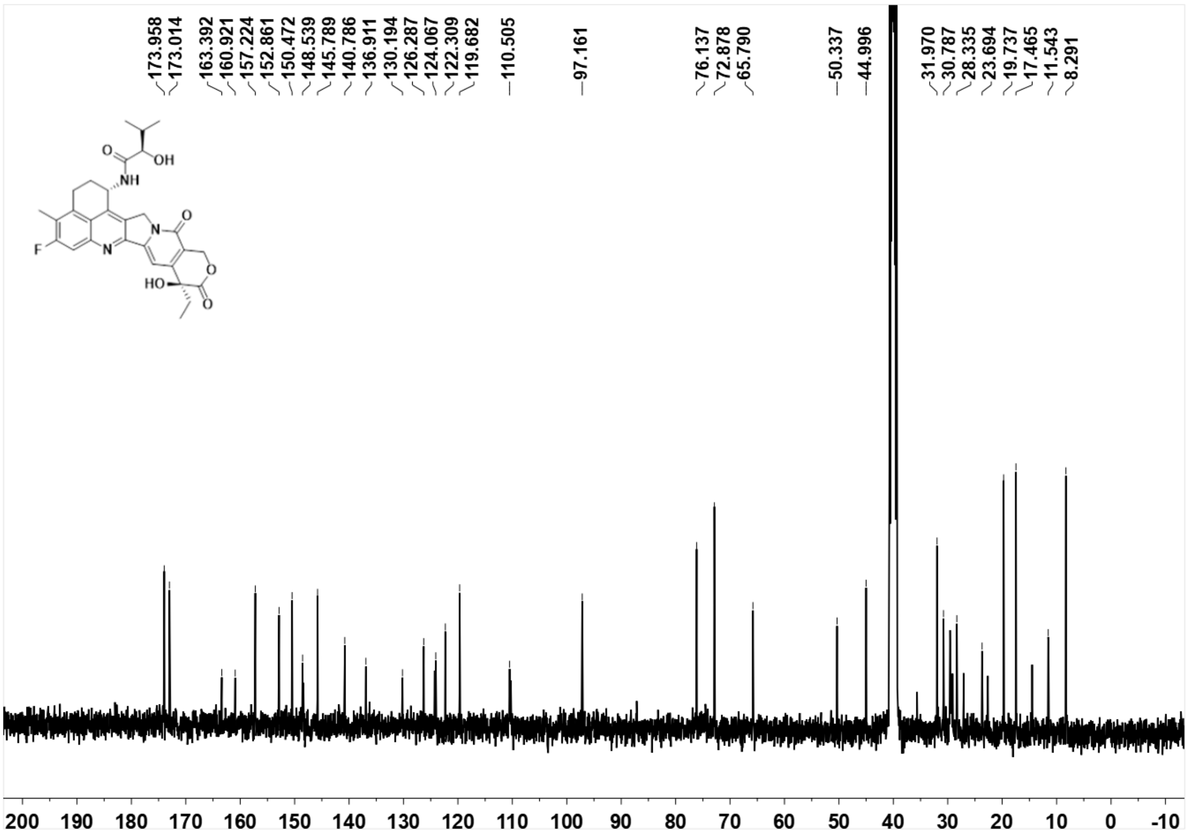

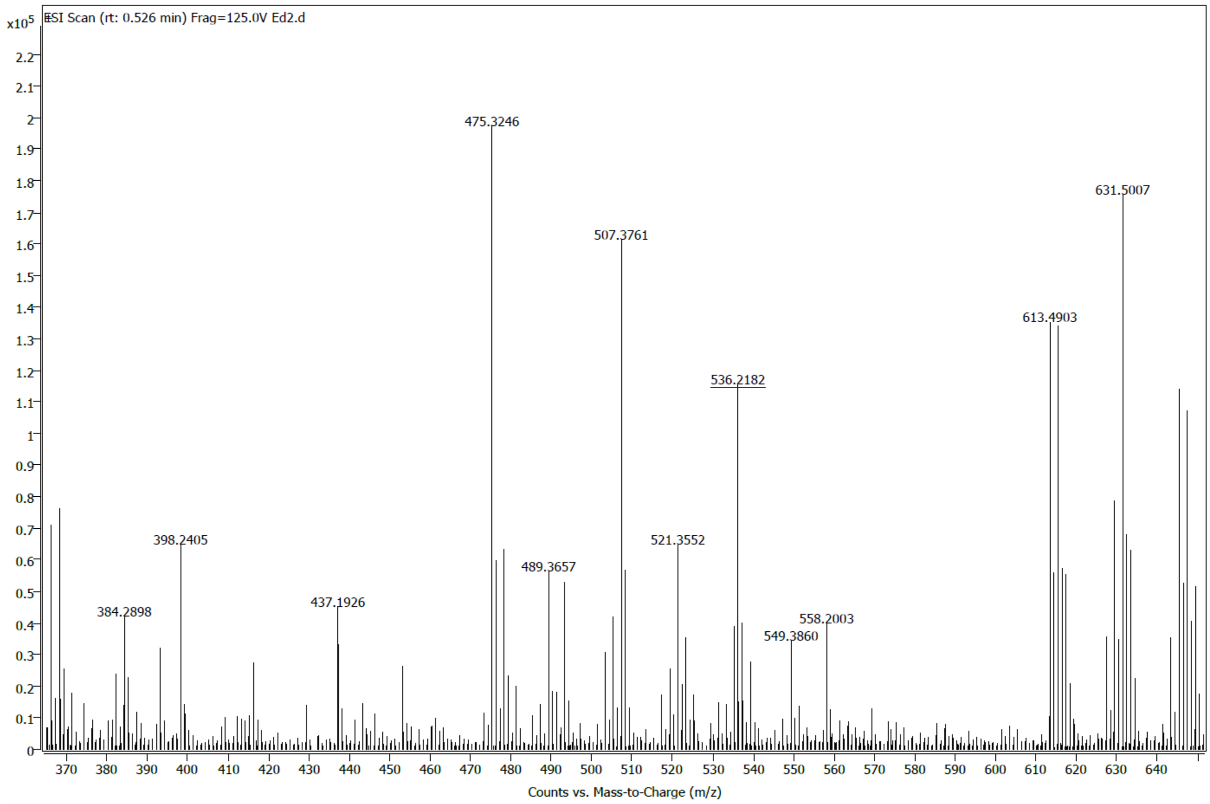

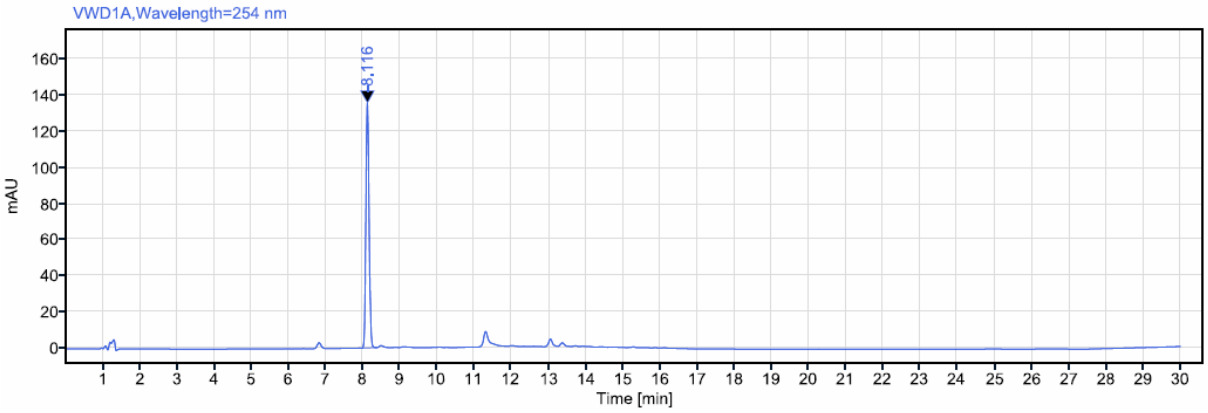

Signal: VWD1A,Wavelength=254 nm

RT [min]	Type	Width [min]	Area	Height	Area%	Symmetry
8.116	BB	0.39	827.013	135.85	100.000	0.88
		Sum	827.013			

The spectrum of compound **Ed3**

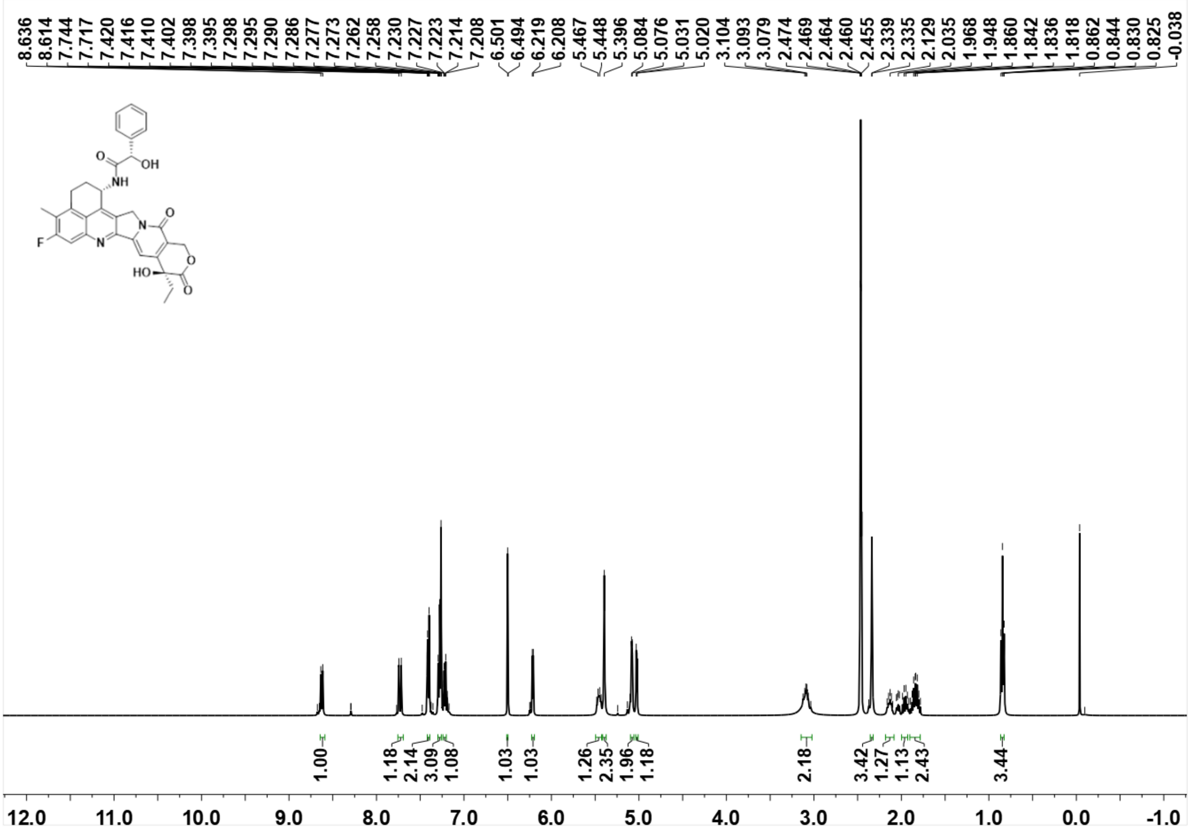

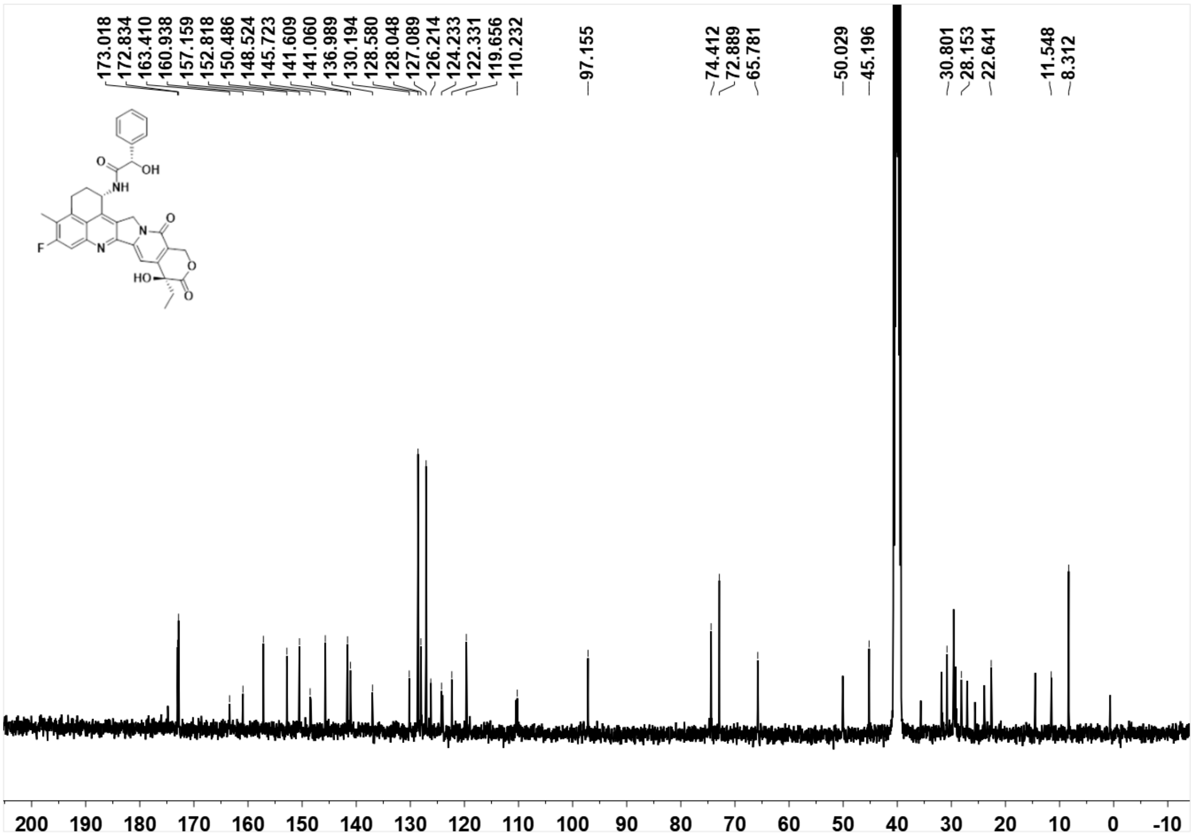

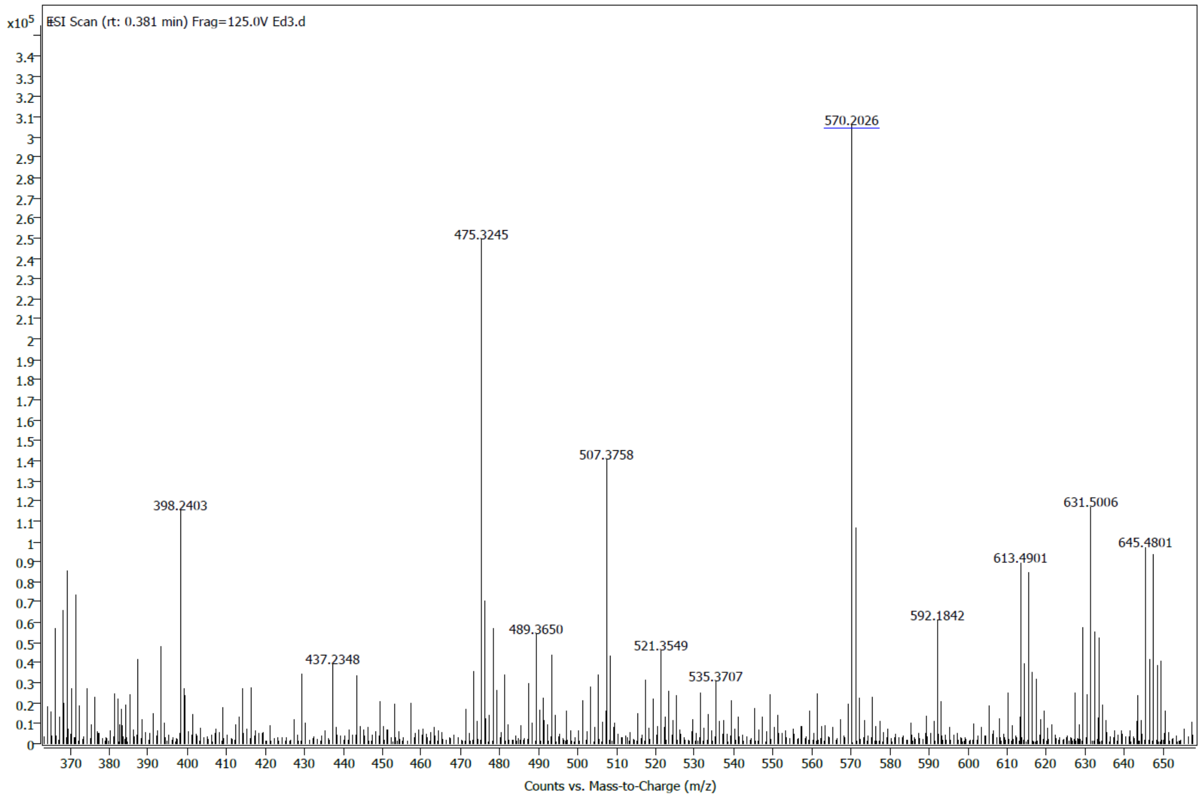

x10^5
ESI Scan (rt: 0.381 min) Frag=125.0V Ed3.d
570.2026
475.3245
507.3758
398.2403
631.5006
645.4801
613.4901
489.3650
592.1842
521.3549
437.2348
535.3707
Counts vs. Mass-to-Charge (m/z)

The spectrum of compound **Ed4**

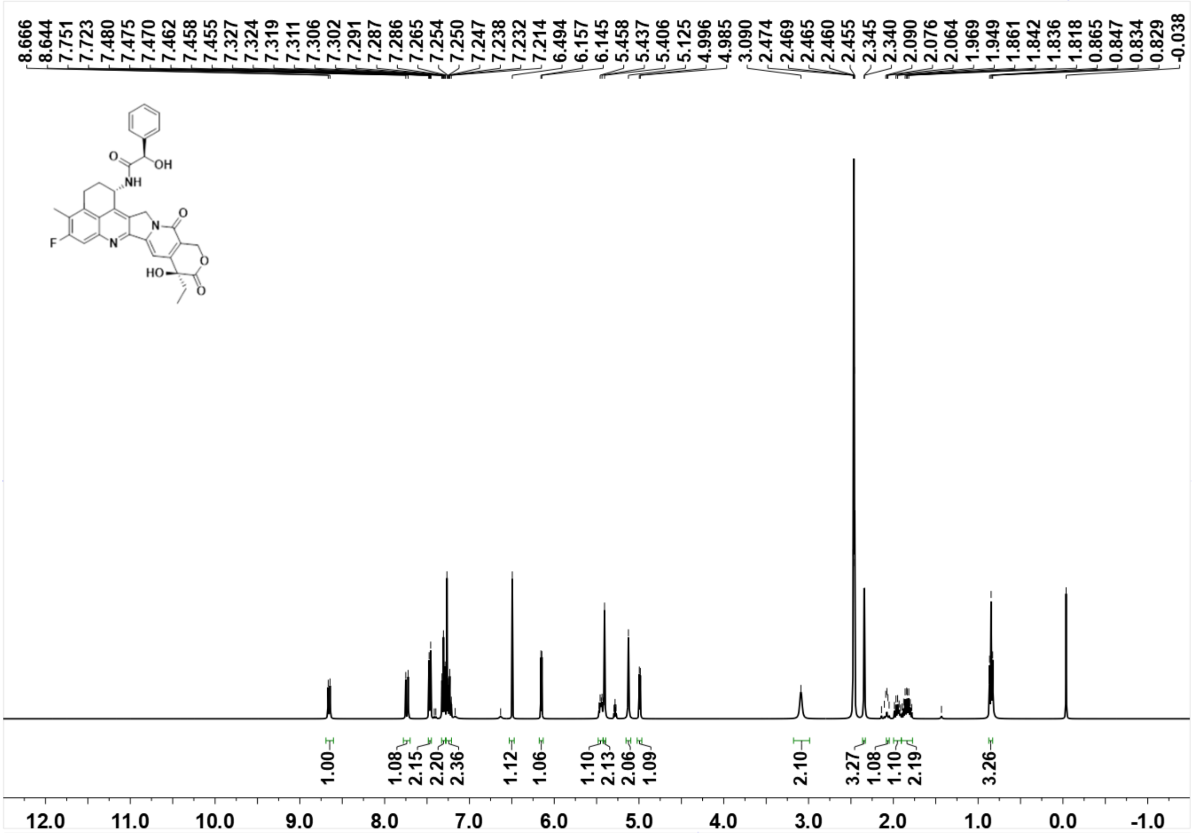

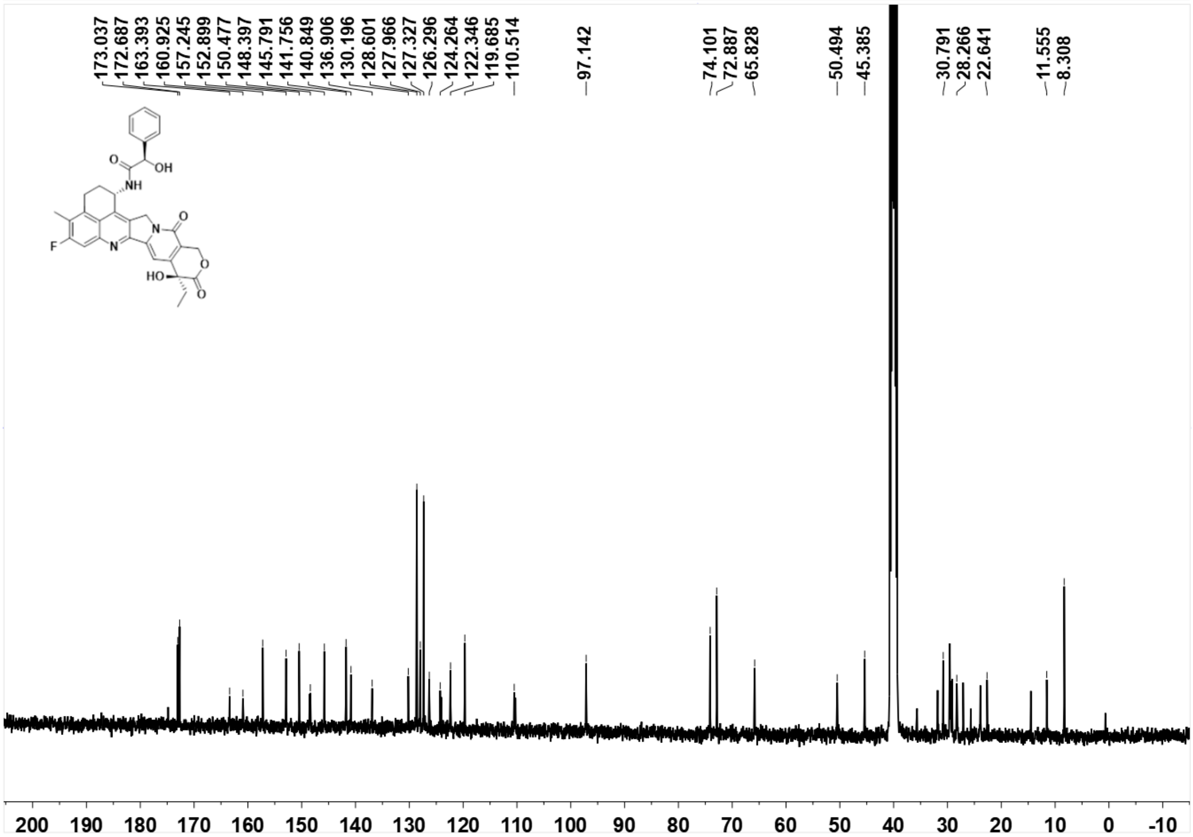

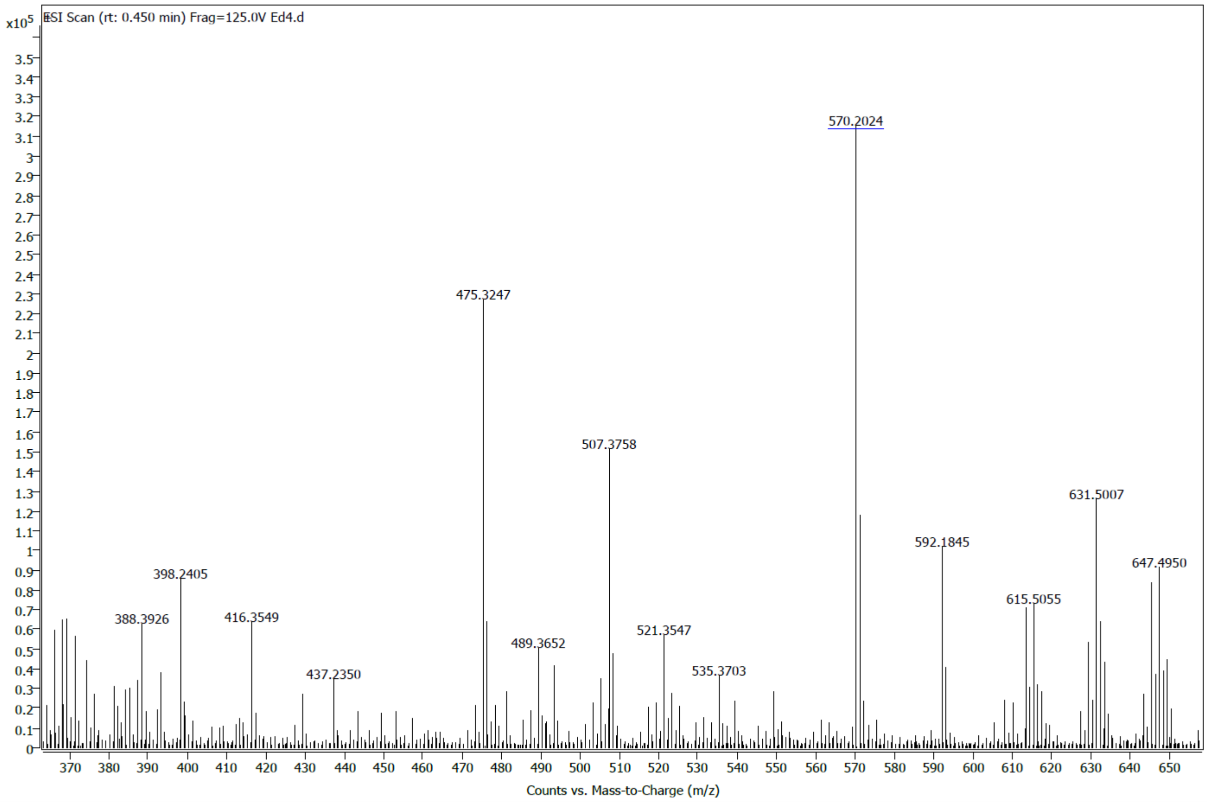

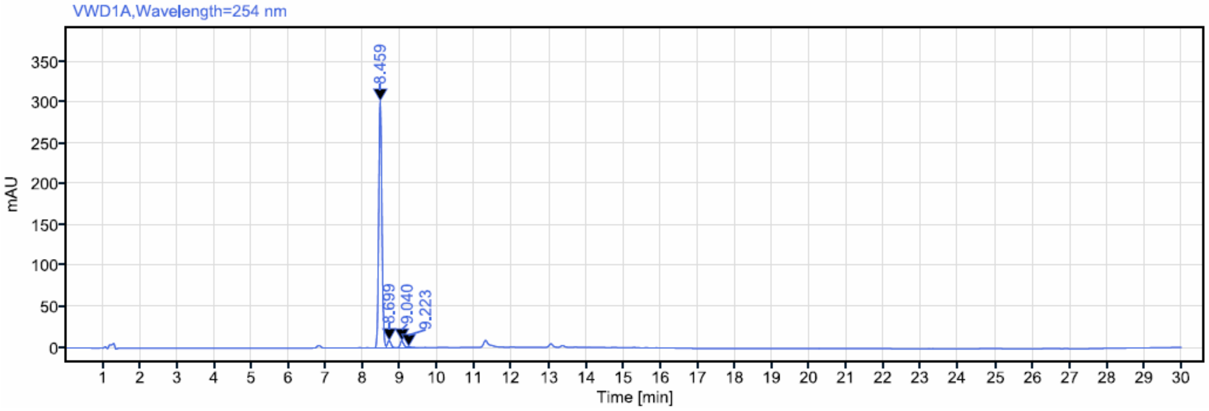

Signal: VWD1A,Wavelength=254 nm

RT [min]	Type	Width [min]	Area	Height	Area%	Symmetry
8.459	BV	0.33	1841.735	301.76	93.815	0.90
8.699	VB	0.26	56.455	8.67	2.876	0.92
9.040	BV	0.28	54.176	8.25	2.760	0.83
9.223	VB	0.30	10.795	1.33	0.550	0.54
		Sum	1963.160			

The spectrum of compound **Ed5**

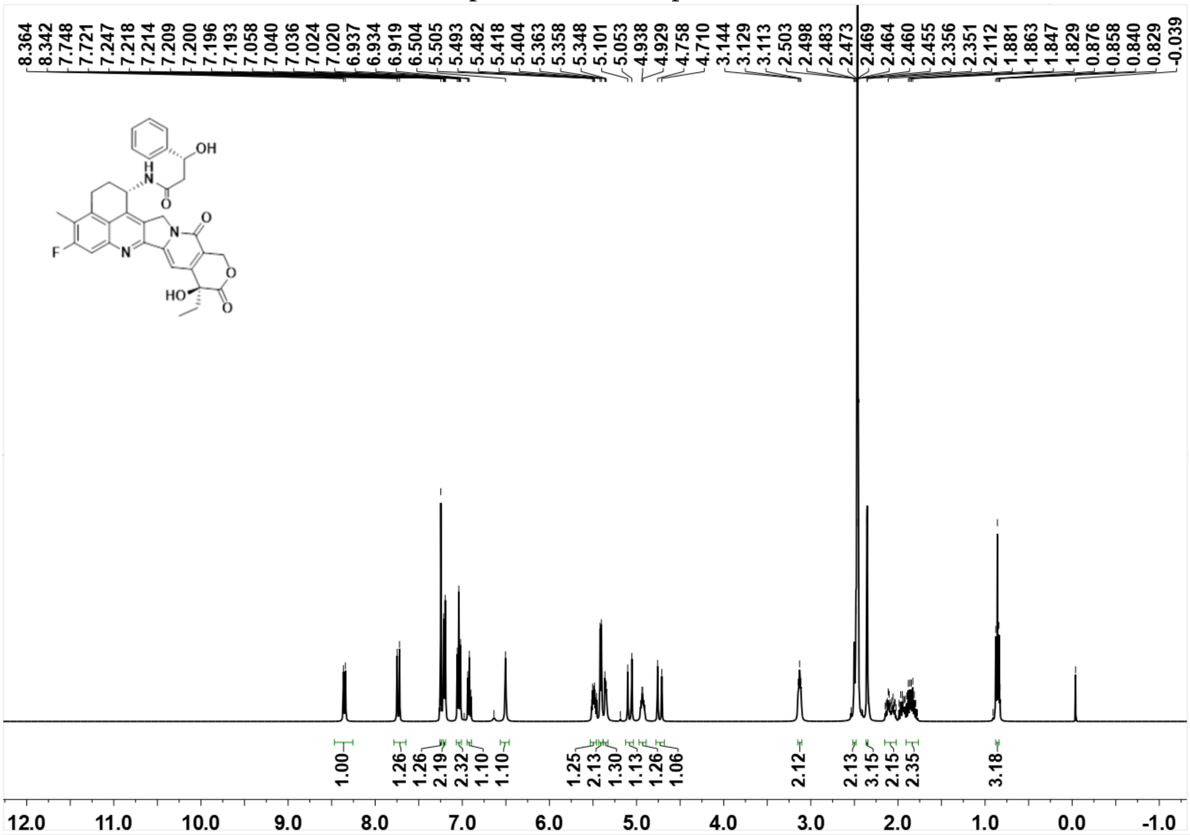

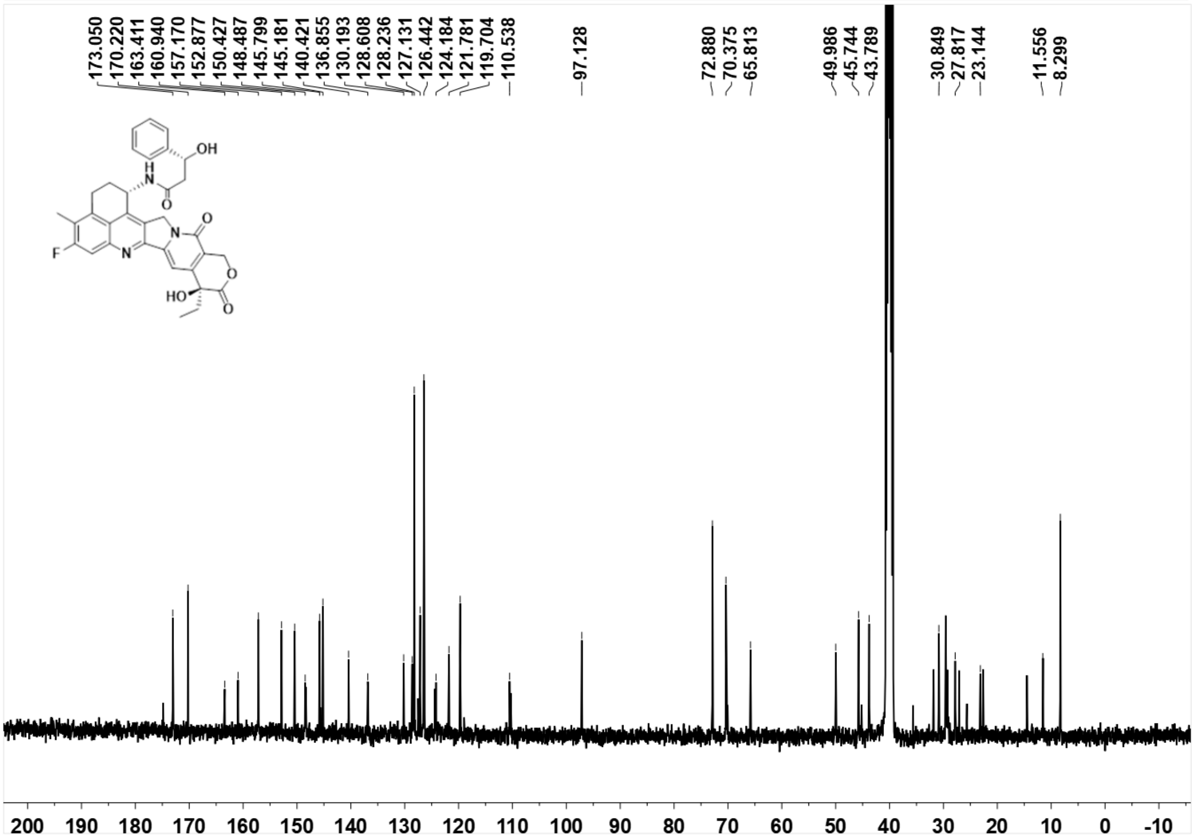

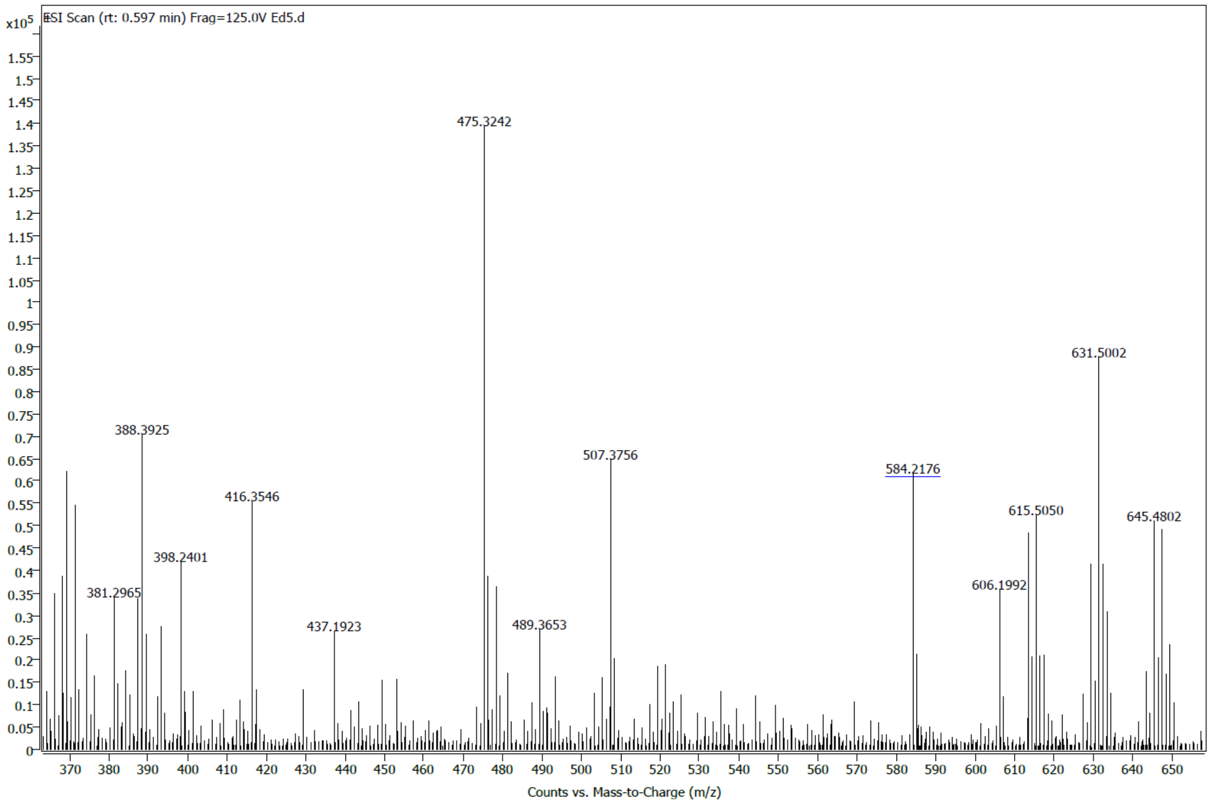

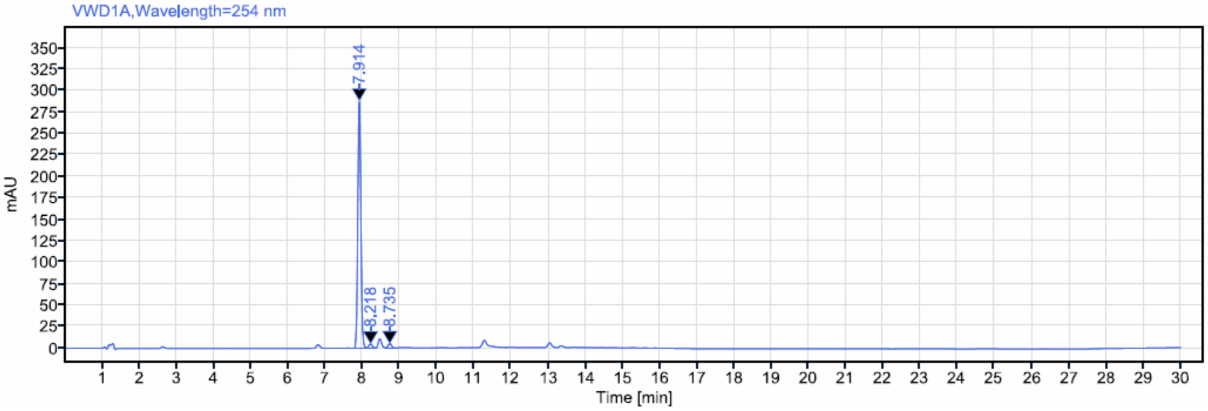

Signal: VWD1A,Wavelength=254 nm

RT [min]	Type	Width [min]	Area	Height	Area%	Symmetry
7.914	BV	0.36	1703.358	288.22	96.603	0.89
8.218	VB	0.23	29.271	4.82	1.660	0.94
8.735	VB	0.26	30.626	4.99	1.737	0.91
		Sum	1763.256			

The spectrum of compound **Ed6**

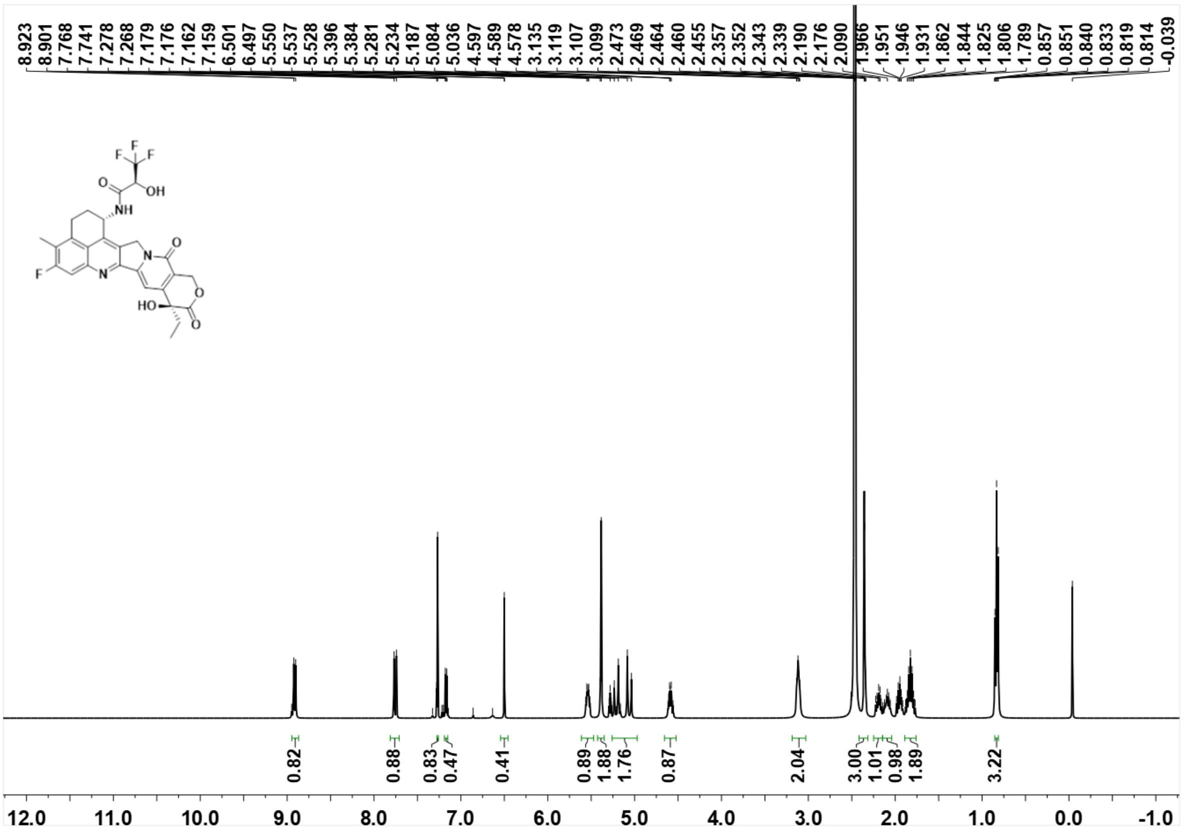

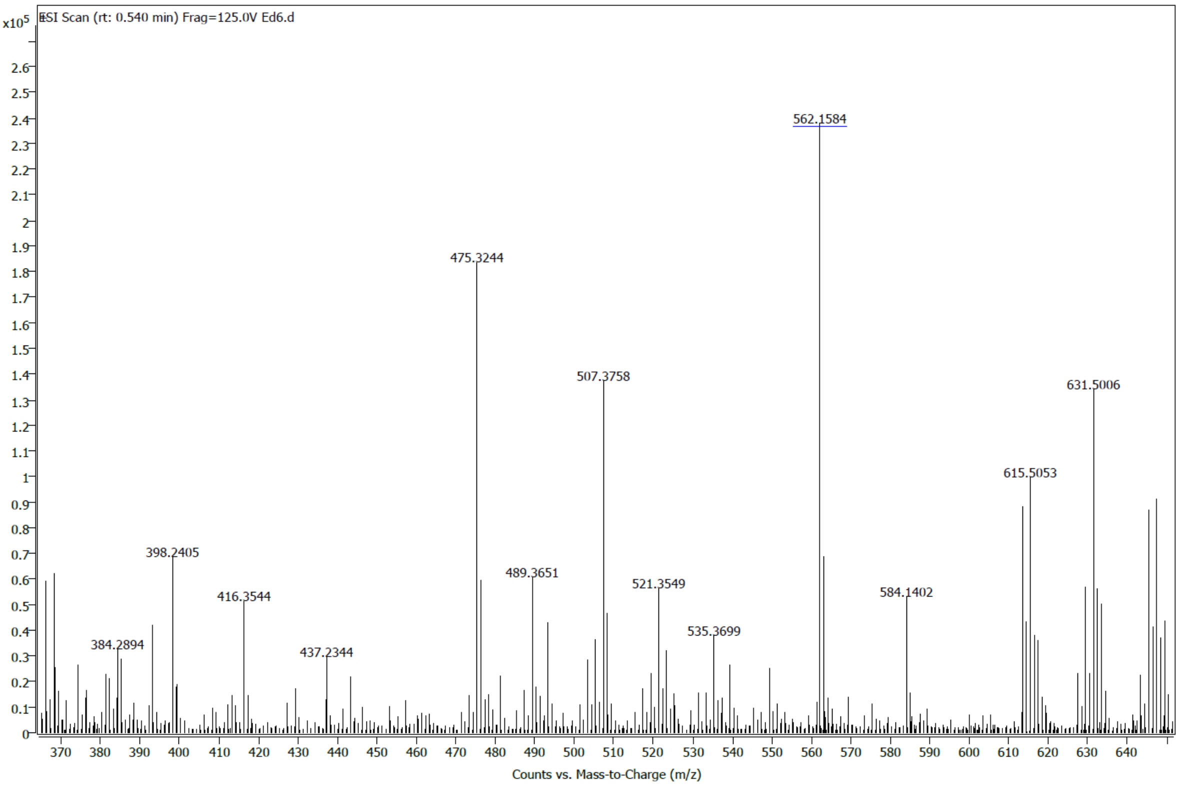

The spectrum of compound **Ed7**

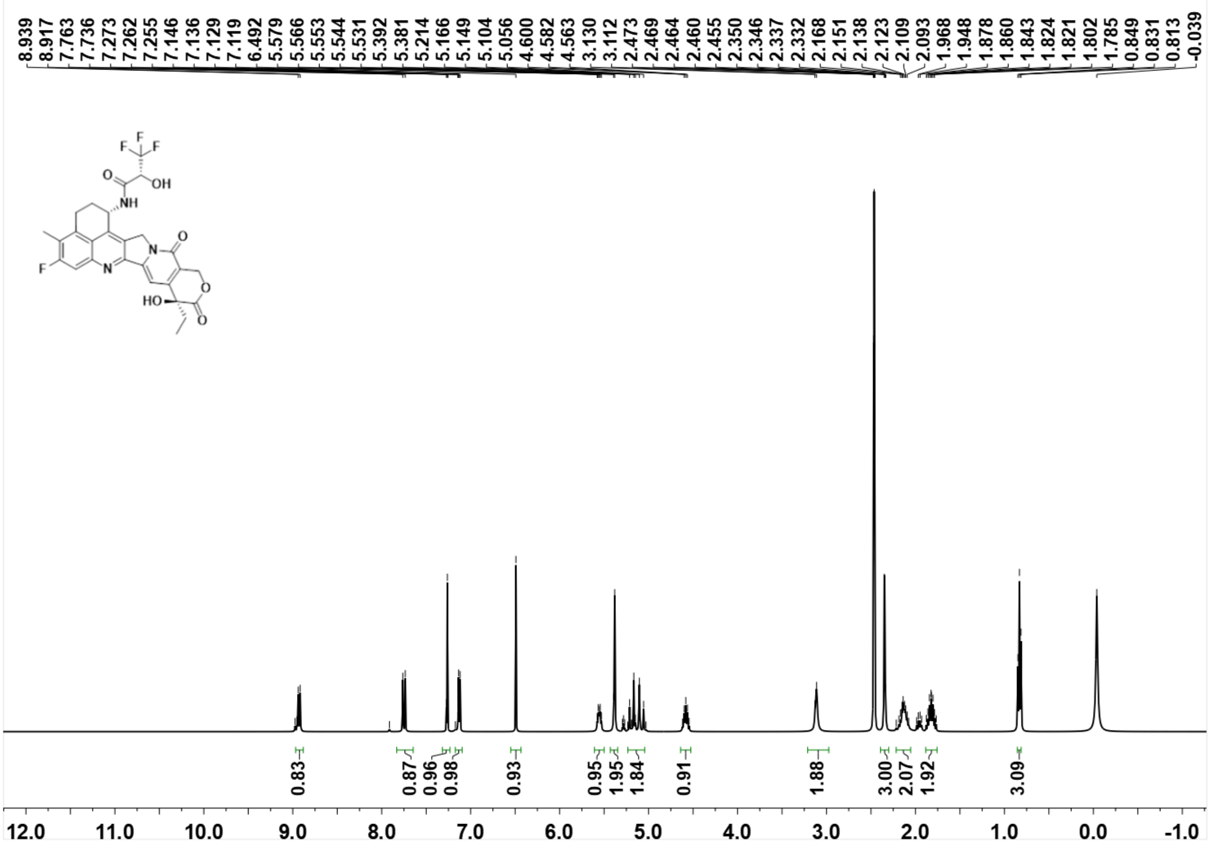

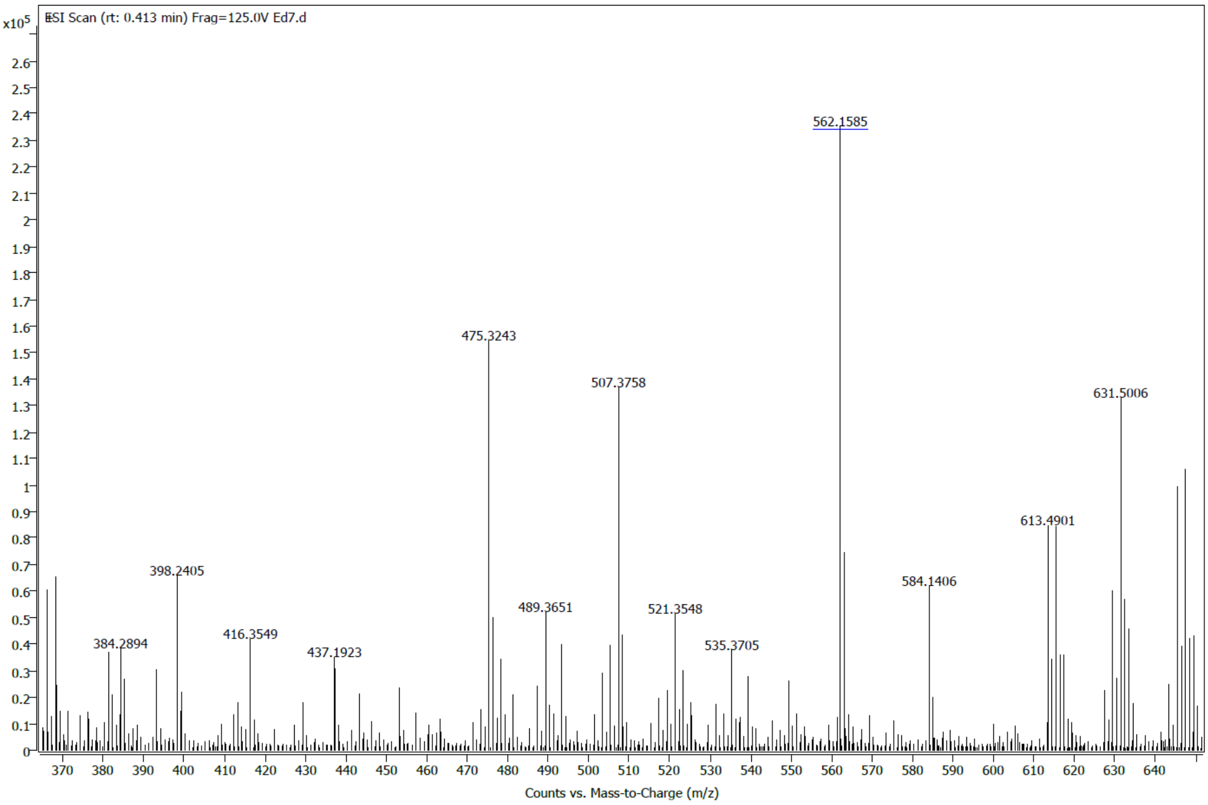

The spectrum of compound **Ed8**

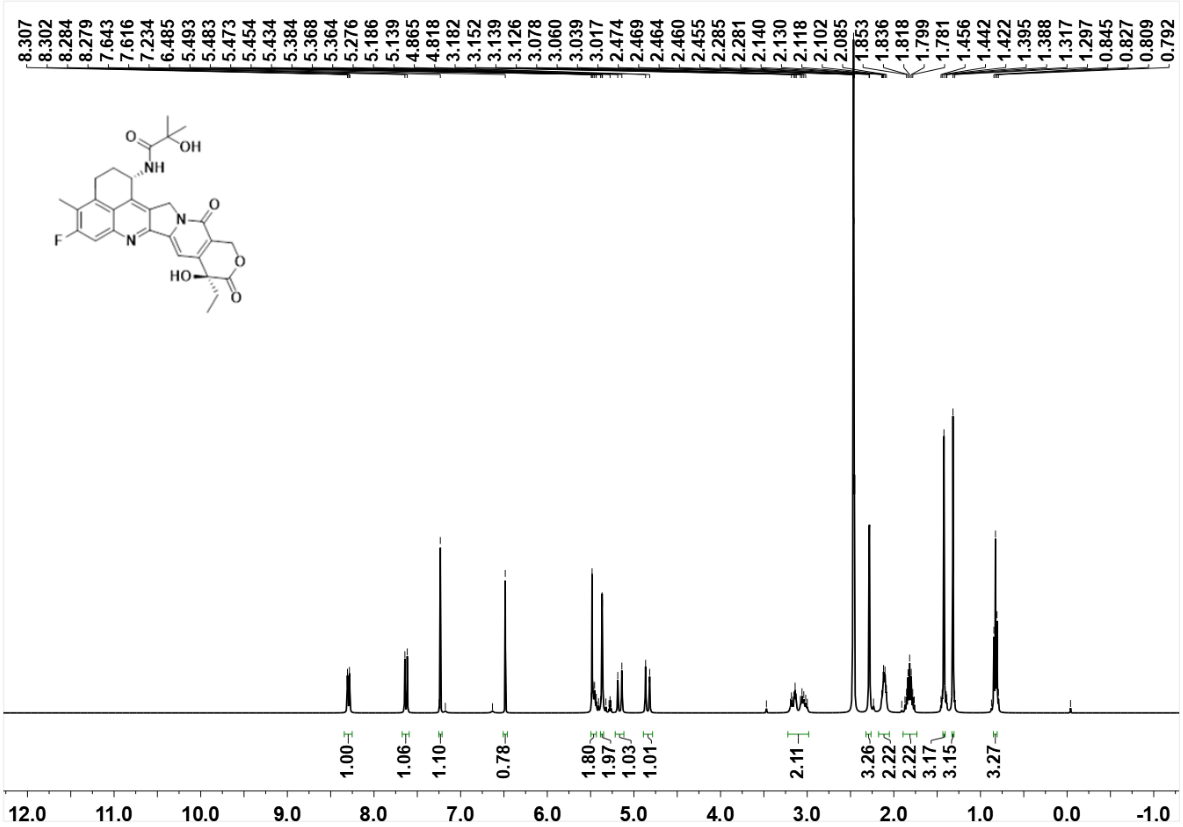

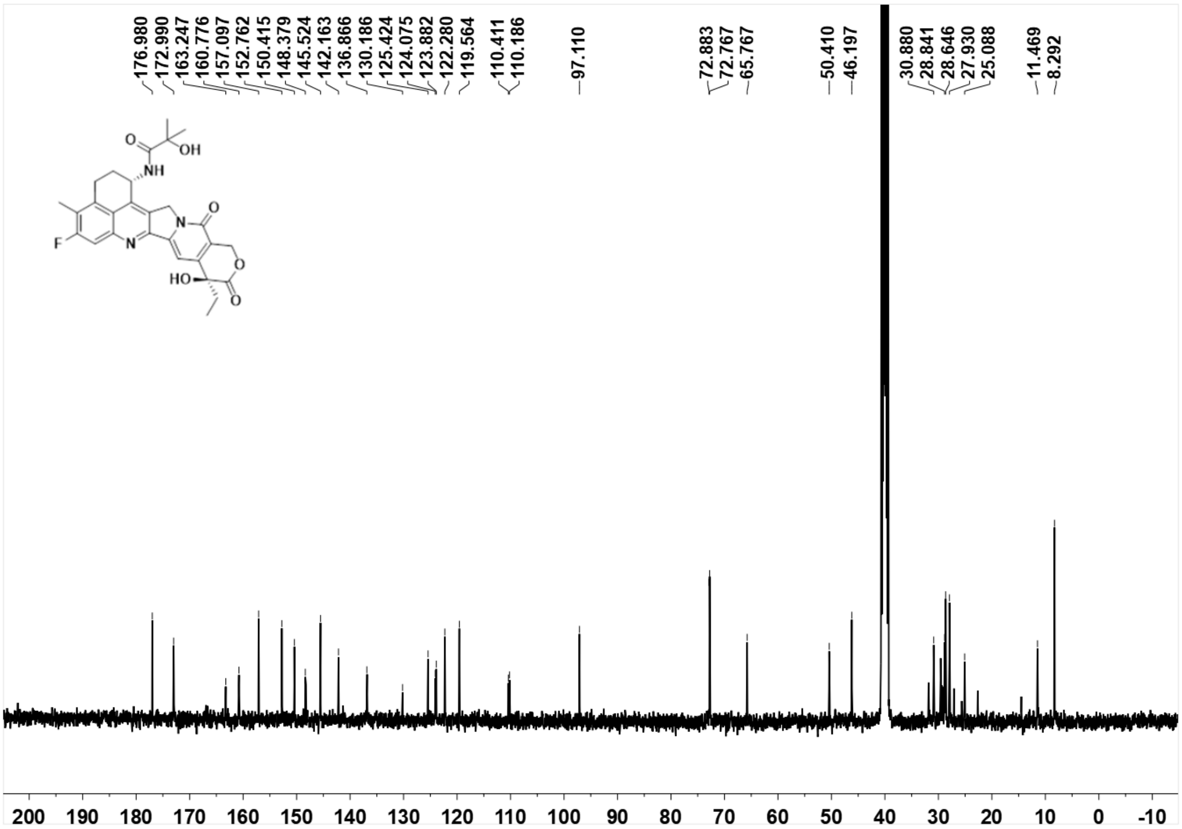

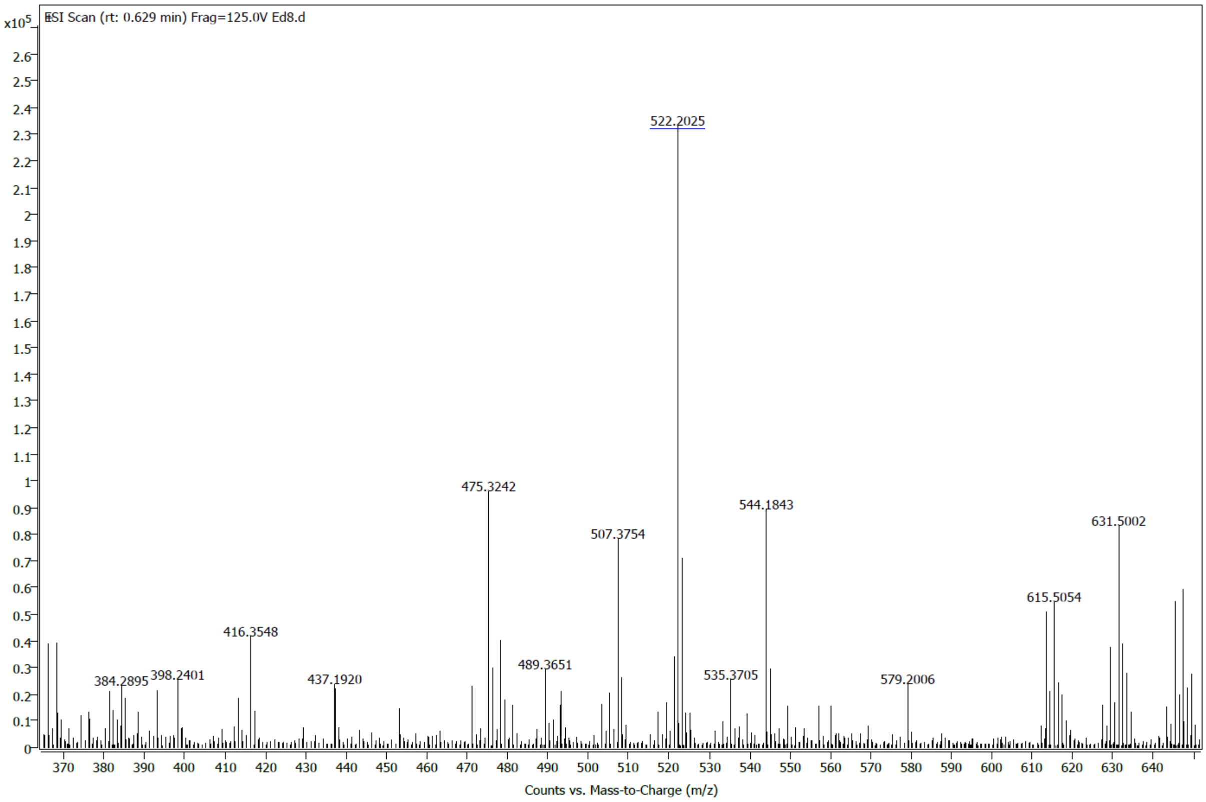

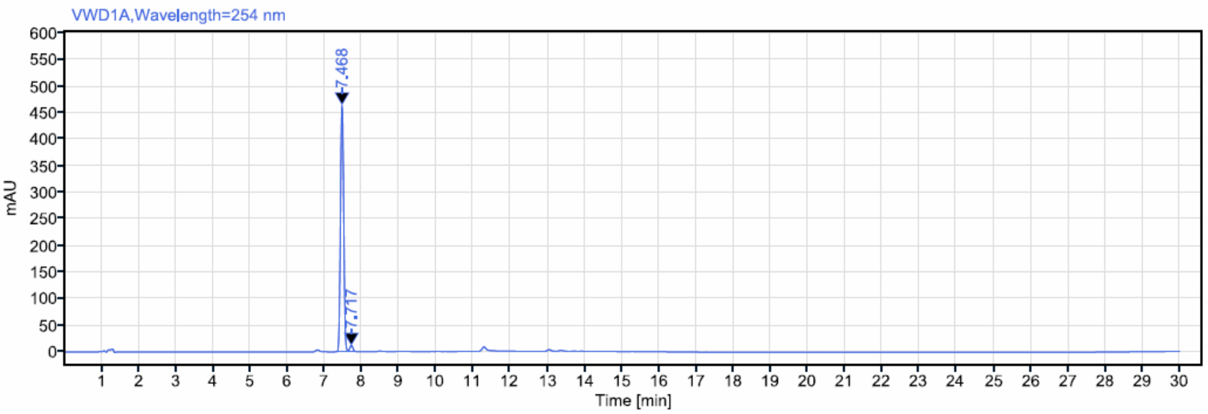

Signal: VWD1A,Wavelength=254 nm

RT [min]	Type	Width [min]	Area	Height	Area%	Symmetry
7.468	BV	0.34	2748.896	464.84	97.397	0.93
7.717	VB	0.23	73.459	12.00	2.603	0.94
		Sum	2822.354			

The spectrum of compound **Ed9**

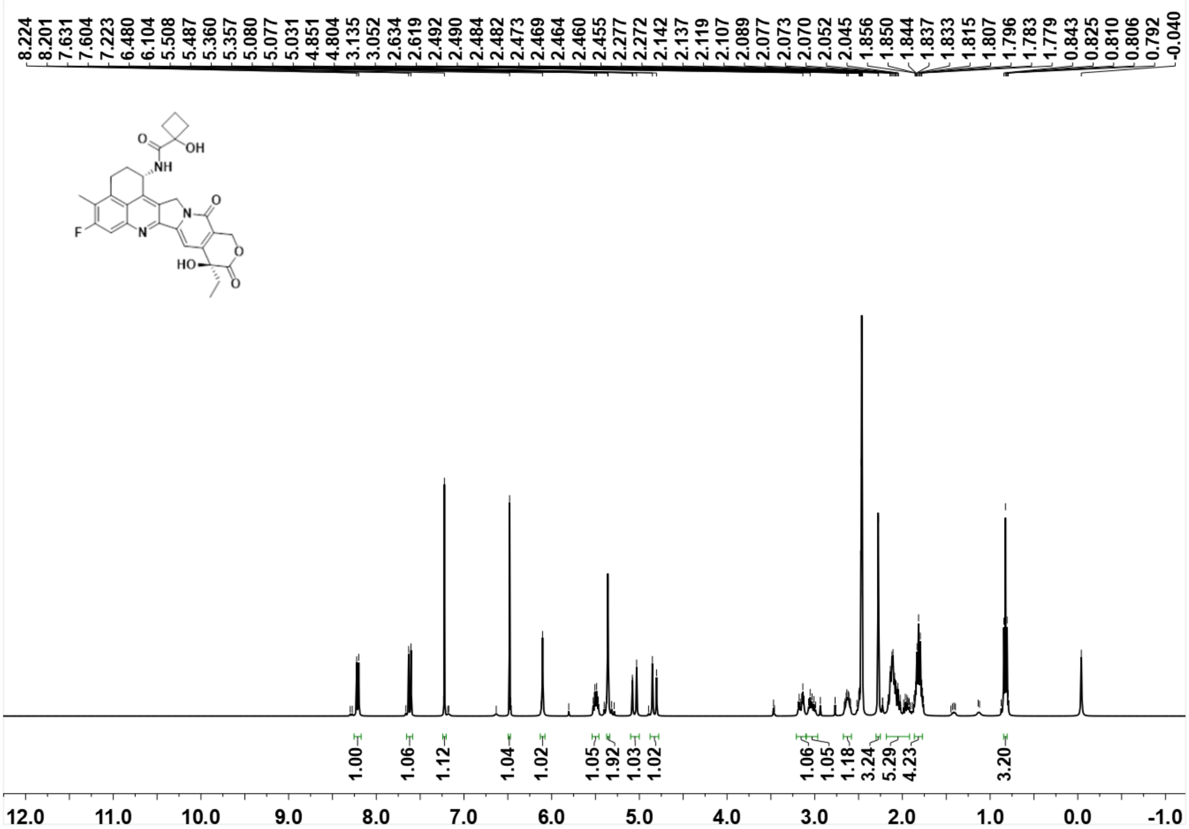

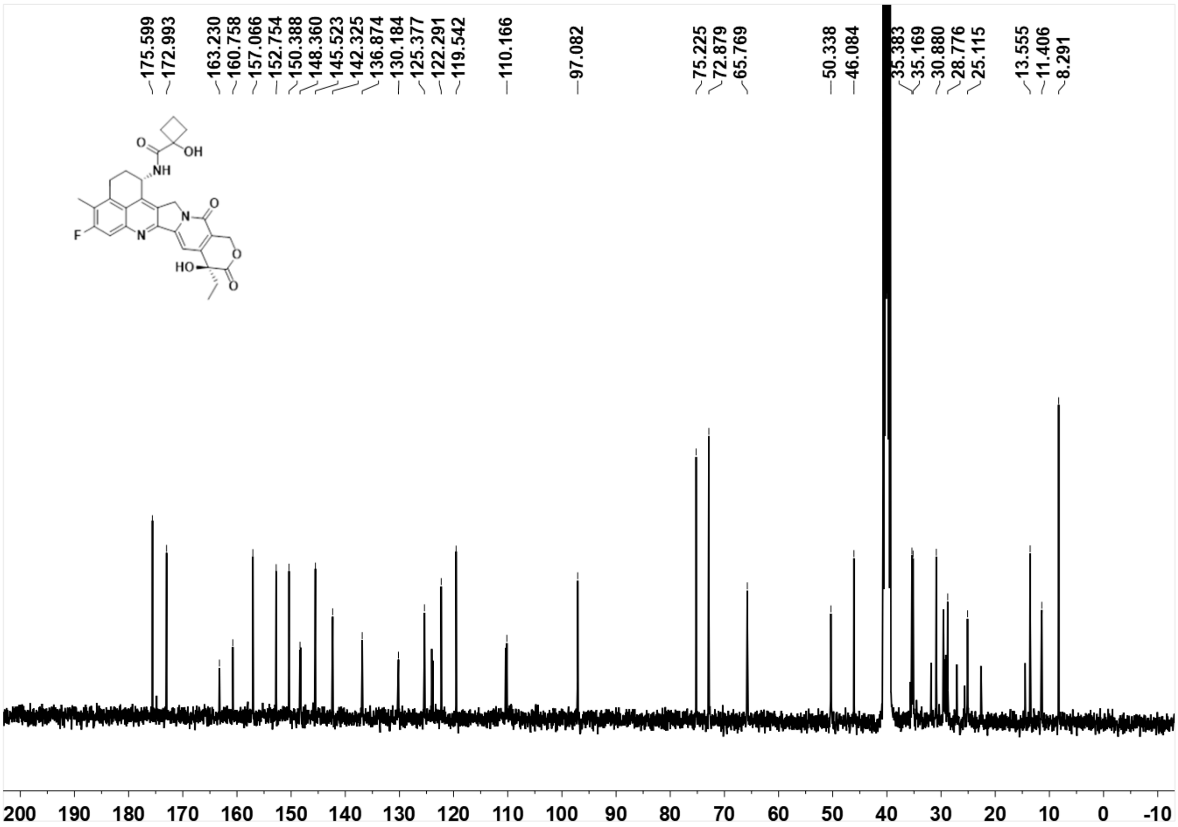

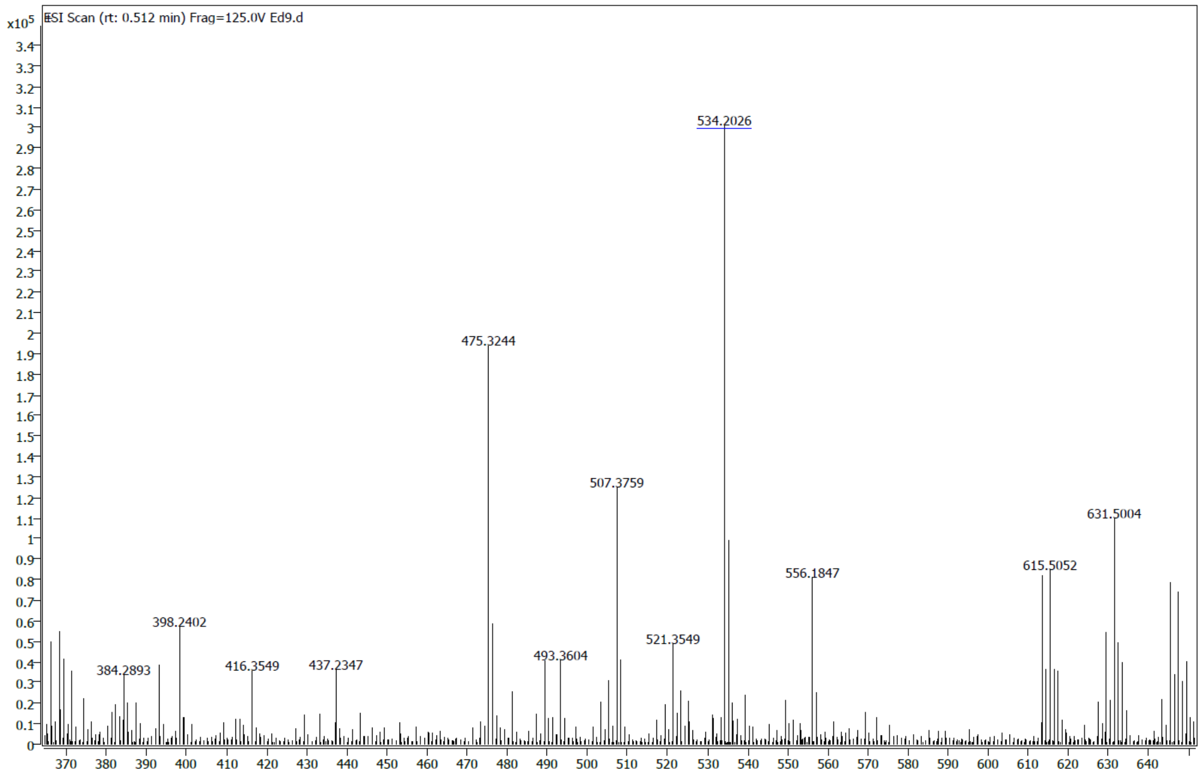

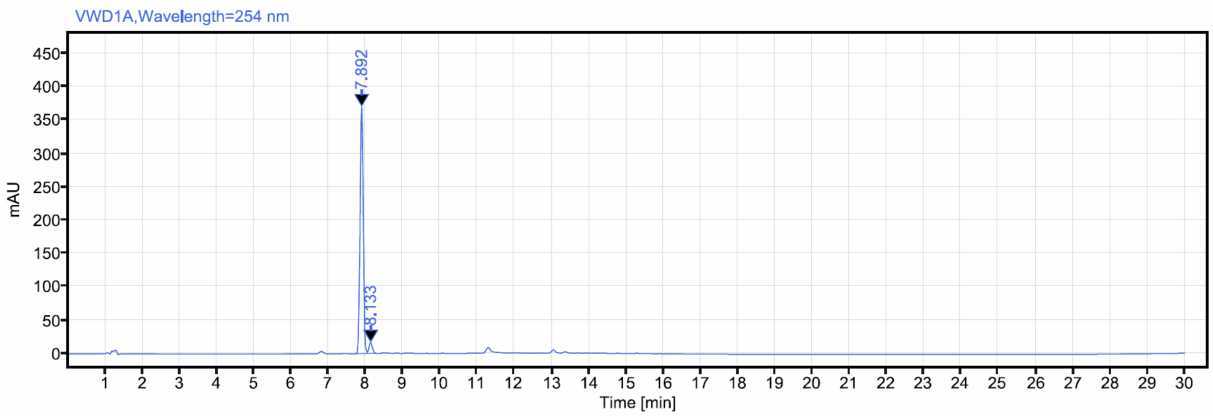

Signal: VWD1A,Wavelength=254 nm

RT [min]	Type	Width [min]	Area	Height	Area%	Symmetry
7.892	BV	0.33	2267.947	371.22	95.518	0.94
8.133	VB	0.28	106.414	16.75	4.482	0.91
		Sum	2374.361			

The spectrum of compound **Ed10**

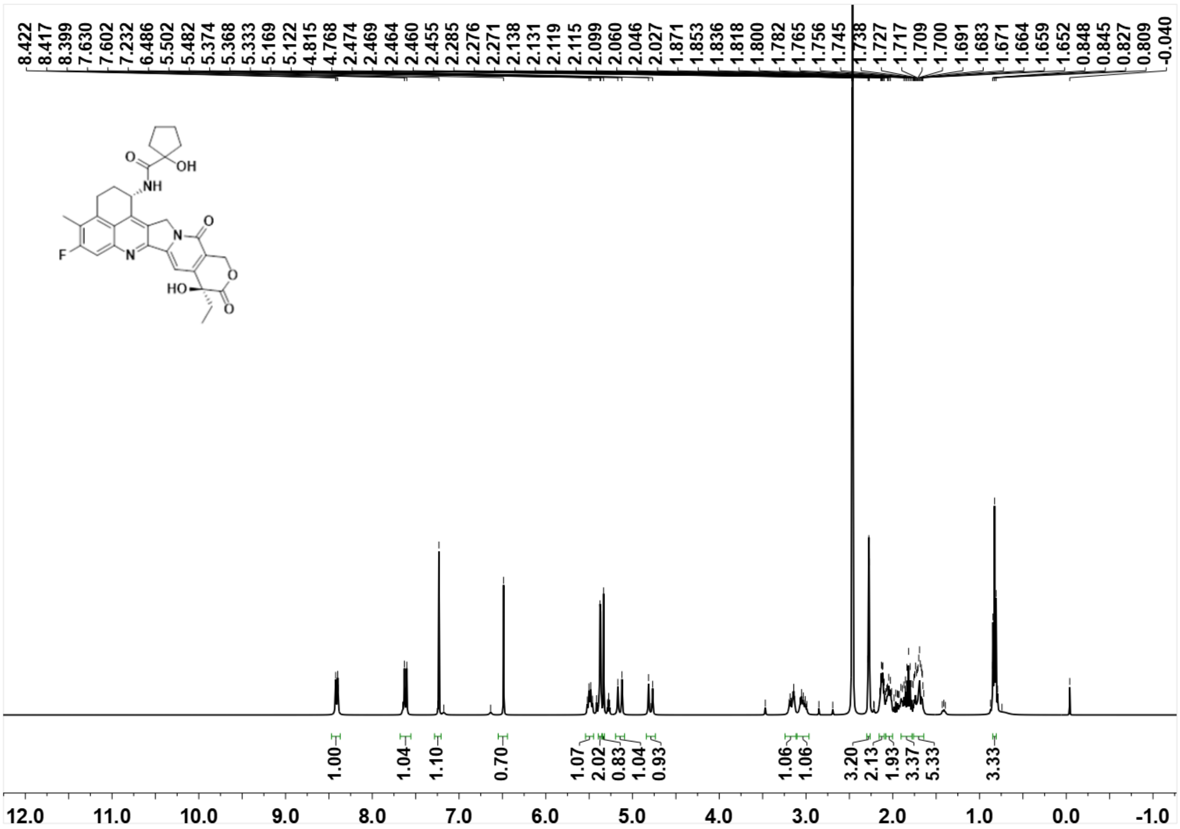

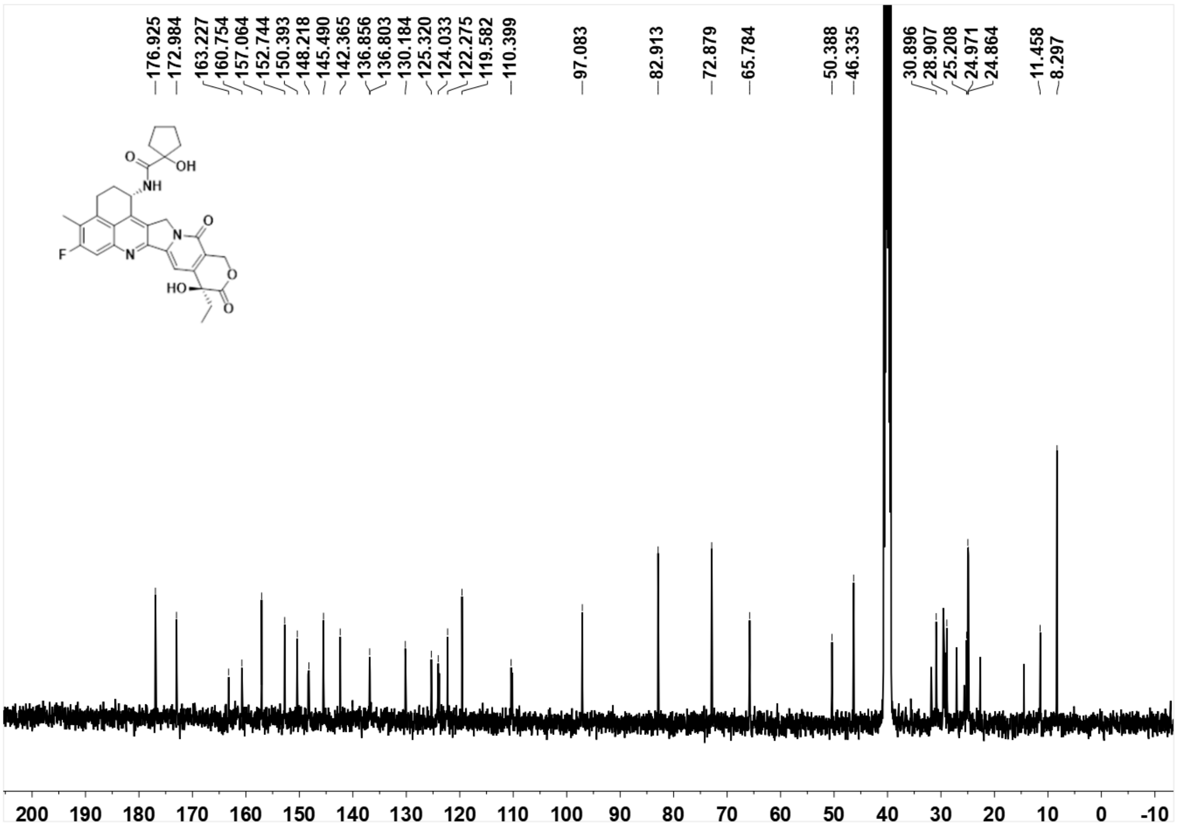

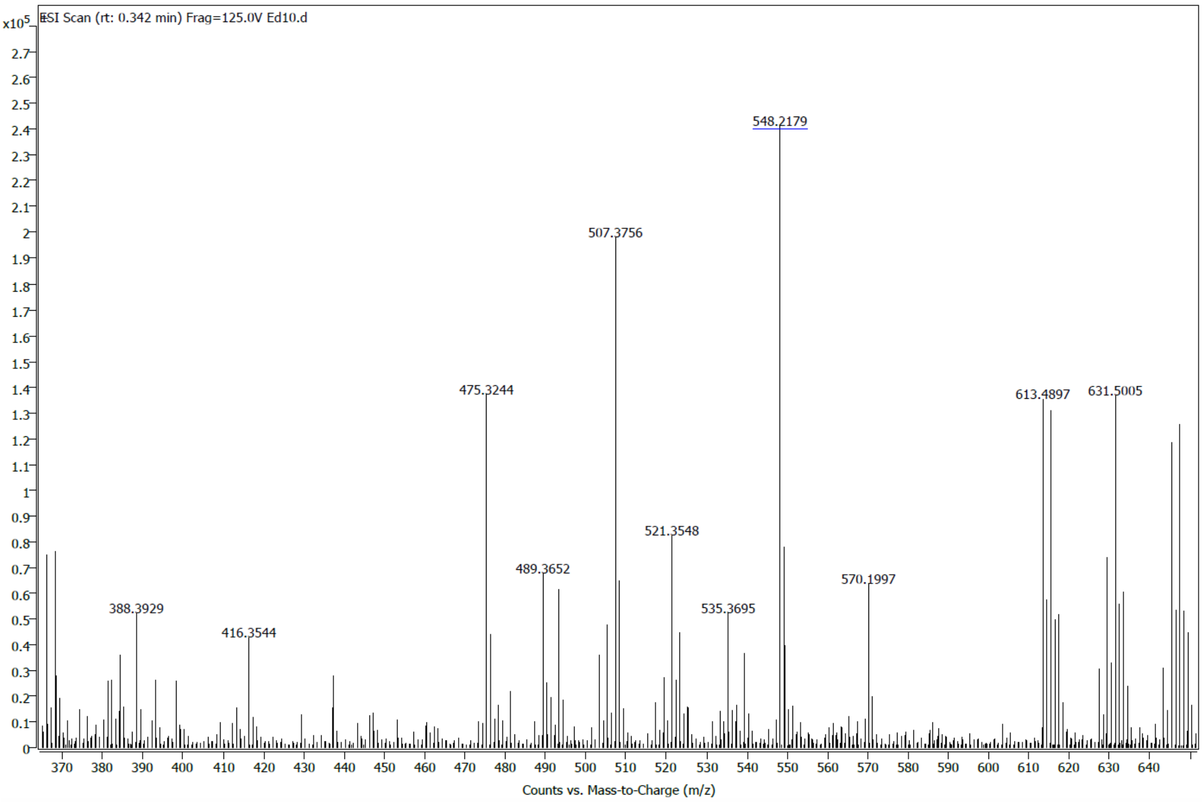

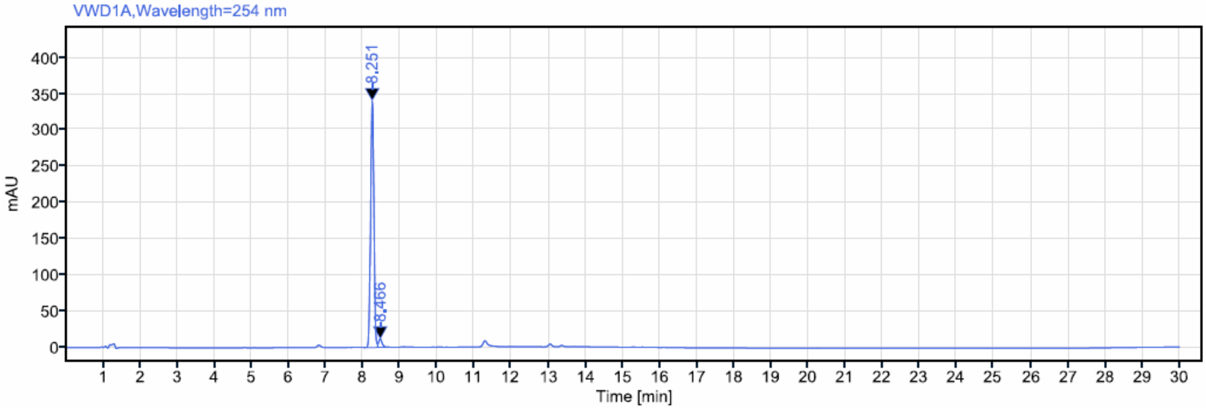

Signal: VWD1A,Wavelength=254 nm

RT [min]	Type	Width [min]	Area	Height	Area%	Symmetry
8.251	BV	0.35	2142.936	341.14	96.291	0.94
8.466	VB	0.47	82.536	11.73	3.709	0.76
		Sum	2225.472			

The spectrum of compound **Ed11**

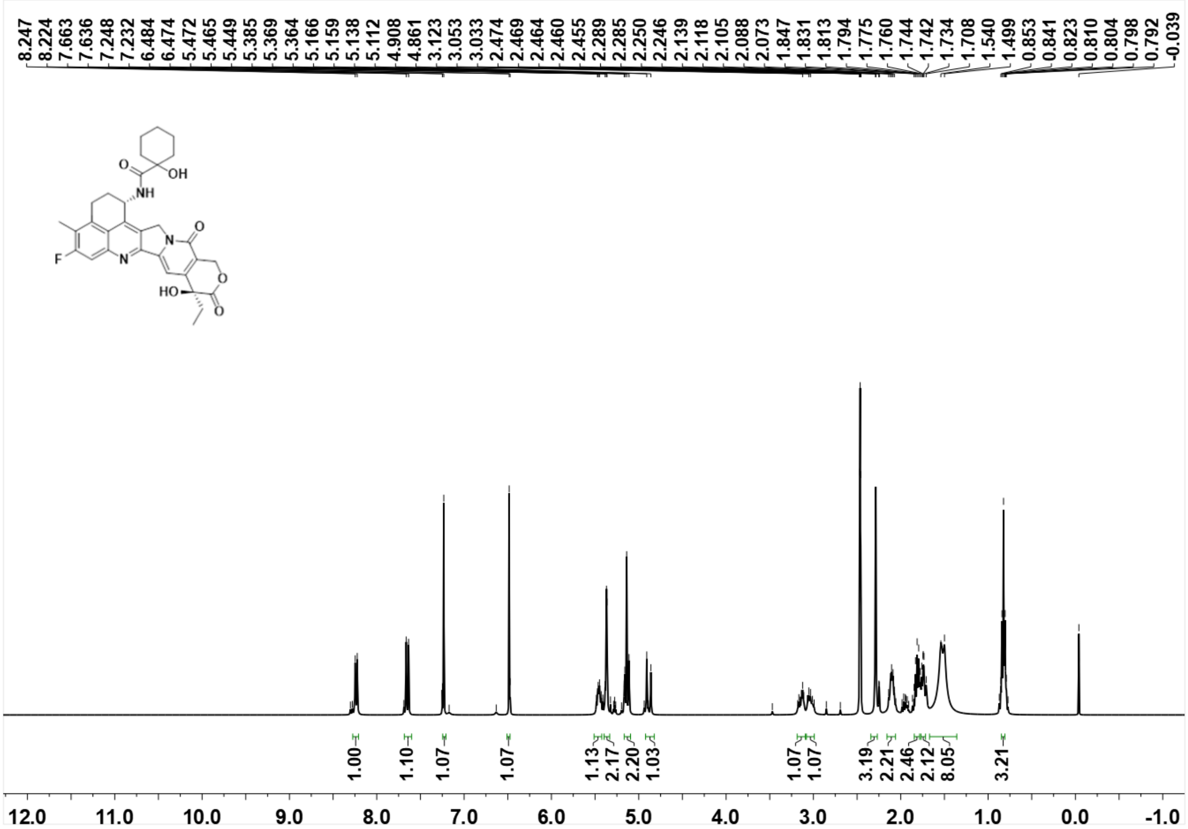

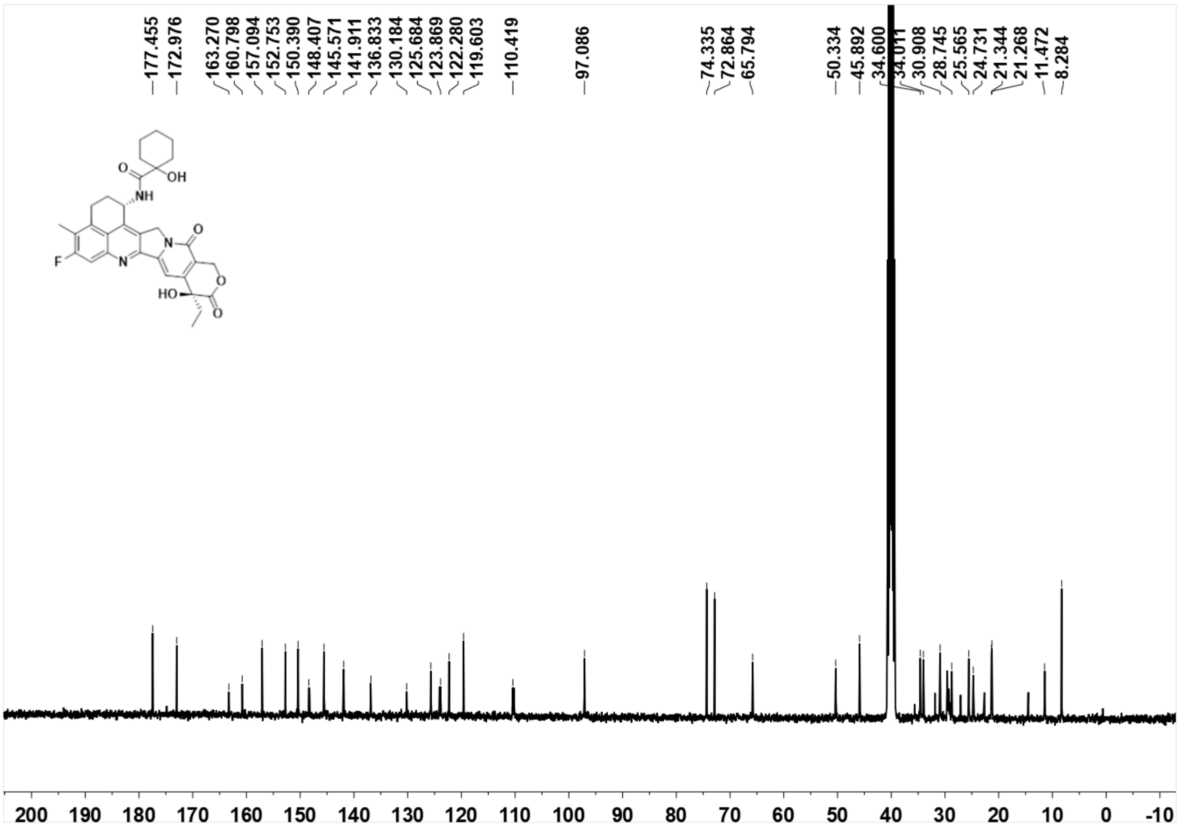

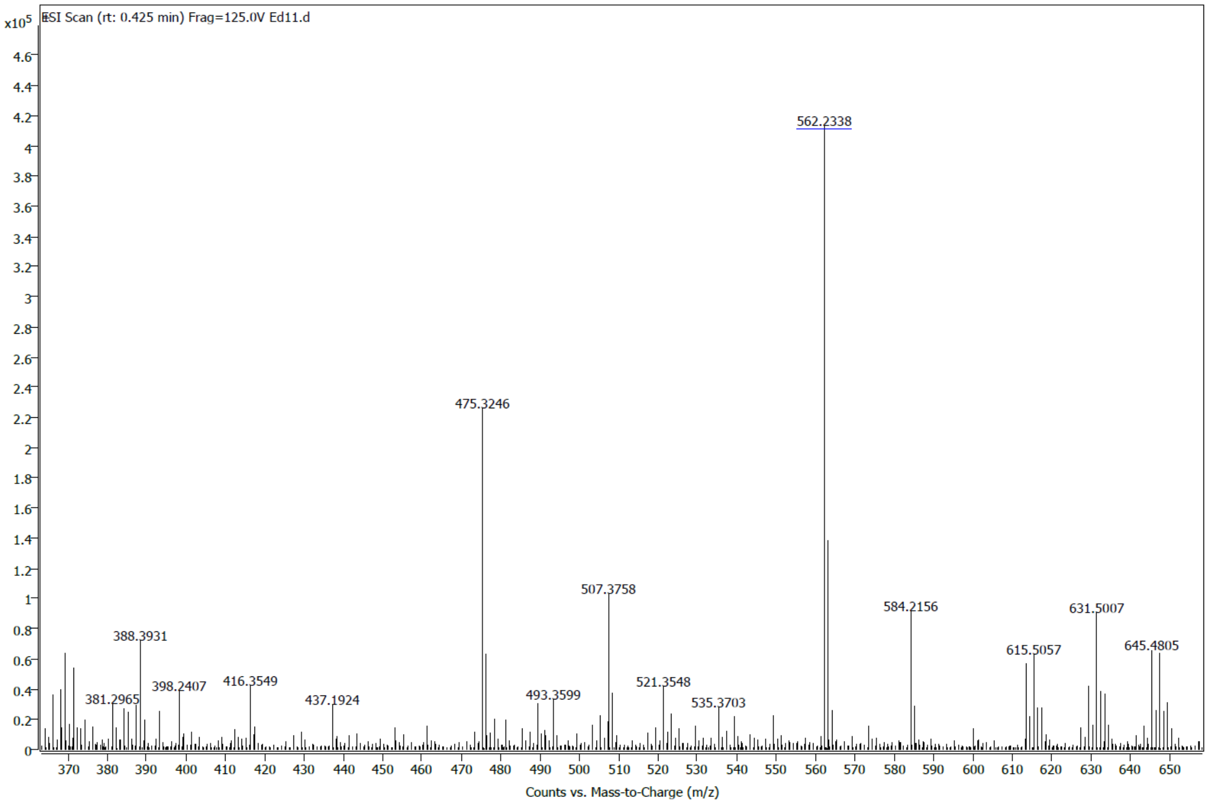

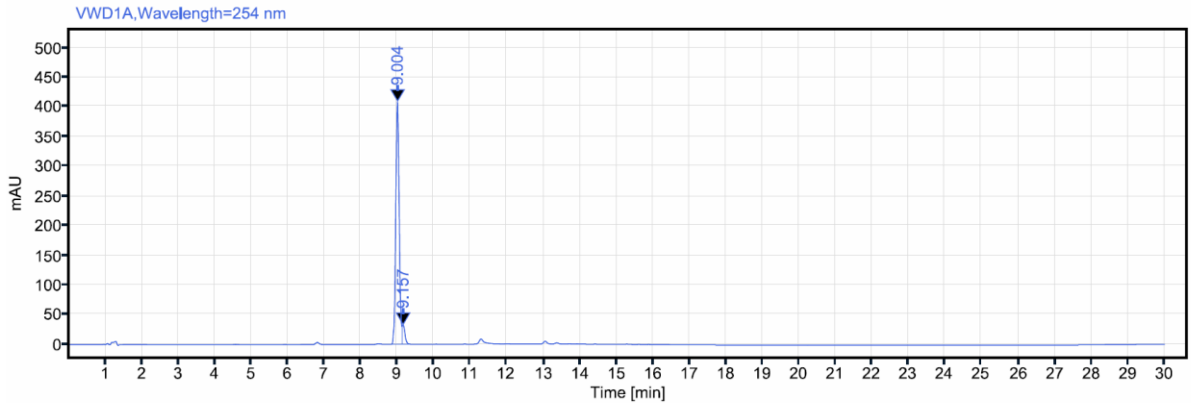

Signal: VWD1A,Wavelength=254 nm

RT [min]	Type	Width [min]	Area	Height	Area%	Symmetry
9.004	BV	0.32	2727.834	408.85	93.675	0.96
9.157	VB	0.45	184.184	32.77	6.325	0.44
		Sum	2912.018			

The spectrum of compound **Ed12**

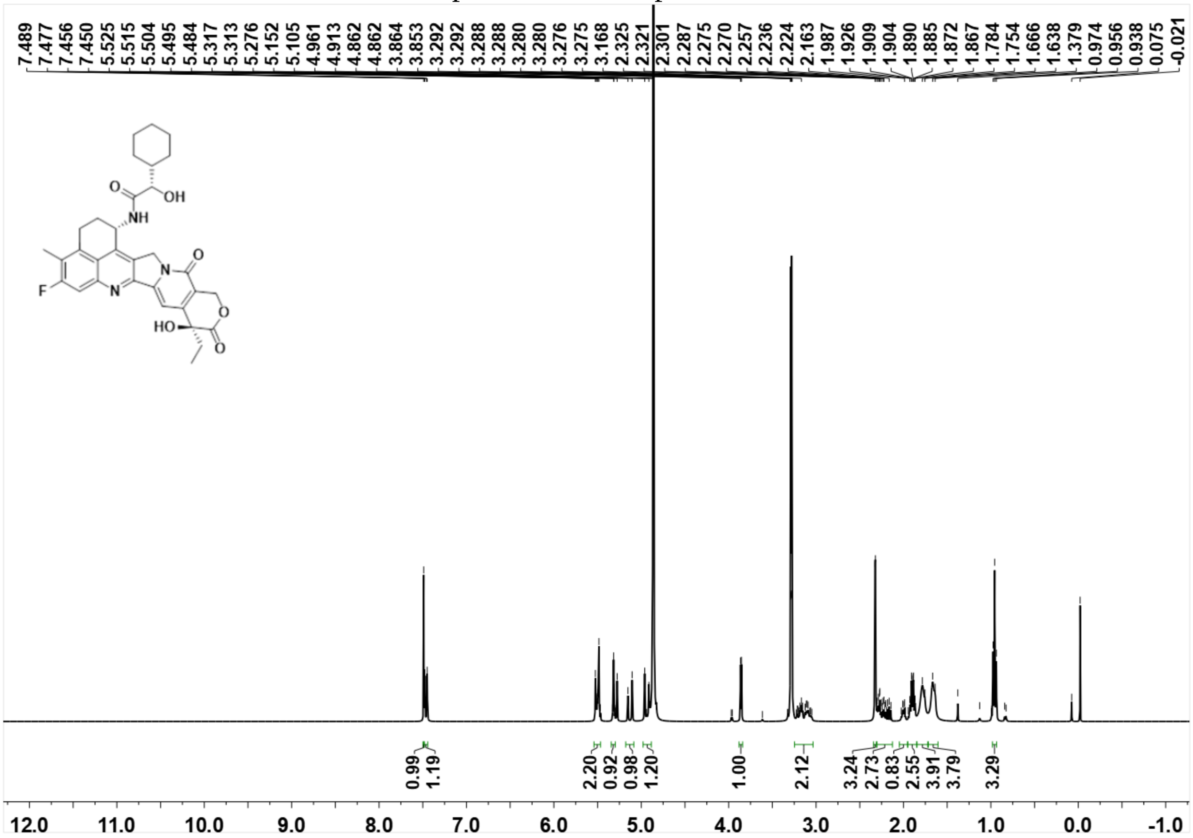

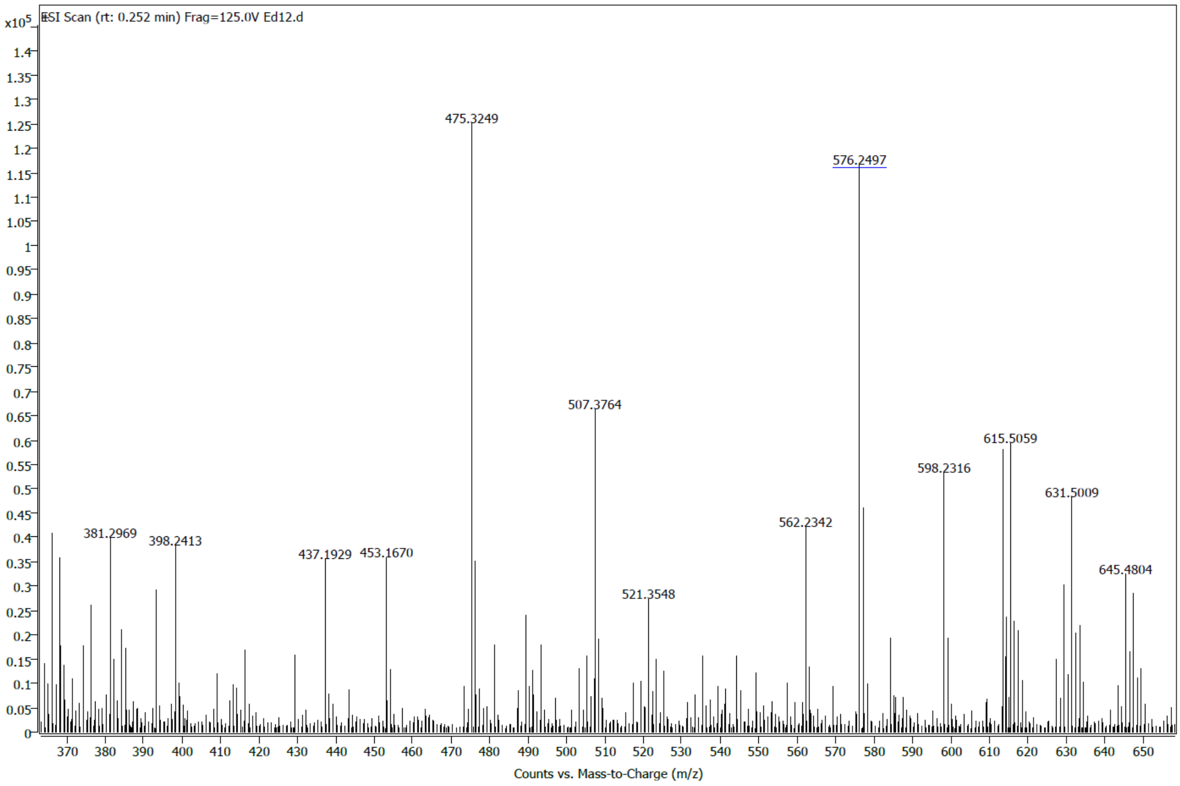

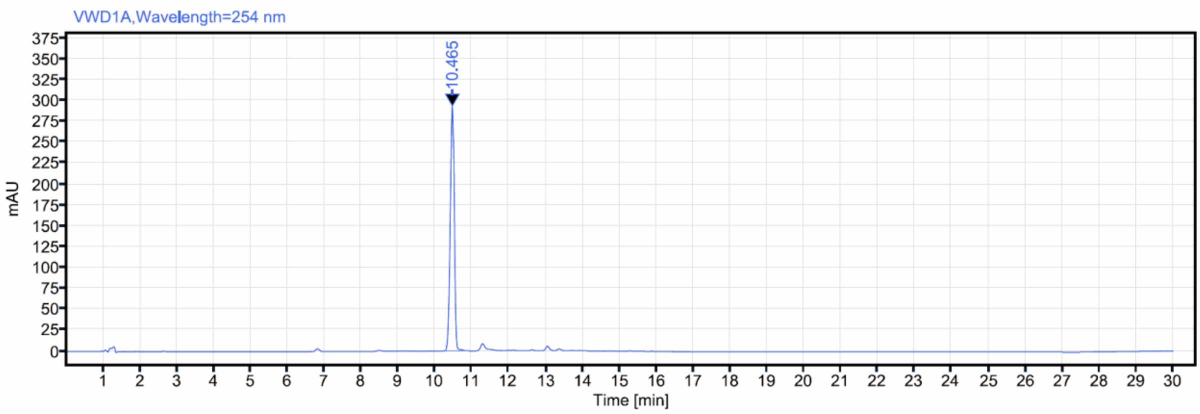

Signal: VWD1A,Wavelength=254 nm

RT [min]	Type	Width [min]	Area	Height	Area%	Symmetry
10.465	BB	0.75	2233.191	292.67	100.000	1.12
		Sum	2233.191			

The spectrum of compound **Ed13**

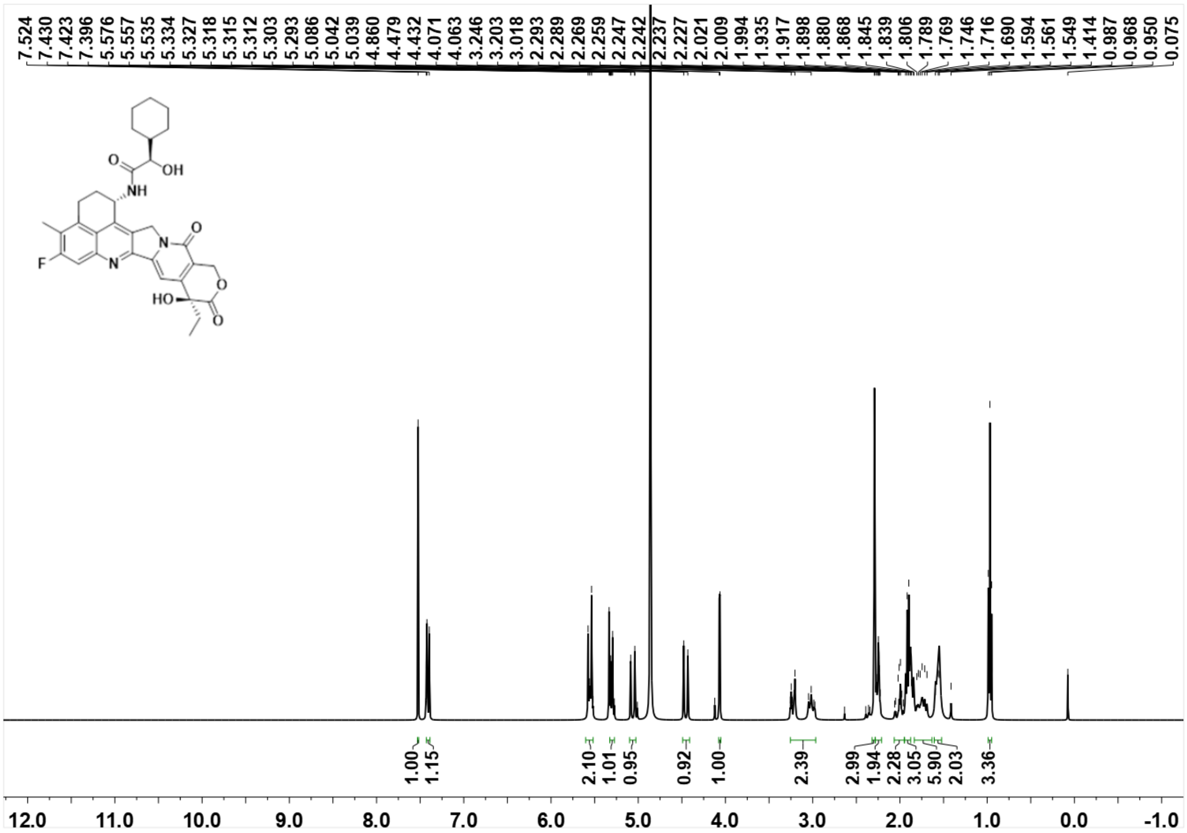

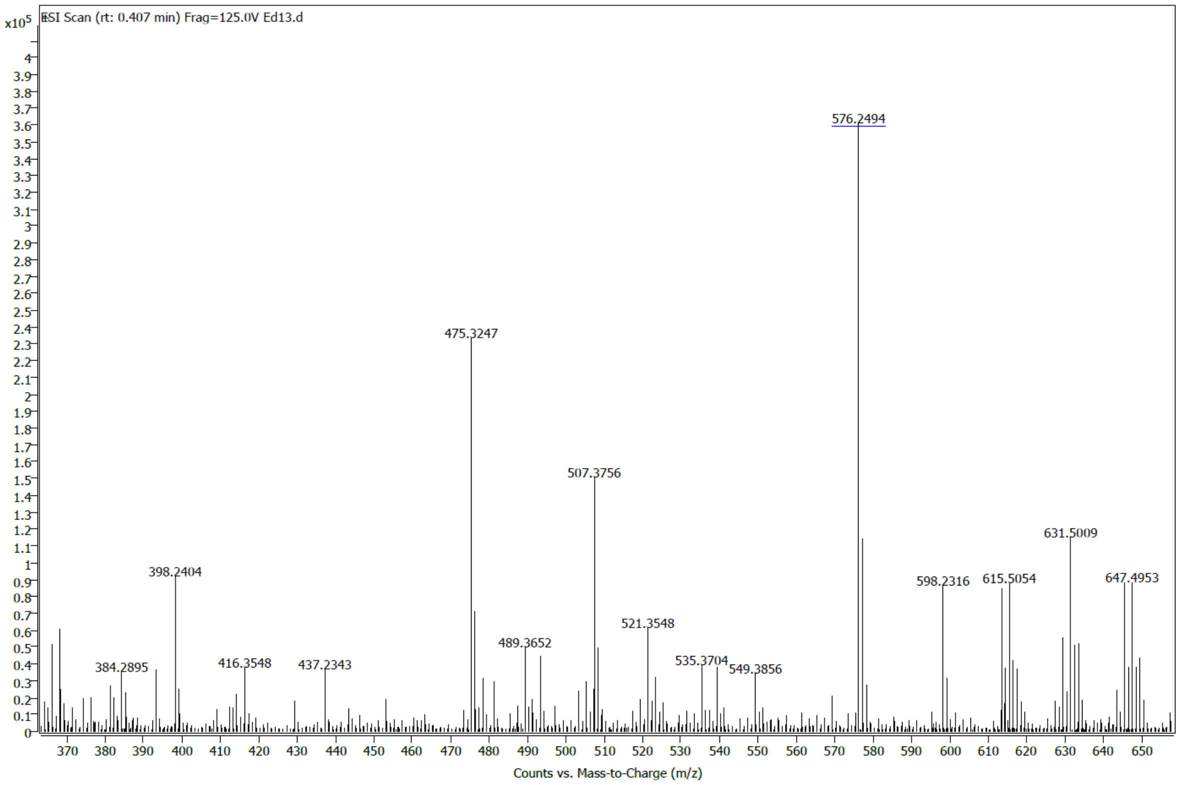

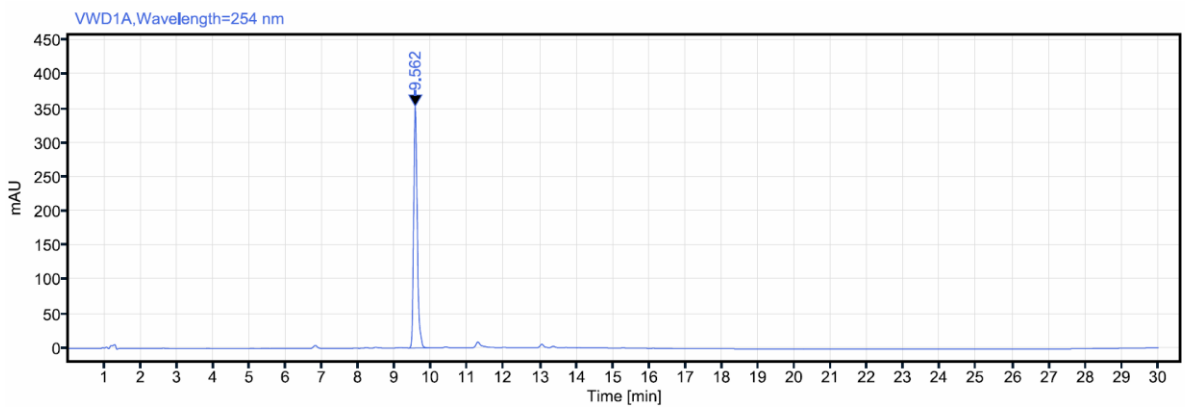

Signal: VWD1A,Wavelength=254 nm

RT [min]	Type	Width [min]	Area	Height	Area%	Symmetry
9.562	BB	0.57	2453.322	352.85	100.000	0.83
		Sum	2453.322			

The spectrum of compound **MC-GGFG-Ed9**

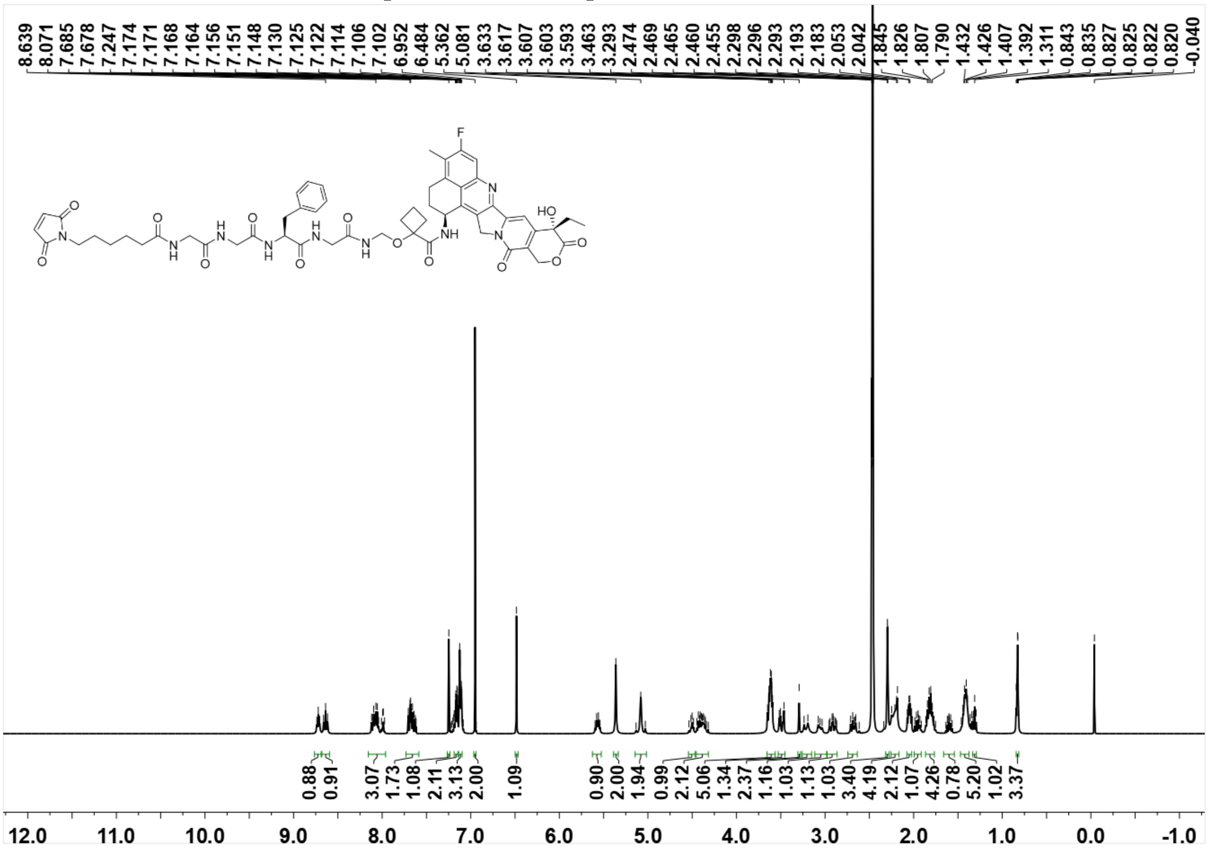

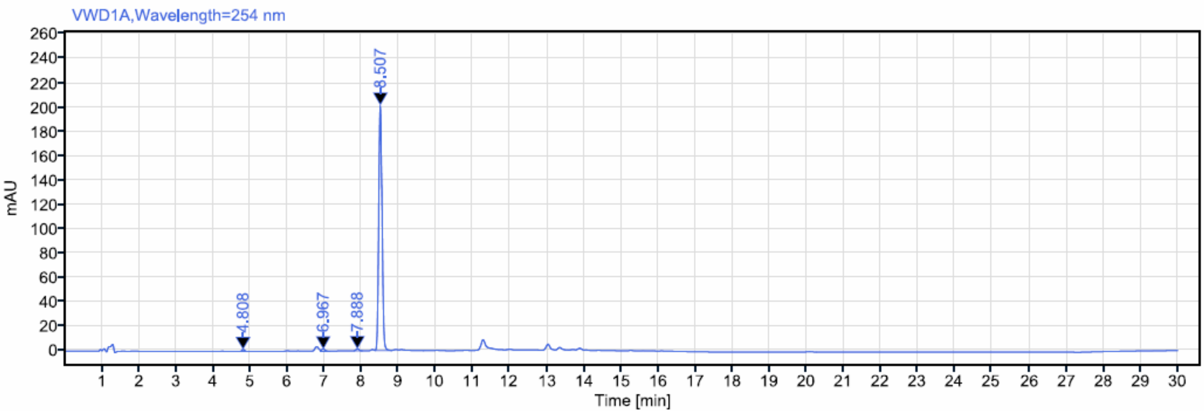

Signal: VWD1A,Wavelength=254 nm

RT [min]	Type	Width [min]	Area	Height	Area%	Symmetry
4.808	BB	0.28	8.487	1.76	0.663	0.87
6.967	VB	0.23	10.904	1.80	0.851	0.86
7.888	BB	0.24	10.594	1.80	0.827	0.87
8.507	VB	0.45	1250.622	201.57	97.658	0.92
		Sum	1280.608			

The spectrum of compound **MC-VA-DXd**

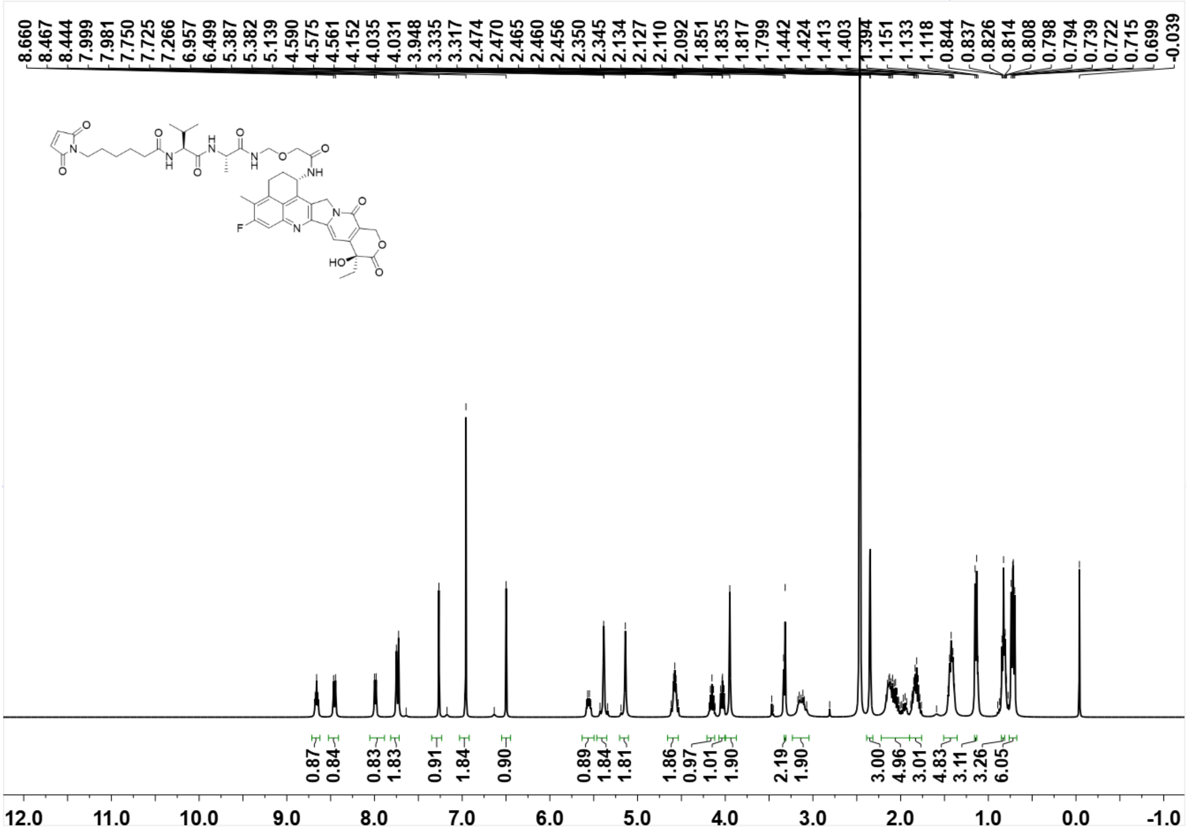

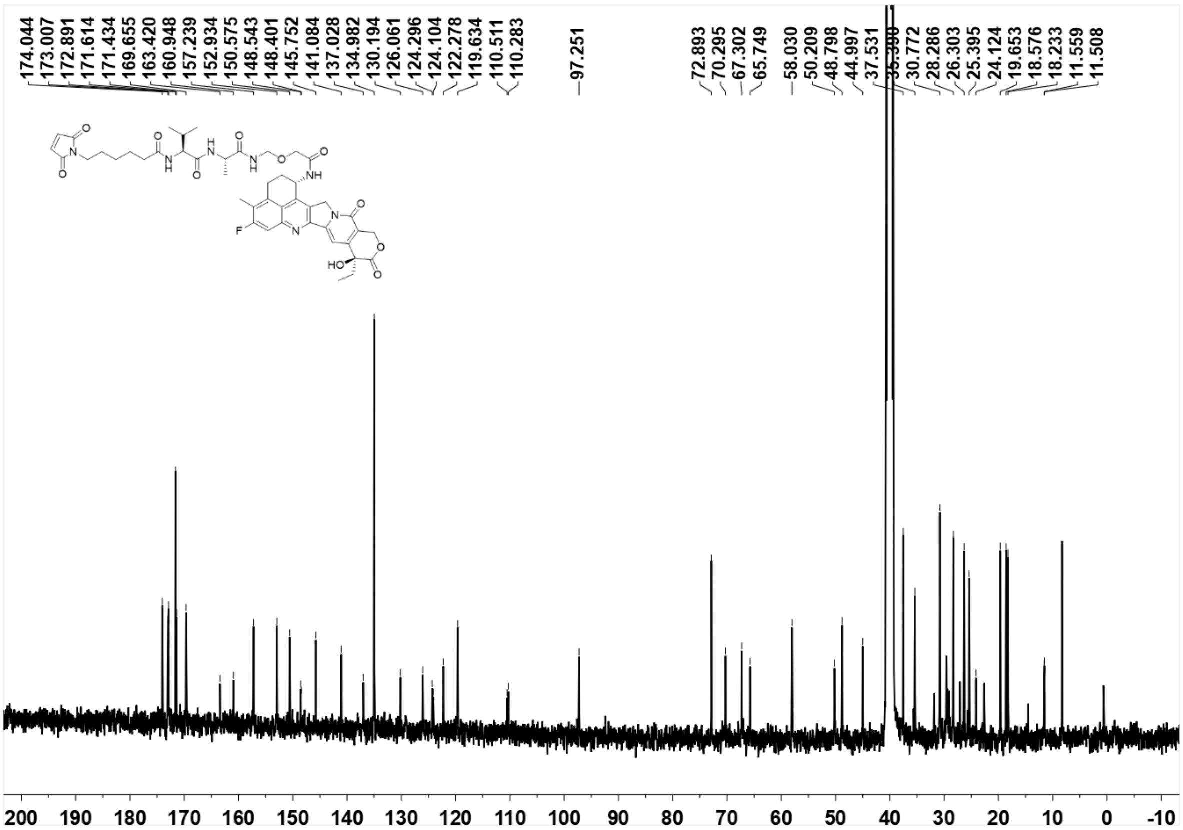

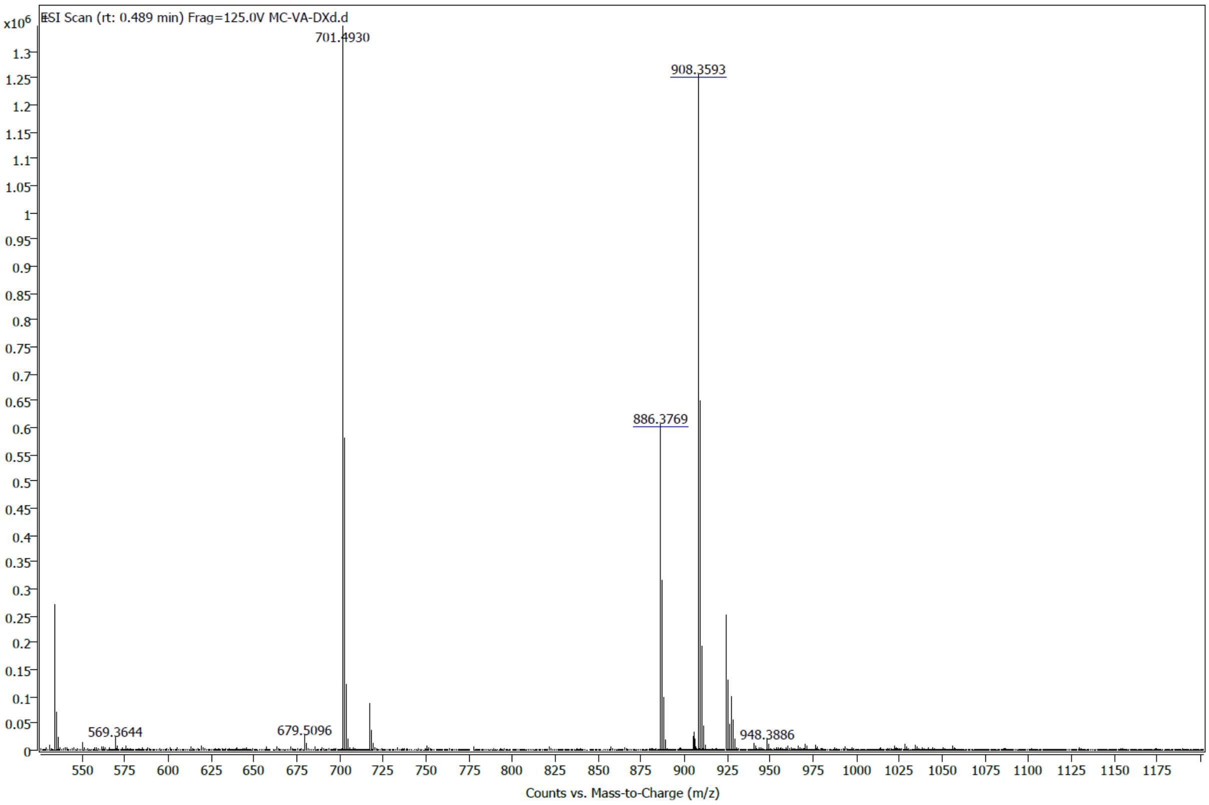

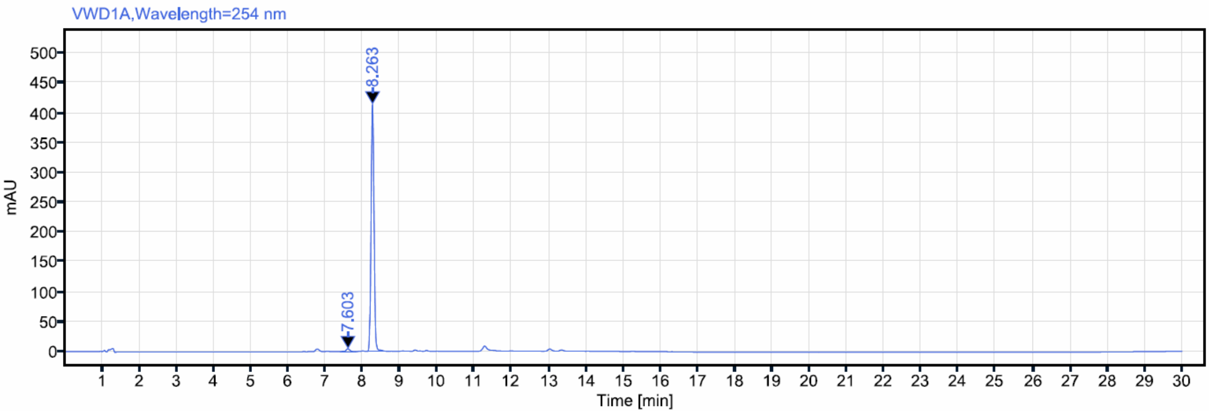

Signal: VWD1A,Wavelength=254 nm

RT [min]	Type	Width [min]	Area	Height	Area%	Symmetry
7.603	BB	0.43	35.668	4.93	1.487	1.14
8.263	VB	0.59	2363.499	414.89	98.513	0.89
		Sum	2399.167			

The spectrum of compound **MC-VA-Ed9**

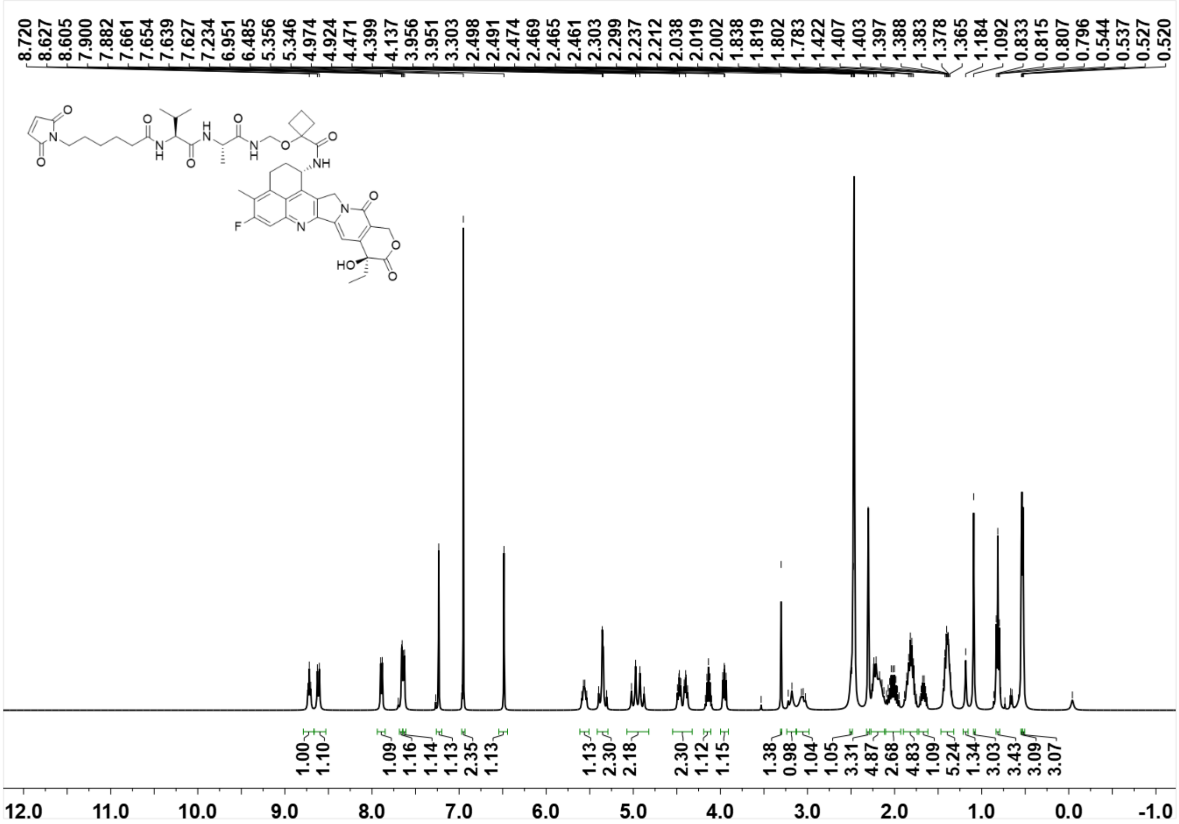

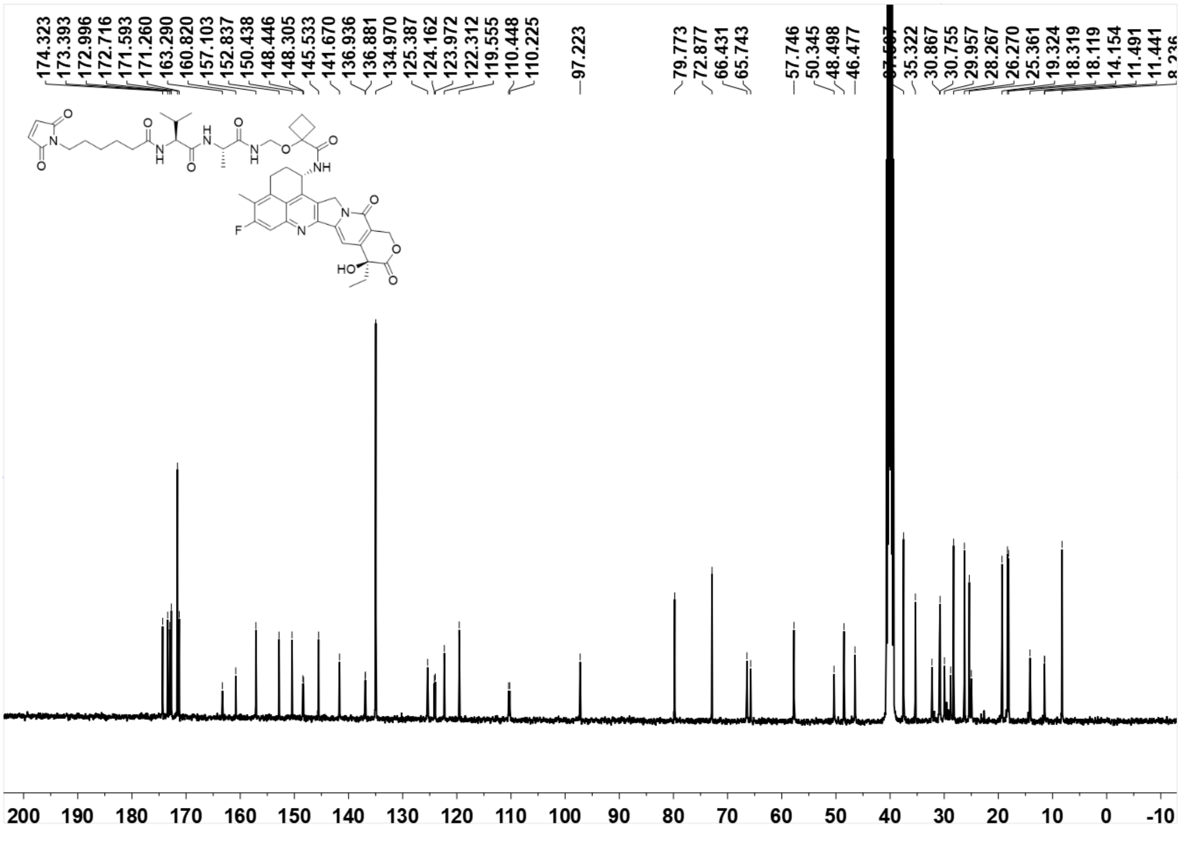

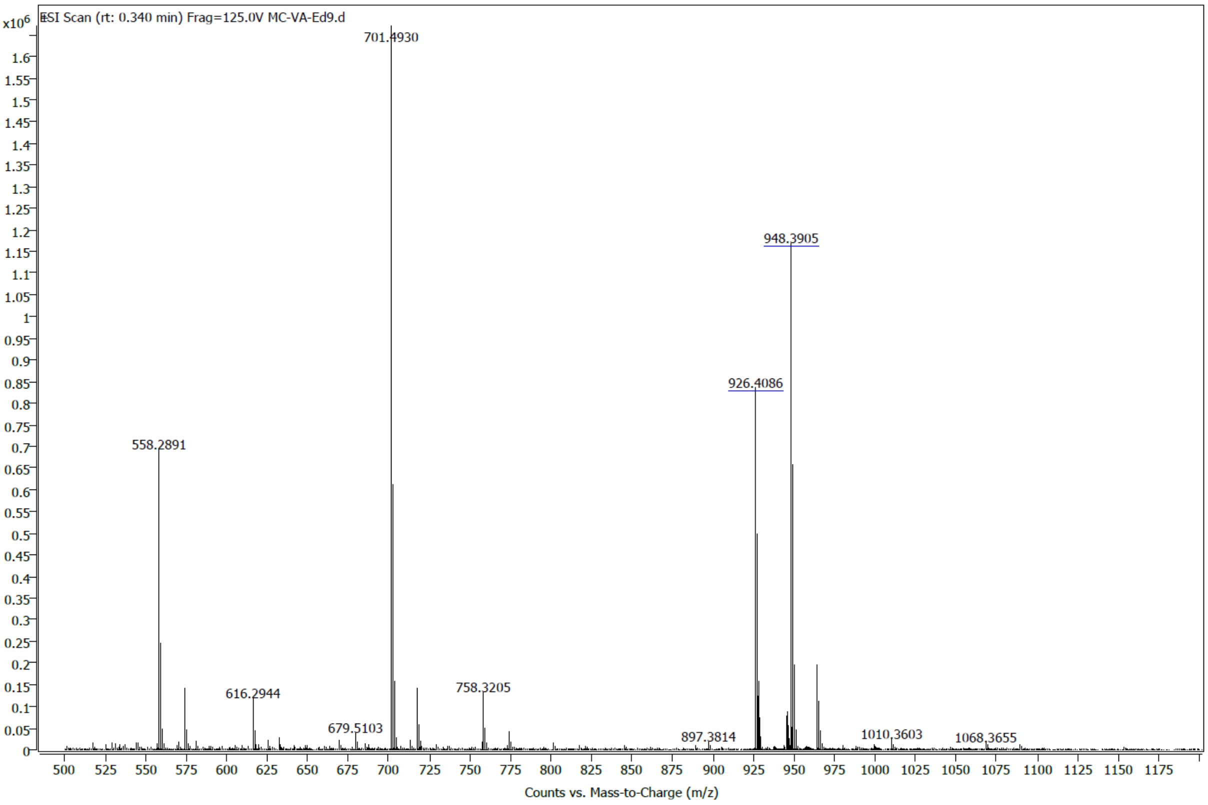

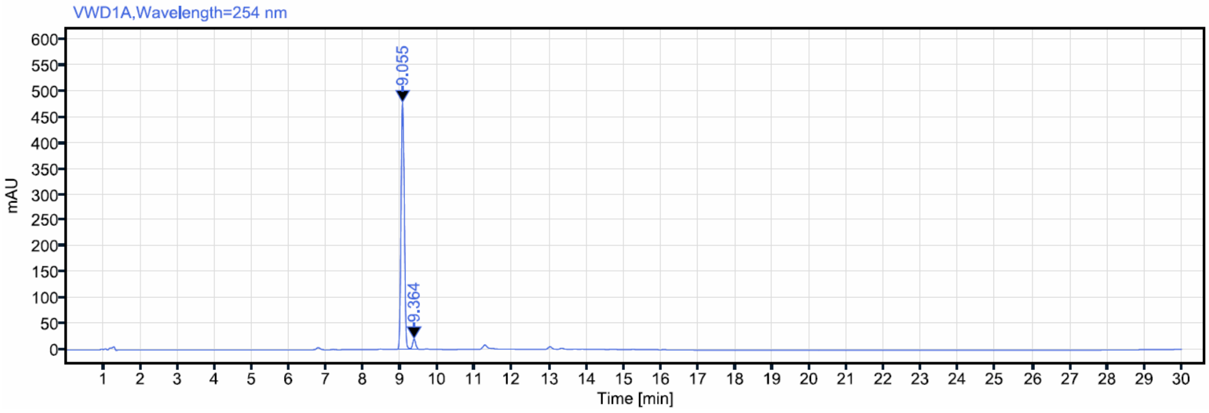

Signal: VWD1A,Wavelength=254 nm

RT [min]	Type	Width [min]	Area	Height	Area%	Symmetry
9.055	BV	0.42	2880.320	478.19	95.994	0.94
9.364	VB	0.25	120.196	19.81	4.006	0.94
		Sum	3000.516			

The HPLC spectrum of **blank injection**

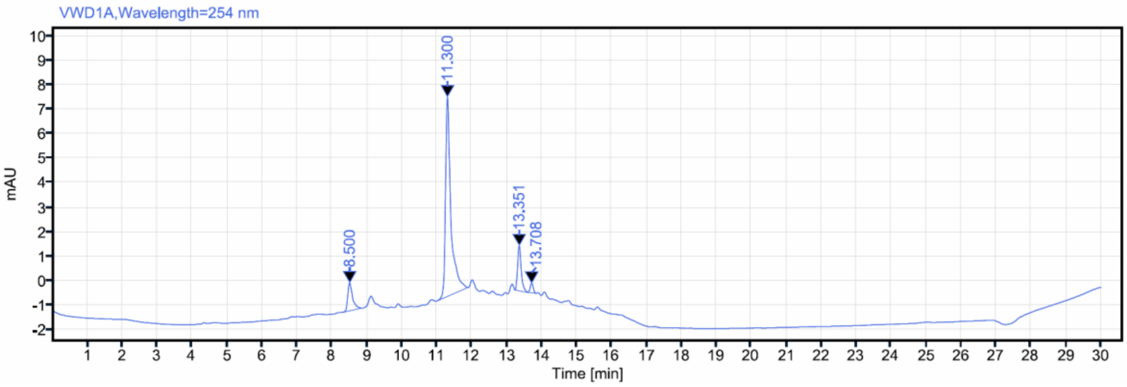

Supplementary References

[1] Y. Ogitani, K. Hagihara, M. Oitate, H. Naito, T. Agatsuma, *Cancer Sci* **2016**, *107*, 1039.

[2] E. Khera, C. Cilliers, S. Bhatnagar, G. M. Thurber, *Mol. Syst. Des. Eng.* **2018**, *3*, 73.

[3] K. Furuuchi, K. Rybinski, J. Fulmer, T. Moriyama, B. Drozdowski, A. Soto, S. Fernando, K. Wilson, A. Milinichik, M. L. Dula, K. Tanaka, X. Cheng, E. Albone, T. Uenaka, *Cancer Science* **2021**, *112*, 2467.

[4] M. M. C. van der Lee, P. G. Groothuis, R. Ubink, M. A. J. van der Vleuten, T. A. van Achterberg, E. M. Loosveld, D. Damming, D. C. H. Jacobs, M. Rouwette, D. F. Egging, D. van den Dobbelsteen, P. H. Beusker, P. Goedings, G. F. M. Verheijden, J. M. Lemmens, M. Timmers, W. H. A. Dokter, *Molecular Cancer Therapeutics* **2015**, *14*, 692.

[5] A. Pahl, C. Lutz, T. Hechler, *Drug Discovery Today: Technologies* **2018**, *30*, 85.
